# Selfish Pups: Weaning Conflict and Milk Theft in Free-Ranging Dogs

**DOI:** 10.1371/journal.pone.0170590

**Published:** 2017-02-08

**Authors:** Manabi Paul, Anindita Bhadra

**Affiliations:** Behaviour and Ecology Lab, Department of Biological Sciences, Indian Institute of Science Education and Research Kolkata, Mohanpur, West Bengal, India; University of British Columbia, CANADA

## Abstract

Parent-offspring conflict theory predicts the emergence of weaning conflict between a mother and her offspring arising from skewed relatedness benefits. Empirical observations of weaning conflict have not been carried out in canids. In a field-based study on free-ranging dogs we observed that nursing/suckling bout durations decrease, proportion of mother-initiated nursing bouts decrease and mother-initiated nursing/suckling terminations increase with pup age. We identified the 7^th^ - 13^th^ week period of pup age as the zone of conflict between the mother and her pups, beyond which suckling solicitations cease, and before which suckling refusals are few. We also report for the first time milk theft by pups who take advantage of the presence of multiple lactating females, due to the promiscuous mating system of the dogs. This behaviour, though apparently disadvantageous for the mothers, is perhaps adaptive for the dogs in the face of high mortality and competition for resources.

## Introduction

Maternal care in the form of nursing is an obligatory behaviour in mammals, at least in the early stages of development of the young. While in some species like hooded and harped seals, nursing occurs for a few days [1], in others it continues for months, and in the case of humans, can continue well beyond the natural weaning period [2]. Nursing is a highly energy intensive behaviour, and makes heavy demands on the maternal resources, with the mothers often losing weight substantially during nursing [3,4]. Mammalian species are typically iteroparous, so mothers need to strike a balance between their investments in current offspring and lifetime reproductive success. Parent-offspring conflict (POC) theory suggests the emergence of a zone of conflict over weaning between the mother and her offspring, due to skewed cost-benefit ratios from relatedness estimates [5]. Various models and empirical tests have validated this theory in contexts as diverse as brood size optimization, reproduction, resource sharing, mate choice, etc. [6,7]. POC in mammals can begin even before the offspring is born, as is evident in the case of preeclampsia (pregnancy induced hypertension) in humans, which is induced by the high energy demands of the fetus [8]. Weaning is a complex process, and it has been argued that weaning occurs through mutual agreement between the mother and offspring, rather than through a situation of conflict [9]. In polytocous species, within litter variations in suckling abilities and weight gain of the offspring add to the complications involved in understanding the dynamics of the weaning process.

Canids display a wide range of social systems, from solitary to strongly social habits, and are mostly polytocous. In the cooperatively breeding canids like wolves (*Canis lupus*) and coyotes (*Canis latrans*), pups receive care in the form of feeding of regurgitated food from adults other than their parents [10,11], which supplements their milk intake. Hence in such species the weaning process is not simply governed by mother-pup interactions. The domestic dog (*Canis familiaris*) is an interesting species of canid, which has evolved from pack living wolves through a process of domestication, and has adapted to life among humans. Pet dogs receive food from humans, and dietary supplementation by human caregivers is possible for the pups. A controlled study on pet dogs has suggested that weaning strategies might exist among pups [9] but the possibilities have not been explored further.

POC over extended parental care has been demonstrated in free-ranging dogs (*Canis familiaris*) [12,13]. Mothers begin competing over food with their pups during weaning; the conflict levels increasing with pup age [12,13]. This conflict is resource-dependent, mothers choosing to be more aggressive when richer resources are present, thereby showing preference towards lifetime reproductive success (LRS) over immediate benefits from the current litter [12]. Indian free-ranging dogs are scavengers dependent on human-generated wastes for their sustenance [14], and have existed in similar conditions for centuries, living in close proximity with humans, but not under their supervision [15]. They defend territories as groups, but typically prefer to forage singly, though capable of forming large packs to hunt [15,16]. They have a promiscuous mating system [17], with no reproductive skew within the groups, though allocare by adults is possible [18], but is not ubiquitous (Paul and Bhadra, Under review). This suggests a highly flexible social structure, as opposed to the more stringent social organization of pack living canids [19]. The free-ranging dog mothers face a challenging environment, with resource limitation, competition and high pup mortality [20], leading to low assured fitness per litter [21]. Striking a balance between investment per litter and LRS can provide greater fitness benefits to the mother, and eventually lead to higher stability for the population. Thus understanding the weaning strategies of free-ranging dogs can also help to test the implications of POC theory. We conducted a study on nursing behaviour in free-ranging dogs to understand if a zone of conflict over nursing/suckling exists between mothers and pups in dogs. If mothers tend to give precedence to LRS, then we expected to observe an increase in unsolicited suckling attempts of pups and decreased interest of the mothers to nurse them as they grew older.

## Materials and Methods

### Ethics statement

No dogs were harmed during this work. All work reported was observation based. The methods were approved by the IISER Kolkata animal ethics committee of (1385/ac/10/CPCSEA), and followed approved guidelines of animal rights regulations of the Government of India.

### Data collection

We observed 15 dog groups from the 3^rd^ to 17^th^ weeks of pup age, in West Bengal, India. Each dog group consisted of one or more adults and pups/juveniles (of both the sexes) that defended a common territory and shared resources like food and shelter. All individuals in the group were uniquely named according to their coat colour and patch patterns. A pedigree was maintained to check the relationships among the focal dogs of each group. A group could have more than one lactating female with her current litter and often these females were observed to be related with each other. Thus 22 mothers-litter units (having 22 mothers and 78 pups) were available from 15 dog groups (S1 Table) that were used as our focal units for behavioural observations. Pups are immobile and restricted to the dens closely guarded by mothers during the first two weeks after birth [22], so rigorous observations were impossible before the third week. Here we use “weeks” to refer to pup age throughout this paper. Each group was observed for two morning (0900-1200h) and two evening (1400-1700h) sessions over blocks of two weeks. Each observation session consisted of 18 instantaneous scans of one minute each and 18 all occurrences sessions (AOS) of five minutes each, intermingled in a random order. Each scan and AOS was followed by a mandatory two minutes break. Thus we had a total of 8712 scans and AOS each per mother-litter unit. Scans included instantaneous recordings of both behavioural states and events, whereas AOS included the detailed recordings of all behavioural events, with the identity of the initiator, and whenever applicable, that of the recipient [23].

Any form of behavioural interaction that could increase the chances of pup survival (such as nursing, allo-grooming, regurgitation of food, food offering or food provisioning, den cleaning, pile sleep, pup guarding and play) were recorded as care. For 11 of the mother-litter units, in addition to the scans and AOS, we recorded the details of all nursing/suckling bouts recorded during the observation period. A combination of the initiator’s identity and the duration of nursing/suckling bouts was used to estimate the interest of mothers to offer nursing (hence cooperation), and pups to receive suckling. Both termination of nursing bouts by the mother and failed solicitations by pups were considered as refusal to nurse (hence conflict), while termination of suckling by the pups was considered voluntary. Often females of a group produce litters at different times that facilitates allosuckling (suckling from lactating females other than the mother). We recorded the details of all allosuckling bouts too. The free-ranging dogs experience quite high rates of mortality (81% of the total pups born) in the early stages of their lives [20], and thus the litter size often changes as the pups grow, reducing from the litter size at birth. So we considered the current litter size for every week of observations in the analyses.

### Statistics

We used the data from instantaneous scans to estimate the proportion of time spent by the mother in maternal care behaviours out of the total time for which the focal animals were observed. The data from AOS were used to estimate the rate (frequency per hour) of nursing/suckling (henceforth designated as suckling) at different pup ages. From the detailed records of suckling bouts of 11 mother-litter units, we measured the duration of each bout and calculated the proportion of mother and pup initiated suckling bouts over pup ages. For statistical analysis, we used StatistiXL 1.10, Statistica 12 and R (“lme4” package)[24,25]. To check whether the identity of the suckling initiator (initiator), pup age in weeks (age) and the current litter size (LS) had any effect on the rate of care received by the individual pup in terms of suckling, we ran a generalized linear mixed effect model (GLMM). We incorporated the predictor variables (initiator, age and LS) as the fixed effects and the identity of mother-litter units as the random effect. We started with the full model, i.e., with all possible three and two-way interactions among the fixed effects. If the three or two-way interaction was non-significant then we reduced the model using the standard protocol of backward selection method and ended up with the optimal model (S1 Text).

## Results

### Suckling behaviour over weeks

**Fig 1:**
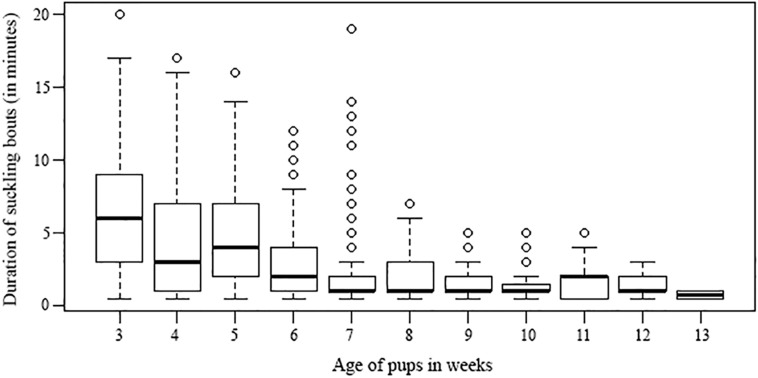
Suckling duration vs pup age. Box-whisker plot represents the decreasing pattern in the duration of suckling (irrespective of the initiator) over increasing pup age. Here suckling refers to the observed nursing or suckling bouts.

All mothers were observed to nurse their pups (1378 bouts in total), and in some cases allomothers were also observed to nurse non-filial pups (594 bouts), which was termed as allonursing (Please see the video clippings of nursing and allonursing bouts in S1 Video). At the 3^rd^ week, mothers invested 18 (± 9.28) % of their total time in nursing, thereby investing 41 (± 27.7) % of their total observed activity in this behaviour. This time decreased with pup age (Linear regression: R^2^ = 0.876, β = -0.936, P< 0.0001), until it stopped completely at the 13^th^ week. Pups at their 3^rd^ week of age spent 14.6 (± 9.14) % of their time in suckling from their mothers, which comprised of 16.5 (± 9.5) % of their total activity. Not only the time spent in suckling but also the duration of suckling bouts decreased as pups grew older (duration ~ age: t = -28.03, p < 0.0001, Model 1 in S1 Text, Fig 1). In the third week, suckling durations were 6.3 (± 4.2) % min, which reduced to 2.2 (± 2.9) % min by the 7^th^ week, and to 0.75 (± 0.3) % min by the 13^th^ week of pup age.

### Effects of pup age and litter size on suckling duration

**Fig 2:**
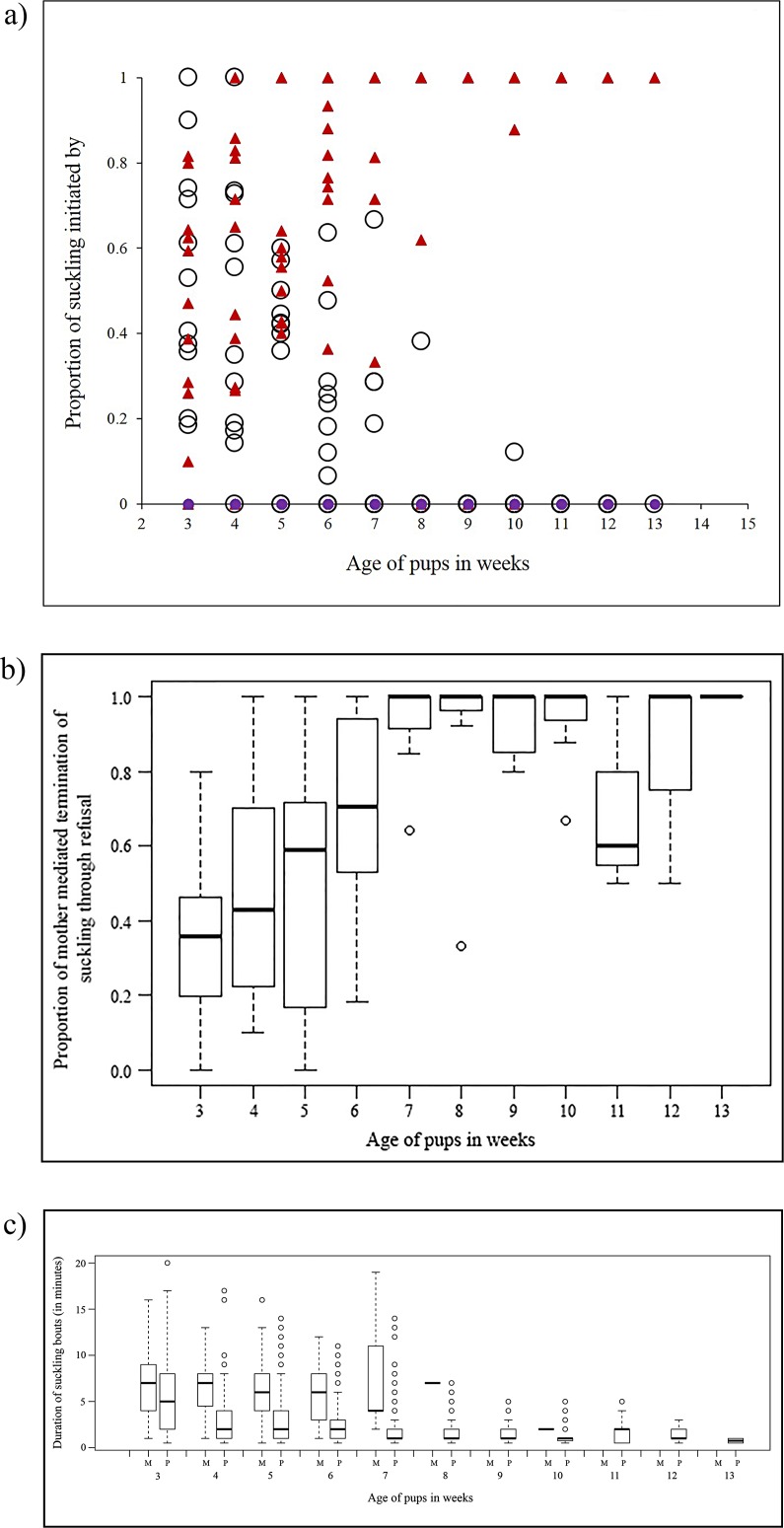
Initiation, termination and duration of suckling. (a) Scatter plot showing the proportion of total suckling initiated by the mother (empty circles), pups (triangles) and allomothers (solid circles) over pup age. (b) Box-whisker plot showing the increased trend of mother-mediated termination of suckling through refusal, over pup age. (c) Box- whisker plot showing the duration of mother and pup initiated suckling, over pup age (N = 11 litters).

Of the total number of suckling events in a week, the proportion of mother-initiated nursing (mo) and pup-initiated suckling (pup) changed with pup age (Fig 2A) and the current litter size (age: p < 0.0001; litter size: p = 0.00029. Model 2 in S1 Text). Mothers increasingly refused suckling solicitations (rf) as the pups grew older, and mother-mediated termination of suckling (through refusals) increased with pup age (age: p < 0.0001. Model 3 in S1 Text) (Fig 2B). The duration of mother-initiated nursing reduced drastically beyond the 7^th^ week (Fig 2C), when mother-mediated terminations reached 100% (Fig 2B), and the mothers stopped initiating nursing completely by the 11^th^ week (Fig 2A).

Suckling duration seems to be controlled by a combination of the identity of the initiator, pup age and the current litter size (Table 1, Model 4 in S1 Text). A three-way interaction in the GLMM suggests a significant role of both the pup age and the current litter size for mother and pup initiated suckling durations. As the pups grow older, the duration of mother initiated nursing bouts decreases in comparison to the pup initiated suckling bouts. However the overall duration of suckling bouts (irrespective of the initiator) decreases over pup ages and litter size. A two-way significant interaction suggests that the duration of mother and pup initiated suckling vary for different litter sizes (Table 1, Model 4 in S1 Text).

**Table 1:** Effects of the initiator, pup age and litter size on suckling duration.

**Fixed Effects**				
	**Estimate**	**Std. Error**	**t value**	**P value**
**Intercept**	10.20989	1.32828	7.687	**3.46e-14**
**Initiator**	-5.17190	1.34665	-3.841	**0.000126**
**Age**	-0.76597	0.27380	-2.798	**0.005189**
**LS**	-0.51550	0.30588	-1.685	0.092067
Initiator *Age	0.54558	0.28016	1.947	0.051599
Initiator *LS	0.85418	0.33336	2.562	**0.010456**
Age*LS	0.07249	0.06490	1.117	0.264121
Initiator *Age*LS	-0.17554	0.06731	-2.608	**0.009160**
**Random Effects**				
**Groups**	**Name**	**Variance**	**Std.Dev.**	
Group Identity	Intercept	1.156	1.075	
Residual		6.222	2.494	

Tabulated results of the generalized linear mixed effect model (GLMM) showing the effects of the initiator (Initiator), pup age (Age) and current litter size for a particular mother-litter group (LS) on the duration of suckling. Here suckling refers to the observed nursing or suckling bouts. P values for significant effects are shown in bold.

### Allonursing or milk theft?

**Fig 3:**
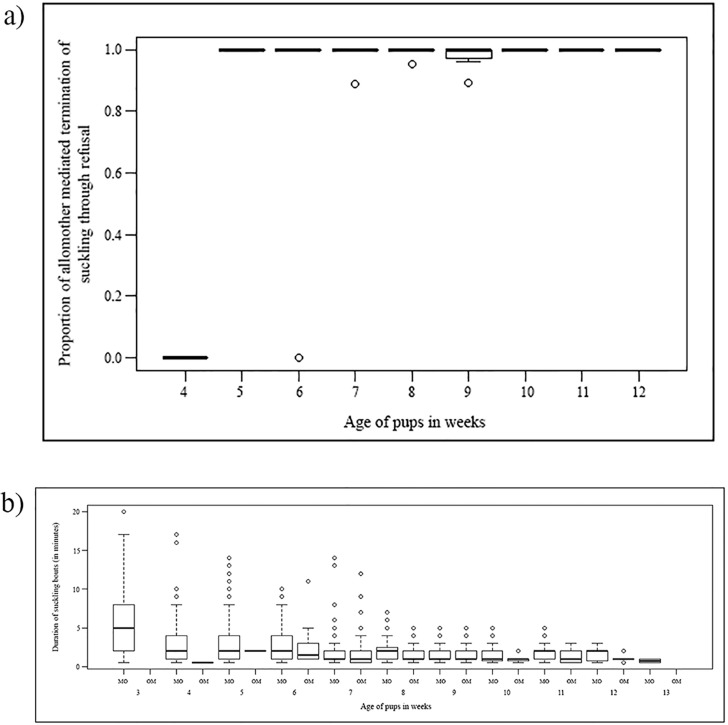
Allonursing or milk theft? (a) Box- whisker plot showing allomother-mediated termination of suckling through refusal over pup age. (b) Box- whisker plot showing the duration of suckling bouts where pups suckled from mothers and allomothers, over weeks.

In nine of the 11 mother-litter units, we observed high incidence of allonursing (594 bouts), and in eight of these nine units, the allonursing female was related to the pups. 100% of the allonursing bouts were initiated by pups, irrespective of their age, and the allomothers never volunteered to allonurse (Fig 2A). Most of the allonursing bouts were terminated by refusals from the allomothers, irrespective of the age of the pups (Fig 3A). The average suckling duration was 3.2 (± 2.3) min for the mothers whereas it was 1.5 (± 0.8) min for the allomothers. Thus the duration of suckling from mothers over the entire period of observations was significantly longer than that from allomothers (2 tailed t test; t = 5.158, df = 91.74, p < 0.0001; Fig 3B). However this difference was not consistent over the zone of conflict, from the 7^th^ to the 13^th^ week of pup age (2 tailed t test; t = 1.844, df = 33.73, p = 0.07). Thus while mothers nursed their pups voluntarily till the onset of weaning and on the whole showed a large degree of care, allomothers never volunteered to nurse non-filial pups but were victims of milk theft.

## Discussion

According to the predictions of POC theory, a mother is expected to invest in LRS, such that her chances of increasing fitness are high [5]. Kin selection theory suggests that individuals can increase their inclusive fitness benefits by investing in the survival of close kin [26]. Thus, while mothers are expected to show conflict towards their offspring over weaning in order to maximize direct fitness, they can benefit by additionally providing altruistic care to the offspring of related females to ensure indirect fitness benefits. The duration of time allocated to a behaviour is a good estimate of investment in the behaviour in terms of time activity budgets of individuals, and we considered the time spent in nursing as a measure of investment by the mothers and allomothers. Though the use of nursing duration as a measure of investment by mothers has been debated, it continues to be used as an indirect measurement of maternal investment [27,28]. Moreover, since we compared nursing/ suckling durations for the same set of individuals over time, this could be considered as a reliable marker for changing interest of the mothers and pups in suckling. Suckling duration reduced as the pups grew older, which could be due to the reduced interest of the mothers to nurse as well the increased efficiency of pups to extract milk. There is also evidence that the physiological needs of pups change rapidly in the first 3–4 weeks of age, determining their milk intake [29].

Our observations revealed that the proportion of mother-initiated nursing bouts reduces while the proportion of pup-initiated suckling bouts increases with pup age. However the mother-initiated nursing bouts were longer in the early weeks of the pups’ development as compared to the pup-initiated bouts. These results suggest the mother’s reduced interest in nursing as the pups grow older. Refusal of suckling by the mothers reaching 100% in the 7^th^ week of pup age and complete seizure of solicitations from the pups by the 13^th^ week is a good representation of the zone of conflict over weaning in the free-ranging dogs. Before the 7^th^ week the mother encourages suckling solicitations and also initiates nursing, and beyond the 13^th^ week, neither the mother nor the pups are interested in nursing/ suckling, leading to the resolution of conflict.

Very low levels of oxytocin administered to lactating females have been shown to be effective in eliciting the milk let down reflex [30], and suckling stimulation causes an immediate rise of oxytocin [31]. Hence, even the short suckling bouts during the later stages of development, and especially the allosuckling bouts could prove beneficial to pups. It has been suggested that allonursing can evolve when costs associated with the behaviour are low [32]. Allonursing is more likely to evolve in polytocous species, and in carnivorous cooperative breeders rather than omnivorous or herbivorous ones [32]. Dogs are promiscuous breeders with a scavenging lifestyle [17]. Multiple females often give birth to pups simultaneously, in close proximity of each other, increasing the chances of communal breeding (Paul and Bhadra, under review). Allonursing started as early as the 4^th^ week of pup age, all bouts being pup-initiated and allomother-terminated, and much shorter than maternal nursing bouts. Pups appear to adopt a snatch and run strategy while suckling from allomothers, as allomothers terminate the suckling whenever they identify free-riders and do not altruistically offer milk to non-filial pups. Thus pups benefit my using a selfish strategy of maximizing milk intake from any available lactating female. Hence allonursing in free-ranging dogs is an example of milk theft by pups, a behaviour more common in primates and ungulates [33] than in carnivores, and less likely in cooperative and communal breeders [32]. In a competitive and disturbed environment with limited resources and high mortality, this ability to snatch milk would be highly adaptive for the pups. Though apparently maladaptive for the mothers, this might indeed be an evolutionarily stable strategy if related females tend to den close to each other. Our observations provide support for this idea, as most of the observed allomothers were related to the pups. Coupled long term behavioural, hormonal and genetic studies in the future will help to understand whether milk theft by pups could lead to increased inclusive fitness benefits and LRS for the mothers.

## Supporting Information

S1 Table Details of the observed dog groups. The table represents the group identity of each observed mother-litter units along with their litter size at birth, year of data collection, location and habitat type of the observed units, etc. Presence or absence of allonursing has also been tabulated here.
(DOCX)

S1 Text Detailed description of the generalized linear mixed effect models (GLMM). Four models have been described in details along with the fixed and random effects that have been incorporated in the models. (doi:10.5061/dryad.d15b0).
(DOCX)

S1 Video Nursing and allonursing in free-ranging dogs in India. The video is comprised of video clippings that present the details of nursing and suckling behaviours in free-ranging dogs (*Canis lupus familiaris*) in India. It also represents the detail of suckling refusals and few examples of allonursing.
(MP4)
