# Supplementary material for: Selfish Pups: Weaning Conflict and Milk Theft in Free-Ranging Dogs
Source: PLoS One. 2017 Feb 8;12(2):e0170590. doi: 10.1371/journal.pone.0170590 (PMC5298236; doi:10.1371/journal.pone.0170590)
Supplement: S1 Table — The table represents the group identity of each observed mother-litter units along with their litter size at birth, year of data collection, location and habitat type of the observed units, etc. Presence or absence of allonursing has also been tabulated here. (DOCX) [file pone.0170590.s001.docx]

**Selfish pups: Weaning conflict and milk theft in free-ranging dogs**

**Manabi Paul^1^ and Anindita Bhadra^1,*^**

^1^Behaviour and Ecology Lab, Department of Biological Sciences, Indian Institute of Science Education and Research Kolkata, India

^*^Address for Correspondence:

Behaviour and Ecology Lab, Department of Biological Sciences,

# Indian Institute of Science Education and Research Kolkata

# Mohanpur Campus, Mohanpur,

# PIN 741246, West Bengal, INDIA

*tel.* 91-33-66340000-1223

*fax* **+**91-33-25873020

# *e-mail:* [abhadra@iiserkol.ac.in](mailto:ragh@ces.iisc.ernet.in)

| **Serial no.** | **Year** | **Group name** | **Mother-litter**  **units’ id** | **Litter size** | **Location** | **Habitat type** | **Latitude and Longitude** | **Nursing/**  **Suckling details noted** | **Allonursing present** |
| --- | --- | --- | --- | --- | --- | --- | --- | --- | --- |
| 1 | 2010-11 | CAN1 | CAN1 | 5 | IISER-K Campus | Suburban | 22.9638° N, 88.5246° E | No | No |
| 2 | 2010-11 | BUD | BUD1 | 4 | IISER-K Campus | Suburban | 22.9638° N, 88.5246° E | No | Yes |
| 3 | 2010-11 | LEL1 | LEL1 | 2 | IISER-K Campus | Suburban | 22.9638° N, 88.5246° E | No | No |
| 4 | 2010-11 | S1 | S1 | 2 | Saltlake, Kolkata | Urban | 22.5800° N, 88.4200° E | No | No |
| 5 | 2011-12 | BSF1 | RS4 | 5 | Kalyani | Suburban | 22.9750° N, 88.4344° E | No | No |
| 6 | 2011-12 | PLT1 | JCB | 2 | IISER-K Campus | Suburban | 22.9638° N, 88.5246° E | No | Yes |
| 7 | 2011-12 | PLT1 | MDB1 | 5 | IISER-K Campus | Suburban | 22.9638° N, 88.5246° E | No | No |
| 8 | 2011-12 | CAN2 | CAN2 | 5 | IISER-K Campus | Suburban | 22.9638° N, 88.5246° E | No | No |
| 9 | 2011-12 | GH | GH2 | 6 | IISER-K Campus | Suburban | 22.9638° N, 88.5246° E | No | No |
| 10 | 2011-12 | LEL2 | LEL2 | 2 | IISER-K Campus | Suburban | 22.9638° N, 88.5246° E | No | No |
| 11 | 2011-12 | S2 | S2 | 3 | Saltlake, Kolkata | Urban | 22.5800° N, 88.4200° E | No | No |
| 12 | 2013-14 | BSF2 | **RS1** | 2 | Kalyani | Suburban | 22.9750° N, 88.4344° E | **Yes** | Yes |
| 13 | 2013-14 | BSF2 | **RS2** | 4 | Kalyani | Suburban | 22.9750° N, 88.4344° E | **Yes** | Yes |
| 14 | 2013-14 | BSF2 | **RS3** | 2 | Kalyani | Suburban | 22.9750° N, 88.4344° E | **Yes** | Yes |
| 15 | 2013-14 | PF | **PF1** | 5 | IISER-K Campus | Suburban | 22.9638° N, 88.5246° E | **Yes** | Yes |
| 16 | 2013-14 | CAN3 | **CAN3** | 6 | IISER-K Campus | Suburban | 22.9638° N, 88.5246° E | **Yes** | No |
| 17 | 2013-14 | PLT2 | **MDB2** | 5 | IISER-K Campus | Suburban | 22.9638° N, 88.5246° E | **Yes** | No |
| 18 | 2014-15 | BSF3 | **BBR** | 4 | Kalyani | Suburban | 22.9750° N, 88.4344° E | **Yes** | Yes |
| 19 | 2014-15 | BSF3 | **KTI** | 2 | Kalyani | Suburban | 22.9750° N, 88.4344° E | **Yes** | Yes |
| 20 | 2014-15 | BSF3 | **WHI** | 2 | Kalyani | Suburban | 22.9750° N, 88.4344° E | **Yes** | Yes |
| 21 | 2014-15 | BSF3 | **BRN** | 2 | Kalyani | Suburban | 22.9750° N, 88.4344° E | **Yes** | Yes |
| 22 | 2014-15 | BSF3 | **RS5** | 3 | Kalyani | Suburban | 22.9750° N, 88.4344° E | **Yes** | Yes |
