# Supplementary material for: Selfish Pups: Weaning Conflict and Milk Theft in Free-Ranging Dogs
Source: PLoS One. 2017 Feb 8;12(2):e0170590. doi: 10.1371/journal.pone.0170590 (PMC5298236; doi:10.1371/journal.pone.0170590)
Supplement: S1 Text — Four models have been described in details along with the fixed and random effects that have been incorporated in the models. (doi:10.5061/dryad.d15b0). (DOCX) [file pone.0170590.s002.docx]

**Selfish pups: Weaning conflict and milk theft in free-ranging dogs**

**Manabi Paul^1^ and Anindita Bhadra^1,*^**

^1^Behaviour and Ecology Lab, Department of Biological Sciences, Indian Institute of Science Education and Research Kolkata, India

^*^Address for Correspondence:

Behaviour and Ecology Lab, Department of Biological Sciences,

# Indian Institute of Science Education and Research Kolkata

# Mohanpur Campus, Mohanpur,

# PIN 741246, West Bengal, INDIA

*tel.* 91-33-66340000-1223

*fax* **+**91-33-25873020

# *e-mail:* [abhadra@iiserkol.ac.in](mailto:ragh@ces.iisc.ernet.in)

**Model 1.**

We recorded the start and end time of each nursing/ suckling bouts observed along with the details of the

initiators. Nursing/ suckling bouts’ duration was calculated from the observed start and end time.

In order to check if the pup age had any impact on the duration of nursing/ suckling bouts, we ran a

Generalized linear mixed effect model (GLMM). We considered the duration of nursing/ suckling bouts as the response variable, pup age as the fixed effects and the mother-litter units’ identity as the random effects. A Gaussian distribution was considered for the response variable in the model.

**Variables used in the model:**

Response variable:

Duration of nursing/ suckling bouts (in minutes)- **dur**

Fixed effects:

Age of pups in weeks- **age**

Random effects:

Identity of mother-litter units- **fgr**

**Model: glmer (dur ~ +age + (1 | fgr), family = gaussian)**

**Results**

Random effects:

Groups Name Variance Std.Deffgr (Intercept) 1.101 1.049

Fixed effects:

Estimate Std. Error t value Pr(>|t|)

(Intercept) 7.58515 0.35466 21.39 1.94e-12 ***

**age -0.67677 0.02414 -28.03 < 2e-16** ***

Significant codes: 0 ‘***’ 0.001 ‘**’ 0.01 ‘*’ 0.05 ‘.’ 0.1 ‘ ’ 1

**Model 2.**

For generalized linear mixed-effects model (GLMM) analysis we categorized the initiators of each nursing/ suckling bouts into two categories i.e. initiated by the mother and initiated by the pup and used the Binomial family of distributions in R statistics. Hence nursing/ suckling was incorporated in the model as a binary variable, either initiated by the mother or the pup. We used the function “cbind” that created a matrix by binding the column vectors containing the numbers of “mother initiated nursing” and “pup initiated suckling” measured for each group per week. In this case R adds the two columns together to produce the correct binomial denominator.

The two parameters, i.e., the current litter size and age of the pups in weeks were considered as the fixed effects while the identity of the mother-litter units was taken as the random effect. We started with the full model, i.e., with all possible two-way interactions among the fixed effects. The two-way interaction was non-significant; we then reduced the model using standard protocol of backward selection method and ended up with the optimal model, the result of which is described below.

**Variables used in the model:**

Response variable:

Number of mother initiated nursing bouts (for each week) - **mo**

Number of pup initiated suckling bouts (for each week) - **pup**

Fixed effects:

Age of pups in weeks- **age**

Current litter size- **ls**

Random effects:

Identity of mother-litter units- **fgr**

**Model: glmer (cbind(mo, pup) ~ +age + ls + (1 | fgr), family = binomial)**

**Results**

Random effects:

Groups Name Variance Std.Dev.

fgr (Intercept) 0.5259 0.7252

Fixed effects:

Estimate Std. Error z value Pr(>|t|)

(Intercept) 0.7904 0.5881 1.344 0.17893

**age -0.6931 0.0414 -16.740 < 2e-16** ***

**ls 0.5173 0.1428 3.624 0.00029*****

Significant codes: 0 ‘***’ 0.001 ‘**’ 0.01 ‘*’ 0.05 ‘.’ 0.1 ‘ ’ 1

**Model 3.**

For each nursing/ suckling bouts observed, we recorded the time duration of bouts, the initiator (i.e. who initiated the nursing/ suckling bout) and the terminator (i.e. who terminated the nursing/ suckling bout) along with the behavior of the terminator to the pup who were suckled. Out of the total termination of the nursing/ suckling bouts observed, mother mediated termination was counted to calculate the proportion. We ran a GLMM considering the proportion of mother mediated termination of nursing/ suckling bouts as the response variable, pup age and the current litter size as fixed effects and the identity of

mother-litter units as the random effects.

We started with the full model, i.e., with all possible two-way interactions among the fixed effects. The two-way interaction was non-significant; we then reduced the model using standard protocol of backward selection method and ended up with the optimal model, the result of which is described below.

**Variables used in the model:**

Response variable:

Proportion of mother mediated termination of suckling bouts through refusal - **rfmo**

Fixed effects:

Age of pups in weeks- **age**

Current litter size- **ls**

Random effects:

Identity of mother-litter units- **fgr**

**Model: glmer (rfmo ~ +age + ls + (1 | fgr), family = gaussian)**

**Results**

Random effects:

Groups Name Variance Std.Dev.

fgr (Intercept) 0.01030 0.1015

Fixed effects:

Estimate Std. Error t value Pr(>|t|)

(Intercept) 0.10251 0.13779 0.744 0.467

**age 0.07669 0.01144 6.705 2.85e-09** ***

ls 0.03154 0.02951 1.069 0.306

Significant codes: 0 ‘***’ 0.001 ‘**’ 0.01 ‘*’ 0.05 ‘.’ 0.1 ‘ ’ 1

**Model 4.**

In order to check the effect of pup age, current litter size and the identity of the initiator (either the

mother or the pup) on the nursing/ suckling duration, we ran a GLMM considering the Gaussian distribution for the response variable i.e. the nursing/ suckling duration. Initiators’ identity, pup age and the

current litter size was incorporated in the model as the fixed effects whereas the identity of mother-litter units was considered as the random effect.

We started with the full model, i.e., with all possible two-way and three-way interactions among the

fixed effects. The three-way interaction was significant, hence we kept the model as the final one.

**Variables used in the model:**

Response variable:

Duration of suckling bouts in minutes: **dur**

Fixed effects:

Mother or pup initiated suckling: **Initiator**

Age of pups in weeks- **age**

Current litter size- **ls**

Random effects:

Identity of mother-litter units- **fgr**

**Model: glmer (dur ~ + Initiator * age * ls + (1 | fgr), family = gaussian)**

**Results**

Random effects:

Groups Name Variance Std.Dev.

fgr (Intercept) 1.156 1.075

Fixed effects:

Estimate Std. Error t value Pr(>|t|)

(Intercept) 10.20989 1.32828 7.687 3.46e-14***

**Initiator -5.17190 1.34665 -3.841 0.000126*****

**age -0.76597 0.27380 -2.798 0.005189****

ls -0.51550 0.30588 -1.685 0.092067**.**

Initiator*age 0.54558 0.28016 1.947 0.051599**.**

**Initiator*ls 0.85418 0.33336 2.562 0.010456***

age*ls 0.07249 0.06490 1.117 0.264121

**Initiator*age*ls -0.17554 0.06731 -2.608 0.009160****

Significant codes: 0 ‘***’ 0.001 ‘**’ 0.01 ‘*’ 0.05 ‘.’ 0.1 ‘ ’ 1

**Raw data for Model 1 and 4**

| Year | ID | Age | Current | Duration of bouts  (in minutes) | Initiator | Suckled by | |
| --- | --- | --- | --- | --- | --- | --- | --- |
|  |  |  | litter size |  |  | Mother | Allomother |
| 4 | CAN | 3 | 6 | 11 | MO | 1 |  |
| 4 | CAN | 3 | 6 | 8 | MO | 1 |  |
| 4 | CAN | 3 | 6 | 11 | MO | 1 |  |
| 4 | CAN | 3 | 6 | 8 | MO | 1 |  |
| 4 | CAN | 3 | 6 | 11 | MO | 1 |  |
| 4 | CAN | 3 | 6 | 11 | MO | 1 |  |
| 4 | CAN | 3 | 6 | 7 | MO | 1 |  |
| 4 | CAN | 3 | 6 | 7 | MO | 1 |  |
| 4 | CAN | 3 | 6 | 7 | MO | 1 |  |
| 4 | CAN | 3 | 6 | 7 | MO | 1 |  |
| 4 | CAN | 3 | 6 | 7 | MO | 1 |  |
| 4 | CAN | 3 | 6 | 7 | MO | 1 |  |
| 4 | CAN | 3 | 6 | 4 | MO | 1 |  |
| 4 | CAN | 3 | 6 | 8 | MO | 1 |  |
| 4 | CAN | 3 | 6 | 4 | MO | 1 |  |
| 4 | CAN | 3 | 6 | 4 | MO | 1 |  |
| 4 | CAN | 3 | 6 | 4 | MO | 1 |  |
| 4 | CAN | 3 | 6 | 4 | MO | 1 |  |
| 4 | CAN | 3 | 6 | 8 | Pup | 1 |  |
| 4 | CAN | 3 | 6 | 5 | Pup | 1 |  |
| 4 | MDB | 3 | 5 | 8 | MO | 1 |  |
| 4 | MDB | 3 | 5 | 8 | MO | 1 |  |
| 4 | MDB | 3 | 5 | 8 | MO | 1 |  |
| 4 | MDB | 3 | 5 | 8 | MO | 1 |  |
| 4 | MDB | 3 | 5 | 8 | MO | 1 |  |
| 4 | MDB | 3 | 5 | 7 | MO | 1 |  |
| 4 | MDB | 3 | 5 | 7 | MO | 1 |  |
| 4 | MDB | 3 | 5 | 7 | MO | 1 |  |
| 4 | MDB | 3 | 5 | 7 | MO | 1 |  |
| 4 | MDB | 3 | 5 | 7 | MO | 1 |  |
| 4 | MDB | 3 | 5 | 11 | MO | 1 |  |
| 4 | MDB | 3 | 5 | 11 | MO | 1 |  |
| 4 | MDB | 3 | 5 | 11 | MO | 1 |  |
| 4 | MDB | 3 | 5 | 11 | MO | 1 |  |
| 4 | MDB | 3 | 5 | 11 | MO | 1 |  |
| 4 | PF1 | 3 | 5 | 1 | MO | 1 |  |
| 4 | PF1 | 3 | 5 | 1 | MO | 1 |  |
| 4 | PF1 | 3 | 5 | 1 | MO | 1 |  |
| 4 | PF1 | 3 | 5 | 1 | MO | 1 |  |
| 4 | PF1 | 3 | 5 | 1 | MO | 1 |  |
| 4 | PF1 | 3 | 5 | 11 | Pup | 1 |  |
| 4 | PF1 | 3 | 5 | 12 | Pup | 1 |  |
| 4 | PF1 | 3 | 5 | 9 | Pup | 1 |  |
| 4 | PF1 | 3 | 5 | 9 | Pup | 1 |  |
| 4 | PF1 | 3 | 5 | 12 | Pup | 1 |  |
| 4 | PF1 | 3 | 5 | 1 | Pup | 1 |  |
| 4 | PF1 | 3 | 5 | 2 | MO | 1 |  |
| 4 | PF1 | 3 | 5 | 2 | MO | 1 |  |
| 4 | PF1 | 3 | 5 | 2 | MO | 1 |  |
| 4 | PF1 | 3 | 5 | 2 | MO | 1 |  |
| 4 | PF1 | 3 | 5 | 2 | MO | 1 |  |
| 4 | PF1 | 3 | 5 | 12 | MO | 1 |  |
| 4 | PF1 | 3 | 5 | 12 | MO | 1 |  |
| 4 | PF1 | 3 | 5 | 12 | MO | 1 |  |
| 4 | PF1 | 3 | 5 | 8 | MO | 1 |  |
| 4 | PF1 | 3 | 5 | 12 | MO | 1 |  |
| 4 | PF1 | 3 | 5 | 16 | MO | 1 |  |
| 4 | PF1 | 3 | 5 | 11 | MO | 1 |  |
| 4 | PF1 | 3 | 5 | 15 | MO | 1 |  |
| 4 | PF1 | 3 | 5 | 11 | MO | 1 |  |
| 4 | PF1 | 3 | 5 | 16 | MO | 1 |  |
| 4 | PF1 | 3 | 5 | 6 | Pup | 1 |  |
| 4 | PF1 | 3 | 5 | 2 | Pup | 1 |  |
| 4 | PF1 | 3 | 5 | 2 | Pup | 1 |  |
| 4 | PF1 | 3 | 5 | 2 | Pup | 1 |  |
| 4 | PF1 | 3 | 5 | 2 | Pup | 1 |  |
| 4 | PF1 | 3 | 5 | 2 | Pup | 1 |  |
| 4 | PF1 | 3 | 5 | 3 | MO | 1 |  |
| 4 | PF1 | 3 | 5 | 3 | MO | 1 |  |
| 4 | PF1 | 3 | 5 | 3 | MO | 1 |  |
| 4 | PF1 | 3 | 5 | 3 | MO | 1 |  |
| 4 | PF1 | 3 | 5 | 3 | MO | 1 |  |
| 4 | PF1 | 3 | 5 | 8 | MO | 1 |  |
| 4 | PF1 | 3 | 5 | 10 | MO | 1 |  |
| 4 | PF1 | 3 | 5 | 7 | MO | 1 |  |
| 4 | PF1 | 3 | 5 | 8 | MO | 1 |  |
| 4 | PF1 | 3 | 5 | 7 | MO | 1 |  |
| 4 | RS1 | 3 | 2 | 4 | MO | 1 |  |
| 4 | RS1 | 3 | 2 | 4 | MO | 1 |  |
| 4 | RS1 | 3 | 2 | 7 | MO | 1 |  |
| 4 | RS1 | 3 | 2 | 7 | MO | 1 |  |
| 4 | RS1 | 3 | 2 | 1 | Pup | 1 |  |
| 4 | RS1 | 3 | 2 | 3 | MO | 1 |  |
| 4 | RS1 | 3 | 2 | 2 | MO | 1 |  |
| 4 | RS1 | 3 | 2 | 4 | Pup | 1 |  |
| 4 | RS1 | 3 | 2 | 11 | Pup | 1 |  |
| 4 | RS1 | 3 | 2 | 10 | Pup | 1 |  |
| 4 | RS1 | 3 | 2 | 8 | Pup | 1 |  |
| 4 | RS1 | 3 | 2 | 8 | Pup | 1 |  |
| 4 | RS1 | 3 | 2 | 4 | Pup | 1 |  |
| 4 | RS1 | 3 | 2 | 1 | Pup | 1 |  |
| 4 | RS1 | 3 | 2 | 2 | Pup | 1 |  |
| 4 | RS1 | 3 | 2 | 9 | Pup | 1 |  |
| 4 | RS1 | 3 | 2 | 9 | Pup | 1 |  |
| 4 | RS1 | 3 | 2 | 10 | MO | 1 |  |
| 4 | RS1 | 3 | 2 | 7 | MO | 1 |  |
| 4 | RS1 | 3 | 2 | 1 | Pup | 1 |  |
| 4 | RS1 | 3 | 2 | 5 | Pup | 1 |  |
| 4 | RS1 | 3 | 2 | 1 | Pup | 1 |  |
| 4 | RS1 | 3 | 2 | 11 | Pup | 1 |  |
| 4 | RS1 | 3 | 2 | 10 | Pup | 1 |  |
| 4 | RS1 | 3 | 2 | 6 | MO | 1 |  |
| 4 | RS1 | 3 | 2 | 6 | MO | 1 |  |
| 4 | RS1 | 3 | 2 | 3 | Pup | 1 |  |
| 4 | RS1 | 3 | 2 | 3 | Pup | 1 |  |
| 4 | RS2 | 3 | 4 | 15 | MO | 1 |  |
| 4 | RS2 | 3 | 4 | 15 | MO | 1 |  |
| 4 | RS2 | 3 | 4 | 15 | MO | 1 |  |
| 4 | RS2 | 3 | 4 | 15 | MO | 1 |  |
| 4 | RS2 | 3 | 4 | 4 | MO | 1 |  |
| 4 | RS2 | 3 | 4 | 4 | MO | 1 |  |
| 4 | RS2 | 3 | 4 | 4 | MO | 1 |  |
| 4 | RS2 | 3 | 4 | 4 | MO | 1 |  |
| 4 | RS2 | 3 | 4 | 1 | MO | 1 |  |
| 4 | RS2 | 3 | 4 | 1 | MO | 1 |  |
| 4 | RS2 | 3 | 4 | 1 | MO | 1 |  |
| 4 | RS2 | 3 | 4 | 1 | MO | 1 |  |
| 4 | RS2 | 3 | 4 | 1 | MO | 1 |  |
| 4 | RS2 | 3 | 4 | 1 | MO | 1 |  |
| 4 | RS2 | 3 | 4 | 1 | MO | 1 |  |
| 4 | RS2 | 3 | 4 | 1 | MO | 1 |  |
| 4 | RS2 | 3 | 4 | 7 | Pup | 1 |  |
| 4 | RS2 | 3 | 4 | 7 | Pup | 1 |  |
| 4 | RS2 | 3 | 4 | 7 | Pup | 1 |  |
| 4 | RS2 | 3 | 4 | 7 | Pup | 1 |  |
| 4 | RS2 | 3 | 4 | 2 | MO | 1 |  |
| 4 | RS2 | 3 | 4 | 2 | MO | 1 |  |
| 4 | RS2 | 3 | 4 | 2 | MO | 1 |  |
| 4 | RS2 | 3 | 4 | 2 | MO | 1 |  |
| 4 | RS2 | 3 | 4 | 10 | Pup | 1 |  |
| 4 | RS2 | 3 | 4 | 10 | Pup | 1 |  |
| 4 | RS2 | 3 | 4 | 10 | Pup | 1 |  |
| 4 | RS2 | 3 | 4 | 10 | Pup | 1 |  |
| 4 | RS2 | 3 | 4 | 20 | Pup | 1 |  |
| 4 | RS2 | 3 | 4 | 20 | Pup | 1 |  |
| 4 | RS2 | 3 | 4 | 20 | Pup | 1 |  |
| 4 | RS2 | 3 | 4 | 20 | Pup | 1 |  |
| 4 | RS2 | 3 | 4 | 15 | MO | 1 |  |
| 4 | RS2 | 3 | 4 | 15 | MO | 1 |  |
| 4 | RS2 | 3 | 4 | 15 | MO | 1 |  |
| 4 | RS2 | 3 | 4 | 15 | MO | 1 |  |
| 4 | RS2 | 3 | 4 | 9 | MO | 1 |  |
| 4 | RS2 | 3 | 4 | 9 | MO | 1 |  |
| 4 | RS2 | 3 | 4 | 9 | MO | 1 |  |
| 4 | RS2 | 3 | 4 | 9 | MO | 1 |  |
| 4 | RS2 | 3 | 4 | 11 | MO | 1 |  |
| 4 | RS2 | 3 | 4 | 11 | MO | 1 |  |
| 4 | RS2 | 3 | 4 | 11 | MO | 1 |  |
| 4 | RS2 | 3 | 4 | 11 | MO | 1 |  |
| 4 | RS2 | 3 | 4 | 6 | MO | 1 |  |
| 4 | RS2 | 3 | 4 | 6 | MO | 1 |  |
| 4 | RS2 | 3 | 4 | 6 | MO | 1 |  |
| 4 | RS2 | 3 | 4 | 6 | MO | 1 |  |
| 4 | RS2 | 3 | 4 | 7 | Pup | 1 |  |
| 4 | RS2 | 3 | 4 | 7 | Pup | 1 |  |
| 4 | RS3 | 3 | 2 | 8 | MO | 1 |  |
| 4 | RS3 | 3 | 2 | 14 | Pup | 1 |  |
| 4 | RS3 | 3 | 2 | 6 | Pup | 1 |  |
| 4 | RS3 | 3 | 2 | 2 | Pup | 1 |  |
| 4 | RS3 | 3 | 2 | 2 | Pup | 1 |  |
| 4 | RS3 | 3 | 2 | 3 | Pup | 1 |  |
| 4 | RS3 | 3 | 2 | 3 | Pup | 1 |  |
| 4 | RS3 | 3 | 2 | 14 | MO | 1 |  |
| 4 | RS3 | 3 | 2 | 14 | MO | 1 |  |
| 4 | RS3 | 3 | 2 | 17 | Pup | 1 |  |
| 4 | RS3 | 3 | 2 | 16 | Pup | 1 |  |
| 4 | RS3 | 3 | 2 | 10 | Pup | 1 |  |
| 4 | RS3 | 3 | 2 | 7 | Pup | 1 |  |
| 4 | RS3 | 3 | 2 | 8 | Pup | 1 |  |
| 4 | RS3 | 3 | 2 | 8 | Pup | 1 |  |
| 4 | RS3 | 3 | 2 | 4 | Pup | 1 |  |
| 4 | RS3 | 3 | 2 | 4 | Pup | 1 |  |
| 4 | RS3 | 3 | 2 | 9 | MO | 1 |  |
| 4 | RS3 | 3 | 2 | 9 | MO | 1 |  |
| 4 | RS3 | 3 | 2 | 3 | Pup | 1 |  |
| 4 | RS3 | 3 | 2 | 3 | Pup | 1 |  |
| 4 | RS3 | 3 | 2 | 14 | Pup | 1 |  |
| 4 | RS3 | 3 | 2 | 8 | Pup | 1 |  |
| 4 | RS3 | 3 | 2 | 5 | Pup | 1 |  |
| 4 | RS3 | 3 | 2 | 7 | Pup | 1 |  |
| 5 | BRN | 3 | 2 | 6 | MO | 1 |  |
| 5 | BRN | 3 | 2 | 5 | Pup | 1 |  |
| 5 | BRN | 3 | 2 | 7 | Pup | 1 |  |
| 5 | BRN | 3 | 2 | 1 | Pup | 1 |  |
| 5 | BRN | 3 | 2 | 7 | Pup | 1 |  |
| 5 | BRN | 3 | 2 | 0.5 | Pup | 1 |  |
| 5 | BRN | 3 | 2 | 2 | Pup | 1 |  |
| 5 | BRN | 3 | 2 | 0.5 | Pup | 1 |  |
| 5 | BRN | 3 | 2 | 0.5 | Pup | 1 |  |
| 5 | BRN | 3 | 2 | 1 | Pup | 1 |  |
| 5 | BRN | 3 | 2 | 0.5 | Pup | 1 |  |
| 5 | BRN | 3 | 2 | 0.5 | Pup | 1 |  |
| 5 | BRN | 3 | 2 | 1 | Pup | 1 |  |
| 5 | BRN | 3 | 2 | 3 | Pup | 1 |  |
| 5 | BRN | 3 | 2 | 2 | Pup | 1 |  |
| 5 | BRN | 3 | 2 | 5 | Pup | 1 |  |
| 5 | BRN | 3 | 2 | 9 | Pup | 1 |  |
| 5 | BRN | 3 | 2 | 1 | Pup | 1 |  |
| 5 | BRN | 3 | 2 | 1 | Pup | 1 |  |
| 5 | BRN | 3 | 2 | 7 | Pup | 1 |  |
| 5 | BRN | 3 | 2 | 5 | Pup | 1 |  |
| 5 | BRN | 3 | 2 | 6 | MO | 1 |  |
| 5 | BRN | 3 | 2 | 10 | MO | 1 |  |
| 5 | BRN | 3 | 2 | 7 | MO | 1 |  |
| 5 | BRN | 3 | 2 | 7 | MO | 1 |  |
| 5 | BRN | 3 | 2 | 8 | Pup | 1 |  |
| 5 | BRN | 3 | 2 | 8 | Pup | 1 |  |
| 5 | KTI | 3 | 2 | 10 | MO | 1 |  |
| 5 | KTI | 3 | 2 | 10 | MO | 1 |  |
| 5 | KTI | 3 | 2 | 8 | MO | 1 |  |
| 5 | KTI | 3 | 2 | 5 | MO | 1 |  |
| 5 | KTI | 3 | 2 | 1 | Pup | 1 |  |
| 5 | KTI | 3 | 2 | 9 | Pup | 1 |  |
| 5 | KTI | 3 | 2 | 5 | Pup | 1 |  |
| 5 | KTI | 3 | 2 | 7 | Pup | 1 |  |
| 5 | KTI | 3 | 2 | 7 | Pup | 1 |  |
| 5 | KTI | 3 | 2 | 5 | Pup | 1 |  |
| 5 | KTI | 3 | 2 | 4 | Pup | 1 |  |
| 5 | KTI | 3 | 2 | 4 | MO | 1 |  |
| 5 | KTI | 3 | 2 | 4 | MO | 1 |  |
| 5 | KTI | 3 | 2 | 2 | Pup | 1 |  |
| 5 | KTI | 3 | 2 | 9 | Pup | 1 |  |
| 5 | KTI | 3 | 2 | 9 | Pup | 1 |  |
| 5 | BBR | 3 | 4 | 6 | MO | 1 |  |
| 5 | BBR | 3 | 4 | 6 | MO | 1 |  |
| 5 | BBR | 3 | 4 | 4 | Pup | 1 |  |
| 5 | BBR | 3 | 4 | 8 | Pup | 1 |  |
| 5 | BBR | 3 | 4 | 12 | Pup | 1 |  |
| 5 | BBR | 3 | 4 | 14 | Pup | 1 |  |
| 5 | BBR | 3 | 4 | 8 | Pup | 1 |  |
| 5 | BBR | 3 | 4 | 0.5 | Pup | 1 |  |
| 5 | BBR | 3 | 4 | 6 | MO | 1 |  |
| 5 | BBR | 3 | 4 | 6 | MO | 1 |  |
| 5 | BBR | 3 | 4 | 3 | MO | 1 |  |
| 5 | BBR | 3 | 4 | 2 | MO | 1 |  |
| 5 | BBR | 3 | 4 | 4 | Pup | 1 |  |
| 5 | BBR | 3 | 4 | 4 | Pup | 1 |  |
| 5 | BBR | 3 | 4 | 7 | Pup | 1 |  |
| 5 | BBR | 3 | 4 | 8 | Pup | 1 |  |
| 5 | BBR | 3 | 4 | 3 | MO | 1 |  |
| 5 | BBR | 3 | 4 | 9 | MO | 1 |  |
| 5 | BBR | 3 | 4 | 10 | MO | 1 |  |
| 5 | BBR | 3 | 4 | 8 | MO | 1 |  |
| 5 | BBR | 3 | 4 | 9 | Pup | 1 |  |
| 5 | BBR | 3 | 4 | 3 | Pup | 1 |  |
| 5 | BBR | 3 | 4 | 2 | Pup | 1 |  |
| 5 | BBR | 3 | 4 | 4 | Pup | 1 |  |
| 5 | BBR | 3 | 4 | 3 | Pup | 1 |  |
| 5 | BBR | 3 | 4 | 5 | MO | 1 |  |
| 5 | BBR | 3 | 4 | 5 | MO | 1 |  |
| 5 | BBR | 3 | 4 | 5 | MO | 1 |  |
| 5 | BBR | 3 | 4 | 5 | MO | 1 |  |
| 5 | BBR | 3 | 4 | 10 | MO | 1 |  |
| 5 | BBR | 3 | 4 | 8 | MO | 1 |  |
| 5 | BBR | 3 | 4 | 7 | MO | 1 |  |
| 5 | BBR | 3 | 4 | 10 | MO | 1 |  |
| 5 | BBR | 3 | 4 | 8 | MO | 1 |  |
| 5 | BBR | 3 | 4 | 5 | MO | 1 |  |
| 5 | BBR | 3 | 4 | 6 | MO | 1 |  |
| 5 | BBR | 3 | 4 | 8 | MO | 1 |  |
| 5 | BBR | 3 | 4 | 1 | Pup | 1 |  |
| 5 | BBR | 3 | 4 | 0.5 | Pup | 1 |  |
| 5 | BBR | 3 | 4 | 0.5 | Pup | 1 |  |
| 5 | BBR | 3 | 4 | 1 | Pup | 1 |  |
| 5 | BBR | 3 | 4 | 8 | MO | 1 |  |
| 5 | BBR | 3 | 4 | 8 | MO | 1 |  |
| 5 | BBR | 3 | 4 | 8 | MO | 1 |  |
| 5 | BBR | 3 | 4 | 8 | MO | 1 |  |
| 5 | BBR | 3 | 4 | 2 | MO | 1 |  |
| 5 | BBR | 3 | 4 | 2 | MO | 1 |  |
| 5 | BBR | 3 | 4 | 2 | MO | 1 |  |
| 5 | BBR | 3 | 4 | 2 | MO | 1 |  |
| 5 | WHI | 3 | 2 | 6 | MO | 1 |  |
| 5 | WHI | 3 | 2 | 9 | Pup | 1 |  |
| 5 | WHI | 3 | 2 | 5 | Pup | 1 |  |
| 5 | WHI | 3 | 2 | 6 | MO | 1 |  |
| 5 | WHI | 3 | 2 | 6 | MO | 1 |  |
| 5 | WHI | 3 | 2 | 9 | MO | 1 |  |
| 5 | WHI | 3 | 2 | 9 | MO | 1 |  |
| 5 | WHI | 3 | 2 | 2 | Pup | 1 |  |
| 5 | WHI | 3 | 2 | 9 | Pup | 1 |  |
| 5 | WHI | 3 | 2 | 8 | Pup | 1 |  |
| 5 | WHI | 3 | 2 | 2 | MO | 1 |  |
| 5 | WHI | 3 | 2 | 2 | MO | 1 |  |
| 5 | WHI | 3 | 2 | 4 | Pup | 1 |  |
| 5 | WHI | 3 | 2 | 3 | Pup | 1 |  |
| 5 | WHI | 3 | 2 | 1 | Pup | 1 |  |
| 5 | WHI | 3 | 2 | 7 | MO | 1 |  |
| 5 | WHI | 3 | 2 | 8 | MO | 1 |  |
| 5 | RS5 | 3 | 3 | 7 | MO | 1 |  |
| 5 | RS5 | 3 | 3 | 4 | MO | 1 |  |
| 5 | RS5 | 3 | 3 | 10 | MO | 1 |  |
| 5 | RS5 | 3 | 3 | 5 | Pup | 1 |  |
| 5 | RS5 | 3 | 3 | 6 | MO | 1 |  |
| 5 | RS5 | 3 | 3 | 6 | MO | 1 |  |
| 5 | RS5 | 3 | 3 | 6 | MO | 1 |  |
| 5 | RS5 | 3 | 3 | 2 | Pup | 1 |  |
| 5 | RS5 | 3 | 3 | 1 | Pup | 1 |  |
| 5 | RS5 | 3 | 3 | 2 | Pup | 1 |  |
| 5 | RS5 | 3 | 3 | 8 | MO | 1 |  |
| 5 | RS5 | 3 | 3 | 7 | MO | 1 |  |
| 5 | RS5 | 3 | 3 | 6 | MO | 1 |  |
| 5 | RS5 | 3 | 3 | 4 | MO | 1 |  |
| 5 | RS5 | 3 | 3 | 4 | MO | 1 |  |
| 5 | RS5 | 3 | 3 | 2 | MO | 1 |  |
| 5 | RS5 | 3 | 3 | 3 | Pup | 1 |  |
| 5 | RS5 | 3 | 3 | 1 | Pup | 1 |  |
| 5 | RS5 | 3 | 3 | 1 | Pup | 1 |  |
| 5 | RS5 | 3 | 3 | 1 | Pup | 1 |  |
| 5 | RS5 | 3 | 3 | 3 | Pup | 1 |  |
| 5 | RS5 | 3 | 3 | 16 | Pup | 1 |  |
| 5 | RS5 | 3 | 3 | 2 | Pup | 1 |  |
| 5 | RS5 | 3 | 3 | 6 | Pup | 1 |  |
| 5 | RS5 | 3 | 3 | 6 | Pup | 1 |  |
| 5 | RS5 | 3 | 3 | 0.5 | Pup | 1 |  |
| 5 | RS5 | 3 | 3 | 1 | Pup | 1 |  |
| 5 | RS5 | 3 | 3 | 0.5 | Pup | 1 |  |
| 5 | RS5 | 3 | 3 | 10 | Pup | 1 |  |
| 5 | RS5 | 3 | 3 | 3 | Pup | 1 |  |
| 5 | RS5 | 3 | 3 | 2 | Pup | 1 |  |
| 5 | RS5 | 3 | 3 | 4 | MO | 1 |  |
| 5 | RS5 | 3 | 3 | 5 | MO | 1 |  |
| 5 | RS5 | 3 | 3 | 5 | MO | 1 |  |
| 5 | RS5 | 3 | 3 | 3 | Pup | 1 |  |
| 5 | RS5 | 3 | 3 | 2 | Pup | 1 |  |
| 5 | RS5 | 3 | 3 | 2 | Pup | 1 |  |
| 4 | CAN | 4 | 6 | 8 | MO | 1 |  |
| 4 | CAN | 4 | 6 | 8 | MO | 1 |  |
| 4 | CAN | 4 | 6 | 8 | MO | 1 |  |
| 4 | CAN | 4 | 6 | 8 | MO | 1 |  |
| 4 | CAN | 4 | 6 | 8 | MO | 1 |  |
| 4 | CAN | 4 | 6 | 8 | MO | 1 |  |
| 4 | CAN | 4 | 6 | 8 | MO | 1 |  |
| 4 | CAN | 4 | 6 | 8 | MO | 1 |  |
| 4 | CAN | 4 | 6 | 8 | MO | 1 |  |
| 4 | CAN | 4 | 6 | 8 | MO | 1 |  |
| 4 | CAN | 4 | 6 | 8 | MO | 1 |  |
| 4 | CAN | 4 | 6 | 8 | MO | 1 |  |
| 4 | CAN | 4 | 6 | 9 | MO | 1 |  |
| 4 | CAN | 4 | 6 | 9 | MO | 1 |  |
| 4 | CAN | 4 | 6 | 9 | MO | 1 |  |
| 4 | CAN | 4 | 6 | 9 | MO | 1 |  |
| 4 | CAN | 4 | 6 | 9 | MO | 1 |  |
| 4 | CAN | 4 | 6 | 9 | MO | 1 |  |
| 4 | MDB | 4 | 5 | 1 | MO | 1 |  |
| 4 | MDB | 4 | 5 | 1 | MO | 1 |  |
| 4 | MDB | 4 | 5 | 1 | MO | 1 |  |
| 4 | MDB | 4 | 5 | 1 | MO | 1 |  |
| 4 | MDB | 4 | 5 | 1 | MO | 1 |  |
| 4 | MDB | 4 | 5 | 6 | MO | 1 |  |
| 4 | MDB | 4 | 5 | 6 | MO | 1 |  |
| 4 | MDB | 4 | 5 | 6 | MO | 1 |  |
| 4 | MDB | 4 | 5 | 6 | MO | 1 |  |
| 4 | MDB | 4 | 5 | 6 | MO | 1 |  |
| 4 | MDB | 4 | 5 | 8 | Pup | 1 |  |
| 4 | MDB | 4 | 5 | 6 | Pup | 1 |  |
| 4 | MDB | 4 | 5 | 9 | MO | 1 |  |
| 4 | MDB | 4 | 5 | 9 | MO | 1 |  |
| 4 | MDB | 4 | 5 | 9 | MO | 1 |  |
| 4 | MDB | 4 | 5 | 9 | MO | 1 |  |
| 4 | MDB | 4 | 5 | 9 | MO | 1 |  |
| 4 | MDB | 4 | 5 | 4 | MO | 1 |  |
| 4 | MDB | 4 | 5 | 4 | MO | 1 |  |
| 4 | MDB | 4 | 5 | 4 | MO | 1 |  |
| 4 | MDB | 4 | 5 | 4 | MO | 1 |  |
| 4 | MDB | 4 | 5 | 4 | MO | 1 |  |
| 4 | MDB | 4 | 5 | 2 | MO | 1 |  |
| 4 | MDB | 4 | 5 | 2 | MO | 1 |  |
| 4 | MDB | 4 | 5 | 1 | Pup | 1 |  |
| 4 | MDB | 4 | 5 | 1 | Pup | 1 |  |
| 4 | MDB | 4 | 5 | 1 | Pup | 1 |  |
| 4 | MDB | 4 | 5 | 2 | Pup | 1 |  |
| 4 | MDB | 4 | 5 | 2 | Pup | 1 |  |
| 4 | MDB | 4 | 5 | 4 | Pup | 1 |  |
| 4 | PF1 | 4 | 5 | 2 | Pup | 1 |  |
| 4 | PF1 | 4 | 5 | 2 | Pup | 1 |  |
| 4 | PF1 | 4 | 5 | 2 | Pup | 1 |  |
| 4 | PF1 | 4 | 5 | 2 | Pup | 1 |  |
| 4 | PF1 | 4 | 5 | 2 | Pup | 1 |  |
| 4 | PF1 | 4 | 5 | 1 | Pup | 1 |  |
| 4 | PF1 | 4 | 5 | 1 | Pup | 1 |  |
| 4 | PF1 | 4 | 5 | 1 | Pup | 1 |  |
| 4 | PF1 | 4 | 5 | 3 | Pup | 1 |  |
| 4 | PF1 | 4 | 5 | 3 | Pup | 1 |  |
| 4 | PF1 | 4 | 5 | 0.5 | Pup |  | 1 |
| 4 | PF1 | 4 | 5 | 0.5 | Pup |  | 1 |
| 4 | PF1 | 4 | 5 | 2 | Pup | 1 |  |
| 4 | PF1 | 4 | 5 | 2 | Pup | 1 |  |
| 4 | PF1 | 4 | 5 | 2 | Pup | 1 |  |
| 4 | PF1 | 4 | 5 | 3 | Pup | 1 |  |
| 4 | PF1 | 4 | 5 | 3 | Pup | 1 |  |
| 4 | PF1 | 4 | 5 | 3 | Pup | 1 |  |
| 4 | PF1 | 4 | 5 | 3 | Pup | 1 |  |
| 4 | PF1 | 4 | 5 | 3 | Pup | 1 |  |
| 4 | PF1 | 4 | 5 | 3 | Pup | 1 |  |
| 4 | PF1 | 4 | 5 | 3 | Pup | 1 |  |
| 4 | PF1 | 4 | 5 | 6 | Pup | 1 |  |
| 4 | PF1 | 4 | 5 | 6 | Pup | 1 |  |
| 4 | PF1 | 4 | 5 | 6 | Pup | 1 |  |
| 4 | PF1 | 4 | 5 | 6 | Pup | 1 |  |
| 4 | PF1 | 4 | 5 | 6 | Pup | 1 |  |
| 4 | PF1 | 4 | 5 | 0.5 | Pup | 1 |  |
| 4 | PF1 | 4 | 5 | 1 | Pup | 1 |  |
| 4 | PF1 | 4 | 5 | 1 | Pup | 1 |  |
| 4 | PF1 | 4 | 5 | 3 | Pup | 1 |  |
| 4 | PF1 | 4 | 5 | 3 | Pup | 1 |  |
| 4 | PF1 | 4 | 5 | 3 | Pup | 1 |  |
| 4 | PF1 | 4 | 5 | 2 | Pup | 1 |  |
| 4 | PF1 | 4 | 5 | 2 | Pup | 1 |  |
| 4 | PF1 | 4 | 5 | 2 | Pup | 1 |  |
| 4 | PF1 | 4 | 5 | 3 | Pup | 1 |  |
| 4 | PF1 | 4 | 5 | 3 | Pup | 1 |  |
| 4 | PF1 | 4 | 5 | 3 | Pup | 1 |  |
| 4 | RS1 | 4 | 2 | 8 | MO | 1 |  |
| 4 | RS1 | 4 | 2 | 8 | MO | 1 |  |
| 4 | RS1 | 4 | 2 | 1 | Pup | 1 |  |
| 4 | RS1 | 4 | 2 | 7 | MO | 1 |  |
| 4 | RS1 | 4 | 2 | 7 | MO | 1 |  |
| 4 | RS1 | 4 | 2 | 1 | Pup | 1 |  |
| 4 | RS1 | 4 | 2 | 4 | MO | 1 |  |
| 4 | RS1 | 4 | 2 | 3 | MO | 1 |  |
| 4 | RS1 | 4 | 2 | 9 | MO | 1 |  |
| 4 | RS1 | 4 | 2 | 7 | MO | 1 |  |
| 4 | RS1 | 4 | 2 | 3 | Pup | 1 |  |
| 4 | RS1 | 4 | 2 | 2 | Pup | 1 |  |
| 4 | RS1 | 4 | 2 | 3 | MO | 1 |  |
| 4 | RS1 | 4 | 2 | 3 | MO | 1 |  |
| 4 | RS1 | 4 | 2 | 7 | Pup | 1 |  |
| 4 | RS1 | 4 | 2 | 7 | Pup | 1 |  |
| 4 | RS1 | 4 | 2 | 7 | Pup | 1 |  |
| 4 | RS1 | 4 | 2 | 8 | Pup | 1 |  |
| 4 | RS2 | 4 | 4 | 8 | MO | 1 |  |
| 4 | RS2 | 4 | 4 | 8 | MO | 1 |  |
| 4 | RS2 | 4 | 4 | 8 | MO | 1 |  |
| 4 | RS2 | 4 | 4 | 8 | MO | 1 |  |
| 4 | RS2 | 4 | 4 | 9 | Pup | 1 |  |
| 4 | RS2 | 4 | 4 | 9 | Pup | 1 |  |
| 4 | RS2 | 4 | 4 | 9 | Pup | 1 |  |
| 4 | RS2 | 4 | 4 | 9 | Pup | 1 |  |
| 4 | RS2 | 4 | 4 | 9 | Pup | 1 |  |
| 4 | RS2 | 4 | 4 | 9 | Pup | 1 |  |
| 4 | RS2 | 4 | 4 | 1 | Pup | 1 |  |
| 4 | RS2 | 4 | 4 | 1 | Pup | 1 |  |
| 4 | RS2 | 4 | 4 | 1 | Pup | 1 |  |
| 4 | RS2 | 4 | 4 | 1 | Pup | 1 |  |
| 4 | RS2 | 4 | 4 | 1 | Pup | 1 |  |
| 4 | RS2 | 4 | 4 | 1 | Pup | 1 |  |
| 4 | RS2 | 4 | 4 | 9 | Pup | 1 |  |
| 4 | RS2 | 4 | 4 | 9 | Pup | 1 |  |
| 4 | RS2 | 4 | 4 | 9 | Pup | 1 |  |
| 4 | RS2 | 4 | 4 | 9 | Pup | 1 |  |
| 4 | RS2 | 4 | 4 | 9 | Pup | 1 |  |
| 4 | RS2 | 4 | 4 | 9 | Pup | 1 |  |
| 4 | RS2 | 4 | 4 | 2 | Pup | 1 |  |
| 4 | RS2 | 4 | 4 | 2 | Pup | 1 |  |
| 4 | RS2 | 4 | 4 | 2 | Pup | 1 |  |
| 4 | RS2 | 4 | 4 | 2 | Pup | 1 |  |
| 4 | RS2 | 4 | 4 | 7 | Pup | 1 |  |
| 4 | RS2 | 4 | 4 | 7 | Pup | 1 |  |
| 4 | RS2 | 4 | 4 | 1 | Pup | 1 |  |
| 4 | RS2 | 4 | 4 | 1 | Pup | 1 |  |
| 4 | RS2 | 4 | 4 | 1 | Pup | 1 |  |
| 4 | RS2 | 4 | 4 | 1 | Pup | 1 |  |
| 4 | RS2 | 4 | 4 | 1 | Pup | 1 |  |
| 4 | RS2 | 4 | 4 | 1 | Pup | 1 |  |
| 4 | RS2 | 4 | 4 | 1 | Pup | 1 |  |
| 4 | RS2 | 4 | 4 | 1 | Pup | 1 |  |
| 4 | RS2 | 4 | 4 | 1 | Pup | 1 |  |
| 4 | RS2 | 4 | 4 | 1 | Pup | 1 |  |
| 4 | RS2 | 4 | 4 | 0.5 | Pup | 1 |  |
| 4 | RS2 | 4 | 4 | 0.5 | Pup | 1 |  |
| 4 | RS2 | 4 | 4 | 1 | Pup | 1 |  |
| 4 | RS2 | 4 | 4 | 1 | Pup | 1 |  |
| 4 | RS2 | 4 | 4 | 2 | Pup | 1 |  |
| 4 | RS2 | 4 | 4 | 2 | Pup | 1 |  |
| 4 | RS2 | 4 | 4 | 2 | Pup | 1 |  |
| 4 | RS2 | 4 | 4 | 1 | Pup | 1 |  |
| 4 | RS2 | 4 | 4 | 1 | Pup | 1 |  |
| 4 | RS2 | 4 | 4 | 1 | Pup | 1 |  |
| 4 | RS2 | 4 | 4 | 0.5 | Pup | 1 |  |
| 4 | RS2 | 4 | 4 | 0.5 | Pup | 1 |  |
| 4 | RS2 | 4 | 4 | 0.5 | Pup | 1 |  |
| 4 | RS2 | 4 | 4 | 0.5 | Pup | 1 |  |
| 4 | RS2 | 4 | 4 | 0.5 | Pup | 1 |  |
| 4 | RS2 | 4 | 4 | 0.5 | Pup | 1 |  |
| 4 | RS2 | 4 | 4 | 2 | Pup | 1 |  |
| 4 | RS2 | 4 | 4 | 2 | Pup | 1 |  |
| 4 | RS2 | 4 | 4 | 0.5 | Pup | 1 |  |
| 4 | RS2 | 4 | 4 | 0.5 | Pup | 1 |  |
| 4 | RS2 | 4 | 4 | 0.5 | Pup | 1 |  |
| 4 | RS2 | 4 | 4 | 1 | Pup | 1 |  |
| 4 | RS2 | 4 | 4 | 5 | Pup | 1 |  |
| 4 | RS2 | 4 | 4 | 1 | Pup | 1 |  |
| 4 | RS2 | 4 | 4 | 8 | MO | 1 |  |
| 4 | RS2 | 4 | 4 | 8 | MO | 1 |  |
| 4 | RS2 | 4 | 4 | 8 | MO | 1 |  |
| 4 | RS2 | 4 | 4 | 8 | MO | 1 |  |
| 4 | RS2 | 4 | 4 | 6 | MO | 1 |  |
| 4 | RS2 | 4 | 4 | 6 | MO | 1 |  |
| 4 | RS2 | 4 | 4 | 6 | MO | 1 |  |
| 4 | RS2 | 4 | 4 | 6 | MO | 1 |  |
| 4 | RS3 | 4 | 2 | 6 | Pup | 1 |  |
| 4 | RS3 | 4 | 2 | 2 | Pup | 1 |  |
| 4 | RS3 | 4 | 2 | 9 | Pup | 1 |  |
| 4 | RS3 | 4 | 2 | 5 | MO | 1 |  |
| 4 | RS3 | 4 | 2 | 5 | MO | 1 |  |
| 4 | RS3 | 4 | 2 | 7 | Pup | 1 |  |
| 4 | RS3 | 4 | 2 | 4 | Pup | 1 |  |
| 4 | RS3 | 4 | 2 | 1 | MO | 1 |  |
| 4 | RS3 | 4 | 2 | 1 | MO | 1 |  |
| 4 | RS3 | 4 | 2 | 16 | Pup | 1 |  |
| 4 | RS3 | 4 | 2 | 17 | Pup | 1 |  |
| 4 | RS3 | 4 | 2 | 2 | Pup | 1 |  |
| 4 | RS3 | 4 | 2 | 1 | Pup | 1 |  |
| 4 | RS3 | 4 | 2 | 1 | Pup | 1 |  |
| 5 | BRN | 4 | 2 | 5 | MO | 1 |  |
| 5 | BRN | 4 | 2 | 5 | MO | 1 |  |
| 5 | BRN | 4 | 2 | 3 | Pup | 1 |  |
| 5 | BRN | 4 | 2 | 2 | Pup | 1 |  |
| 5 | BRN | 4 | 2 | 2 | Pup | 1 |  |
| 5 | BRN | 4 | 2 | 2 | Pup | 1 |  |
| 5 | BRN | 4 | 2 | 4 | Pup | 1 |  |
| 5 | BRN | 4 | 2 | 3 | Pup | 1 |  |
| 5 | BRN | 4 | 2 | 2 | Pup | 1 |  |
| 5 | BRN | 4 | 2 | 1 | Pup | 1 |  |
| 5 | BRN | 4 | 2 | 0.5 | Pup | 1 |  |
| 5 | BRN | 4 | 2 | 1 | Pup | 1 |  |
| 5 | BRN | 4 | 2 | 1 | Pup | 1 |  |
| 5 | BRN | 4 | 2 | 0.5 | Pup | 1 |  |
| 5 | KTI | 4 | 2 | 4 | MO | 1 |  |
| 5 | KTI | 4 | 2 | 4 | MO | 1 |  |
| 5 | KTI | 4 | 2 | 6 | MO | 1 |  |
| 5 | KTI | 4 | 2 | 7 | MO | 1 |  |
| 5 | KTI | 4 | 2 | 7 | MO | 1 |  |
| 5 | KTI | 4 | 2 | 7 | MO | 1 |  |
| 5 | KTI | 4 | 2 | 7 | MO | 1 |  |
| 5 | KTI | 4 | 2 | 8 | MO | 1 |  |
| 5 | KTI | 4 | 2 | 3 | Pup | 1 |  |
| 5 | KTI | 4 | 2 | 2 | Pup | 1 |  |
| 5 | KTI | 4 | 2 | 1 | Pup | 1 |  |
| 5 | KTI | 4 | 2 | 2 | Pup | 1 |  |
| 5 | KTI | 4 | 2 | 2 | Pup | 1 |  |
| 5 | KTI | 4 | 2 | 2 | Pup | 1 |  |
| 5 | KTI | 4 | 2 | 1 | Pup | 1 |  |
| 5 | KTI | 4 | 2 | 2 | Pup | 1 |  |
| 5 | KTI | 4 | 2 | 6 | Pup | 1 |  |
| 5 | KTI | 4 | 2 | 5 | Pup | 1 |  |
| 5 | KTI | 4 | 2 | 6 | Pup | 1 |  |
| 5 | KTI | 4 | 2 | 7 | Pup | 1 |  |
| 5 | KTI | 4 | 2 | 6 | Pup | 1 |  |
| 5 | KTI | 4 | 2 | 2 | Pup | 1 |  |
| 5 | KTI | 4 | 2 | 8 | Pup | 1 |  |
| 5 | KTI | 4 | 2 | 8 | Pup | 1 |  |
| 5 | KTI | 4 | 2 | 5 | MO | 1 |  |
| 5 | KTI | 4 | 2 | 5 | MO | 1 |  |
| 5 | KTI | 4 | 2 | 3 | Pup | 1 |  |
| 5 | KTI | 4 | 2 | 2 | Pup | 1 |  |
| 5 | KTI | 4 | 2 | 1 | Pup | 1 |  |
| 5 | KTI | 4 | 2 | 3 | Pup | 1 |  |
| 5 | KTI | 4 | 2 | 3 | Pup | 1 |  |
| 5 | KTI | 4 | 2 | 5 | MO | 1 |  |
| 5 | KTI | 4 | 2 | 5 | MO | 1 |  |
| 5 | KTI | 4 | 2 | 2 | Pup | 1 |  |
| 5 | KTI | 4 | 2 | 9 | Pup | 1 |  |
| 5 | KTI | 4 | 2 | 8 | Pup | 1 |  |
| 5 | KTI | 4 | 2 | 4 | MO | 1 |  |
| 5 | KTI | 4 | 2 | 4 | MO | 1 |  |
| 5 | KTI | 4 | 2 | 8 | Pup | 1 |  |
| 5 | KTI | 4 | 2 | 8 | Pup | 1 |  |
| 5 | BBR | 4 | 4 | 6 | MO | 1 |  |
| 5 | BBR | 4 | 4 | 6 | MO | 1 |  |
| 5 | BBR | 4 | 4 | 6 | MO | 1 |  |
| 5 | BBR | 4 | 4 | 6 | MO | 1 |  |
| 5 | BBR | 4 | 4 | 4 | MO | 1 |  |
| 5 | BBR | 4 | 4 | 6 | MO | 1 |  |
| 5 | BBR | 4 | 4 | 6 | MO | 1 |  |
| 5 | BBR | 4 | 4 | 6 | MO | 1 |  |
| 5 | BBR | 4 | 4 | 3 | Pup | 1 |  |
| 5 | BBR | 4 | 4 | 4 | Pup | 1 |  |
| 5 | BBR | 4 | 4 | 3 | Pup | 1 |  |
| 5 | BBR | 4 | 4 | 1 | Pup | 1 |  |
| 5 | BBR | 4 | 4 | 4 | MO | 1 |  |
| 5 | BBR | 4 | 4 | 4 | MO | 1 |  |
| 5 | BBR | 4 | 4 | 4 | MO | 1 |  |
| 5 | BBR | 4 | 4 | 4 | MO | 1 |  |
| 5 | BBR | 4 | 4 | 3 | MO | 1 |  |
| 5 | BBR | 4 | 4 | 3 | MO | 1 |  |
| 5 | BBR | 4 | 4 | 3 | MO | 1 |  |
| 5 | BBR | 4 | 4 | 3 | MO | 1 |  |
| 5 | BBR | 4 | 4 | 4 | Pup | 1 |  |
| 5 | BBR | 4 | 4 | 1 | Pup | 1 |  |
| 5 | BBR | 4 | 4 | 3 | Pup | 1 |  |
| 5 | BBR | 4 | 4 | 0.5 | Pup | 1 |  |
| 5 | BBR | 4 | 4 | 0.5 | Pup | 1 |  |
| 5 | BBR | 4 | 4 | 7 | MO | 1 |  |
| 5 | BBR | 4 | 4 | 7 | MO | 1 |  |
| 5 | BBR | 4 | 4 | 7 | MO | 1 |  |
| 5 | BBR | 4 | 4 | 7 | MO | 1 |  |
| 5 | BBR | 4 | 4 | 7 | MO | 1 |  |
| 5 | BBR | 4 | 4 | 7 | MO | 1 |  |
| 5 | BBR | 4 | 4 | 7 | MO | 1 |  |
| 5 | BBR | 4 | 4 | 7 | MO | 1 |  |
| 5 | WHI | 4 | 2 | 5 | MO | 1 |  |
| 5 | WHI | 4 | 2 | 10 | MO | 1 |  |
| 5 | WHI | 4 | 2 | 4 | Pup | 1 |  |
| 5 | WHI | 4 | 2 | 1 | Pup | 1 |  |
| 5 | WHI | 4 | 2 | 4 | Pup | 1 |  |
| 5 | WHI | 4 | 2 | 1 | Pup | 1 |  |
| 5 | WHI | 4 | 2 | 0.5 | Pup | 1 |  |
| 5 | WHI | 4 | 2 | 4 | MO | 1 |  |
| 5 | WHI | 4 | 2 | 6 | MO | 1 |  |
| 5 | WHI | 4 | 2 | 4 | Pup | 1 |  |
| 5 | WHI | 4 | 2 | 3 | Pup | 1 |  |
| 5 | WHI | 4 | 2 | 2 | Pup | 1 |  |
| 5 | WHI | 4 | 2 | 2 | Pup | 1 |  |
| 5 | WHI | 4 | 2 | 7 | MO | 1 |  |
| 5 | WHI | 4 | 2 | 8 | MO | 1 |  |
| 5 | WHI | 4 | 2 | 1 | Pup | 1 |  |
| 5 | WHI | 4 | 2 | 0.5 | Pup | 1 |  |
| 5 | WHI | 4 | 2 | 4 | Pup | 1 |  |
| 5 | WHI | 4 | 2 | 7 | Pup | 1 |  |
| 5 | WHI | 4 | 2 | 6 | MO | 1 |  |
| 5 | WHI | 4 | 2 | 7 | MO | 1 |  |
| 5 | WHI | 4 | 2 | 7 | MO | 1 |  |
| 5 | WHI | 4 | 2 | 8 | MO | 1 |  |
| 5 | WHI | 4 | 2 | 1 | Pup | 1 |  |
| 5 | WHI | 4 | 2 | 1 | Pup | 1 |  |
| 5 | WHI | 4 | 2 | 1 | Pup | 1 |  |
| 5 | WHI | 4 | 2 | 1 | Pup | 1 |  |
| 5 | WHI | 4 | 2 | 2 | Pup | 1 |  |
| 5 | WHI | 4 | 2 | 0.5 | Pup | 1 |  |
| 5 | WHI | 4 | 2 | 0.5 | Pup | 1 |  |
| 5 | WHI | 4 | 2 | 1 | Pup | 1 |  |
| 5 | WHI | 4 | 2 | 2 | Pup | 1 |  |
| 5 | WHI | 4 | 2 | 0.5 | Pup | 1 |  |
| 5 | WHI | 4 | 2 | 2 | Pup | 1 |  |
| 5 | WHI | 4 | 2 | 3 | Pup | 1 |  |
| 5 | WHI | 4 | 2 | 0.5 | Pup | 1 |  |
| 5 | WHI | 4 | 2 | 0.5 | Pup | 1 |  |
| 5 | WHI | 4 | 2 | 0.5 | Pup | 1 |  |
| 5 | WHI | 4 | 2 | 3 | Pup | 1 |  |
| 5 | WHI | 4 | 2 | 3 | Pup | 1 |  |
| 5 | WHI | 4 | 2 | 1 | Pup | 1 |  |
| 5 | WHI | 4 | 2 | 0.5 | Pup | 1 |  |
| 5 | WHI | 4 | 2 | 0.5 | Pup | 1 |  |
| 5 | WHI | 4 | 2 | 1 | Pup | 1 |  |
| 5 | WHI | 4 | 2 | 1 | Pup | 1 |  |
| 5 | WHI | 4 | 2 | 1 | Pup | 1 |  |
| 5 | WHI | 4 | 2 | 1 | Pup | 1 |  |
| 5 | WHI | 4 | 2 | 1 | Pup | 1 |  |
| 5 | WHI | 4 | 2 | 3 | Pup | 1 |  |
| 5 | WHI | 4 | 2 | 7 | Pup | 1 |  |
| 5 | WHI | 4 | 2 | 3 | Pup | 1 |  |
| 5 | WHI | 4 | 2 | 4 | Pup | 1 |  |
| 5 | WHI | 4 | 2 | 2 | Pup | 1 |  |
| 5 | RS5 | 4 | 3 | 12 | MO | 1 |  |
| 5 | RS5 | 4 | 3 | 9 | MO | 1 |  |
| 5 | RS5 | 4 | 3 | 13 | MO | 1 |  |
| 5 | RS5 | 4 | 3 | 10 | MO | 1 |  |
| 5 | RS5 | 4 | 3 | 9 | MO | 1 |  |
| 5 | RS5 | 4 | 3 | 11 | MO | 1 |  |
| 5 | RS5 | 4 | 3 | 2 | Pup | 1 |  |
| 5 | RS5 | 4 | 3 | 7 | MO | 1 |  |
| 5 | RS5 | 4 | 3 | 8 | MO | 1 |  |
| 5 | RS5 | 4 | 3 | 8 | Pup | 1 |  |
| 5 | RS5 | 4 | 3 | 2 | Pup | 1 |  |
| 5 | RS5 | 4 | 3 | 2 | Pup | 1 |  |
| 5 | RS5 | 4 | 3 | 10 | Pup | 1 |  |
| 5 | RS5 | 4 | 3 | 9 | Pup | 1 |  |
| 5 | RS5 | 4 | 3 | 10 | Pup | 1 |  |
| 5 | RS5 | 4 | 3 | 8 | MO | 1 |  |
| 5 | RS5 | 4 | 3 | 8 | MO | 1 |  |
| 5 | RS5 | 4 | 3 | 8 | MO | 1 |  |
| 4 | CAN | 5 | 6 | 7 | MO | 1 |  |
| 4 | CAN | 5 | 6 | 7 | MO | 1 |  |
| 4 | CAN | 5 | 6 | 7 | MO | 1 |  |
| 4 | CAN | 5 | 6 | 7 | MO | 1 |  |
| 4 | CAN | 5 | 6 | 7 | MO | 1 |  |
| 4 | CAN | 5 | 6 | 7 | MO | 1 |  |
| 4 | CAN | 5 | 6 | 9 | MO | 1 |  |
| 4 | CAN | 5 | 6 | 9 | MO | 1 |  |
| 4 | CAN | 5 | 6 | 9 | MO | 1 |  |
| 4 | CAN | 5 | 6 | 9 | MO | 1 |  |
| 4 | CAN | 5 | 6 | 6 | MO | 1 |  |
| 4 | CAN | 5 | 6 | 9 | MO | 1 |  |
| 4 | CAN | 5 | 6 | 14 | Pup | 1 |  |
| 4 | CAN | 5 | 6 | 14 | Pup | 1 |  |
| 4 | CAN | 5 | 6 | 12 | Pup | 1 |  |
| 4 | CAN | 5 | 6 | 12 | Pup | 1 |  |
| 4 | CAN | 5 | 6 | 12 | Pup | 1 |  |
| 4 | CAN | 5 | 6 | 12 | Pup | 1 |  |
| 4 | CAN | 5 | 6 | 8 | MO | 1 |  |
| 4 | CAN | 5 | 6 | 8 | MO | 1 |  |
| 4 | CAN | 5 | 6 | 7 | MO | 1 |  |
| 4 | CAN | 5 | 6 | 7 | MO | 1 |  |
| 4 | CAN | 5 | 6 | 6 | MO | 1 |  |
| 4 | CAN | 5 | 6 | 6 | MO | 1 |  |
| 4 | CAN | 5 | 6 | 2 | Pup | 1 |  |
| 4 | CAN | 5 | 6 | 2 | Pup | 1 |  |
| 4 | CAN | 5 | 6 | 2 | Pup | 1 |  |
| 4 | CAN | 5 | 6 | 2 | Pup | 1 |  |
| 4 | CAN | 5 | 6 | 2 | Pup | 1 |  |
| 4 | CAN | 5 | 6 | 2 | Pup | 1 |  |
| 4 | CAN | 5 | 6 | 2 | Pup | 1 |  |
| 4 | CAN | 5 | 6 | 1 | Pup | 1 |  |
| 4 | CAN | 5 | 6 | 8 | MO | 1 |  |
| 4 | CAN | 5 | 6 | 4 | MO | 1 |  |
| 4 | CAN | 5 | 6 | 10 | MO | 1 |  |
| 4 | CAN | 5 | 6 | 9 | MO | 1 |  |
| 4 | CAN | 5 | 6 | 9 | MO | 1 |  |
| 4 | CAN | 5 | 6 | 8 | MO | 1 |  |
| 4 | CAN | 5 | 6 | 3 | Pup | 1 |  |
| 4 | CAN | 5 | 6 | 2 | Pup | 1 |  |
| 4 | PF1 | 5 | 4 | 1 | Pup | 1 |  |
| 4 | PF1 | 5 | 4 | 1 | Pup | 1 |  |
| 4 | PF1 | 5 | 4 | 1 | Pup | 1 |  |
| 4 | PF1 | 5 | 4 | 1 | Pup | 1 |  |
| 4 | PF1 | 5 | 4 | 1 | Pup | 1 |  |
| 4 | PF1 | 5 | 4 | 3 | Pup | 1 |  |
| 4 | PF1 | 5 | 4 | 3 | Pup | 1 |  |
| 4 | PF1 | 5 | 4 | 3 | Pup | 1 |  |
| 4 | PF1 | 5 | 4 | 2 | Pup | 1 |  |
| 4 | PF1 | 5 | 4 | 2 | Pup | 1 |  |
| 4 | PF1 | 5 | 4 | 2 | Pup | 1 |  |
| 4 | PF1 | 5 | 4 | 2 | Pup | 1 |  |
| 4 | PF1 | 5 | 4 | 0.5 | Pup | 1 |  |
| 4 | PF1 | 5 | 4 | 0.5 | Pup | 1 |  |
| 4 | PF1 | 5 | 4 | 0.5 | Pup | 1 |  |
| 4 | PF1 | 5 | 4 | 0.5 | Pup | 1 |  |
| 4 | PF1 | 5 | 4 | 2 | Pup | 1 |  |
| 4 | PF1 | 5 | 4 | 2 | Pup | 1 |  |
| 4 | PF1 | 5 | 4 | 2 | Pup | 1 |  |
| 4 | PF1 | 5 | 4 | 2 | Pup | 1 |  |
| 4 | PF1 | 5 | 4 | 0.5 | Pup | 1 |  |
| 4 | PF1 | 5 | 4 | 1 | Pup | 1 |  |
| 4 | PF1 | 5 | 4 | 1 | Pup | 1 |  |
| 4 | PF1 | 5 | 4 | 1 | Pup | 1 |  |
| 4 | PF1 | 5 | 4 | 1 | Pup | 1 |  |
| 4 | PF1 | 5 | 4 | 1 | Pup | 1 |  |
| 4 | PF1 | 5 | 4 | 3 | Pup | 1 |  |
| 4 | PF1 | 5 | 4 | 3 | Pup | 1 |  |
| 4 | PF1 | 5 | 4 | 0.5 | Pup | 1 |  |
| 4 | RS1 | 5 | 2 | 4 | Pup | 1 |  |
| 4 | RS1 | 5 | 2 | 4 | Pup | 1 |  |
| 4 | RS1 | 5 | 2 | 8 | MO | 1 |  |
| 4 | RS1 | 5 | 2 | 6 | MO | 1 |  |
| 4 | RS1 | 5 | 2 | 2 | Pup | 1 |  |
| 4 | RS2 | 5 | 4 | 4 | MO | 1 |  |
| 4 | RS2 | 5 | 4 | 4 | MO | 1 |  |
| 4 | RS2 | 5 | 4 | 4 | MO | 1 |  |
| 4 | RS2 | 5 | 4 | 4 | MO | 1 |  |
| 4 | RS2 | 5 | 4 | 3 | Pup | 1 |  |
| 4 | RS2 | 5 | 4 | 1 | Pup | 1 |  |
| 4 | RS2 | 5 | 4 | 6 | MO | 1 |  |
| 4 | RS2 | 5 | 4 | 8 | MO | 1 |  |
| 4 | RS2 | 5 | 4 | 5 | MO | 1 |  |
| 4 | RS2 | 5 | 4 | 5 | MO | 1 |  |
| 4 | RS2 | 5 | 4 | 3 | Pup | 1 |  |
| 4 | RS2 | 5 | 4 | 3 | Pup | 1 |  |
| 4 | RS2 | 5 | 4 | 1 | Pup | 1 |  |
| 4 | RS2 | 5 | 4 | 1 | Pup | 1 |  |
| 4 | RS2 | 5 | 4 | 1 | Pup | 1 |  |
| 4 | RS2 | 5 | 4 | 1 | Pup | 1 |  |
| 4 | RS2 | 5 | 4 | 3 | MO | 1 |  |
| 4 | RS2 | 5 | 4 | 3 | MO | 1 |  |
| 4 | RS2 | 5 | 4 | 3 | MO | 1 |  |
| 4 | RS2 | 5 | 4 | 3 | MO | 1 |  |
| 4 | RS2 | 5 | 4 | 1 | Pup | 1 |  |
| 4 | RS2 | 5 | 4 | 1 | Pup | 1 |  |
| 4 | RS2 | 5 | 4 | 1 | Pup | 1 |  |
| 4 | RS2 | 5 | 4 | 1 | Pup | 1 |  |
| 4 | RS2 | 5 | 4 | 3 | Pup | 1 |  |
| 4 | RS2 | 5 | 4 | 6 | MO | 1 |  |
| 4 | RS2 | 5 | 4 | 6 | MO | 1 |  |
| 4 | RS2 | 5 | 4 | 6 | MO | 1 |  |
| 4 | RS2 | 5 | 4 | 6 | MO | 1 |  |
| 4 | RS2 | 5 | 4 | 0.5 | Pup | 1 |  |
| 4 | RS2 | 5 | 4 | 2 | Pup | 1 |  |
| 4 | RS2 | 5 | 4 | 2 | Pup | 1 |  |
| 4 | RS2 | 5 | 4 | 0.5 | Pup | 1 |  |
| 4 | RS2 | 5 | 4 | 0.5 | Pup | 1 |  |
| 4 | RS2 | 5 | 4 | 1 | Pup | 1 |  |
| 4 | RS2 | 5 | 4 | 1 | Pup | 1 |  |
| 4 | RS2 | 5 | 4 | 4 | MO | 1 |  |
| 4 | RS2 | 5 | 4 | 4 | MO | 1 |  |
| 4 | RS2 | 5 | 4 | 4 | MO | 1 |  |
| 4 | RS2 | 5 | 4 | 4 | MO | 1 |  |
| 4 | RS2 | 5 | 4 | 3 | MO | 1 |  |
| 4 | RS2 | 5 | 4 | 3 | MO | 1 |  |
| 4 | RS2 | 5 | 4 | 3 | MO | 1 |  |
| 4 | RS2 | 5 | 4 | 3 | MO | 1 |  |
| 4 | RS2 | 5 | 4 | 2 | Pup | 1 |  |
| 4 | RS2 | 5 | 4 | 1 | Pup | 1 |  |
| 4 | RS2 | 5 | 4 | 1 | Pup | 1 |  |
| 4 | RS2 | 5 | 4 | 1 | Pup | 1 |  |
| 4 | RS2 | 5 | 4 | 10 | MO | 1 |  |
| 4 | RS2 | 5 | 4 | 10 | MO | 1 |  |
| 4 | RS2 | 5 | 4 | 10 | MO | 1 |  |
| 4 | RS2 | 5 | 4 | 10 | MO | 1 |  |
| 4 | RS2 | 5 | 4 | 2 | Pup | 1 |  |
| 4 | RS2 | 5 | 4 | 1 | Pup | 1 |  |
| 4 | RS2 | 5 | 4 | 0.5 | Pup | 1 |  |
| 4 | RS2 | 5 | 4 | 1 | Pup | 1 |  |
| 4 | RS3 | 5 | 2 | 6 | Pup | 1 |  |
| 4 | RS3 | 5 | 2 | 16 | MO | 1 |  |
| 4 | RS3 | 5 | 2 | 12 | MO | 1 |  |
| 4 | RS3 | 5 | 2 | 1 | Pup | 1 |  |
| 4 | RS3 | 5 | 2 | 7 | MO | 1 |  |
| 4 | RS3 | 5 | 2 | 9 | MO | 1 |  |
| 4 | RS3 | 5 | 2 | 4 | Pup | 1 |  |
| 4 | RS3 | 5 | 2 | 4 | Pup | 1 |  |
| 4 | RS3 | 5 | 2 | 2 | Pup | 1 |  |
| 4 | RS3 | 5 | 2 | 2 | Pup | 1 |  |
| 4 | RS3 | 5 | 2 | 11 | Pup | 1 |  |
| 4 | RS3 | 5 | 2 | 13 | Pup | 1 |  |
| 4 | RS3 | 5 | 2 | 3 | Pup | 1 |  |
| 4 | RS3 | 5 | 2 | 3 | MO | 1 |  |
| 4 | RS3 | 5 | 2 | 3 | MO | 1 |  |
| 4 | RS3 | 5 | 2 | 2 | Pup | 1 |  |
| 4 | RS3 | 5 | 2 | 2 | Pup | 1 |  |
| 4 | RS3 | 5 | 2 | 3 | MO | 1 |  |
| 4 | RS3 | 5 | 2 | 3 | MO | 1 |  |
| 5 | BRN | 5 | 2 | 2 | Pup |  | 1 |
| 5 | BRN | 5 | 2 | 1 | Pup | 1 |  |
| 5 | BRN | 5 | 2 | 6 | Pup | 1 |  |
| 5 | BRN | 5 | 2 | 4 | Pup | 1 |  |
| 5 | BRN | 5 | 2 | 4 | Pup | 1 |  |
| 5 | BRN | 5 | 2 | 5 | Pup | 1 |  |
| 5 | BRN | 5 | 2 | 5 | Pup | 1 |  |
| 5 | BRN | 5 | 2 | 2 | Pup | 1 |  |
| 5 | BRN | 5 | 2 | 0.5 | Pup | 1 |  |
| 5 | BRN | 5 | 2 | 4 | Pup | 1 |  |
| 5 | BRN | 5 | 2 | 2 | Pup | 1 |  |
| 5 | BRN | 5 | 2 | 2 | Pup | 1 |  |
| 5 | KTI | 5 | 2 | 13 | MO | 1 |  |
| 5 | KTI | 5 | 2 | 8 | MO | 1 |  |
| 5 | KTI | 5 | 2 | 1 | Pup | 1 |  |
| 5 | KTI | 5 | 2 | 1 | Pup | 1 |  |
| 5 | KTI | 5 | 2 | 5 | MO | 1 |  |
| 5 | KTI | 5 | 2 | 7 | MO | 1 |  |
| 5 | KTI | 5 | 2 | 1 | Pup | 1 |  |
| 5 | KTI | 5 | 2 | 2 | Pup | 1 |  |
| 5 | KTI | 5 | 2 | 1 | Pup | 1 |  |
| 5 | KTI | 5 | 2 | 4 | Pup | 1 |  |
| 5 | KTI | 5 | 2 | 6 | Pup | 1 |  |
| 5 | KTI | 5 | 2 | 2 | Pup | 1 |  |
| 5 | KTI | 5 | 2 | 2 | Pup | 1 |  |
| 5 | KTI | 5 | 2 | 5 | MO | 1 |  |
| 5 | KTI | 5 | 2 | 5 | MO | 1 |  |
| 5 | KTI | 5 | 2 | 7 | Pup | 1 |  |
| 5 | KTI | 5 | 2 | 1 | Pup | 1 |  |
| 5 | KTI | 5 | 2 | 9 | MO | 1 |  |
| 5 | KTI | 5 | 2 | 9 | MO | 1 |  |
| 5 | KTI | 5 | 2 | 3 | MO | 1 |  |
| 5 | KTI | 5 | 2 | 3 | MO | 1 |  |
| 5 | KTI | 5 | 2 | 1 | MO | 1 |  |
| 5 | KTI | 5 | 2 | 1 | MO | 1 |  |
| 5 | KTI | 5 | 2 | 5 | Pup | 1 |  |
| 5 | KTI | 5 | 2 | 5 | Pup | 1 |  |
| 5 | KTI | 5 | 2 | 3 | Pup | 1 |  |
| 5 | KTI | 5 | 2 | 4 | Pup | 1 |  |
| 5 | KTI | 5 | 2 | 4 | Pup | 1 |  |
| 5 | KTI | 5 | 2 | 6 | Pup | 1 |  |
| 5 | KTI | 5 | 2 | 0.5 | Pup | 1 |  |
| 5 | KTI | 5 | 2 | 0.5 | Pup | 1 |  |
| 5 | KTI | 5 | 2 | 9 | Pup | 1 |  |
| 5 | KTI | 5 | 2 | 9 | Pup | 1 |  |
| 5 | KTI | 5 | 2 | 1 | Pup | 1 |  |
| 5 | KTI | 5 | 2 | 1 | Pup | 1 |  |
| 5 | KTI | 5 | 2 | 2 | Pup | 1 |  |
| 5 | KTI | 5 | 2 | 5 | Pup | 1 |  |
| 5 | KTI | 5 | 2 | 0.5 | MO | 1 |  |
| 5 | KTI | 5 | 2 | 1 | MO | 1 |  |
| 5 | BBR | 5 | 4 | 0.5 | MO | 1 |  |
| 5 | BBR | 5 | 4 | 0.5 | MO | 1 |  |
| 5 | BBR | 5 | 4 | 0.5 | MO | 1 |  |
| 5 | BBR | 5 | 4 | 0.5 | MO | 1 |  |
| 5 | BBR | 5 | 4 | 9 | Pup | 1 |  |
| 5 | BBR | 5 | 4 | 8 | Pup | 1 |  |
| 5 | BBR | 5 | 4 | 9 | Pup | 1 |  |
| 5 | BBR | 5 | 4 | 10 | Pup | 1 |  |
| 5 | BBR | 5 | 4 | 2 | Pup | 1 |  |
| 5 | BBR | 5 | 4 | 5 | Pup | 1 |  |
| 5 | BBR | 5 | 4 | 4 | Pup | 1 |  |
| 5 | BBR | 5 | 4 | 4 | Pup | 1 |  |
| 5 | BBR | 5 | 4 | 4 | Pup | 1 |  |
| 5 | BBR | 5 | 4 | 2 | Pup | 1 |  |
| 5 | BBR | 5 | 4 | 1 | Pup | 1 |  |
| 5 | BBR | 5 | 4 | 8 | MO | 1 |  |
| 5 | BBR | 5 | 4 | 8 | MO | 1 |  |
| 5 | BBR | 5 | 4 | 8 | MO | 1 |  |
| 5 | BBR | 5 | 4 | 8 | MO | 1 |  |
| 5 | BBR | 5 | 4 | 1 | Pup | 1 |  |
| 5 | BBR | 5 | 4 | 5 | Pup | 1 |  |
| 5 | BBR | 5 | 4 | 9 | Pup | 1 |  |
| 5 | BBR | 5 | 4 | 4 | Pup | 1 |  |
| 5 | BBR | 5 | 4 | 4 | Pup | 1 |  |
| 5 | BBR | 5 | 4 | 4 | Pup | 1 |  |
| 5 | BBR | 5 | 4 | 5 | MO | 1 |  |
| 5 | BBR | 5 | 4 | 5 | MO | 1 |  |
| 5 | BBR | 5 | 4 | 5 | MO | 1 |  |
| 5 | BBR | 5 | 4 | 5 | MO | 1 |  |
| 5 | BBR | 5 | 4 | 8 | MO | 1 |  |
| 5 | BBR | 5 | 4 | 8 | MO | 1 |  |
| 5 | BBR | 5 | 4 | 5 | MO | 1 |  |
| 5 | BBR | 5 | 4 | 8 | MO | 1 |  |
| 5 | BBR | 5 | 4 | 8 | MO | 1 |  |
| 5 | BBR | 5 | 4 | 8 | MO | 1 |  |
| 5 | BBR | 5 | 4 | 8 | MO | 1 |  |
| 5 | BBR | 5 | 4 | 8 | MO | 1 |  |
| 5 | BBR | 5 | 4 | 2 | Pup | 1 |  |
| 5 | BBR | 5 | 4 | 6 | MO | 1 |  |
| 5 | BBR | 5 | 4 | 6 | MO | 1 |  |
| 5 | BBR | 5 | 4 | 6 | MO | 1 |  |
| 5 | BBR | 5 | 4 | 6 | MO | 1 |  |
| 5 | WHI | 5 | 2 | 7 | MO | 1 |  |
| 5 | WHI | 5 | 2 | 9 | MO | 1 |  |
| 5 | WHI | 5 | 2 | 2 | Pup | 1 |  |
| 5 | WHI | 5 | 2 | 1 | Pup | 1 |  |
| 5 | WHI | 5 | 2 | 1 | Pup | 1 |  |
| 5 | WHI | 5 | 2 | 2 | Pup | 1 |  |
| 5 | WHI | 5 | 2 | 6 | Pup | 1 |  |
| 5 | WHI | 5 | 2 | 4 | MO | 1 |  |
| 5 | WHI | 5 | 2 | 4 | MO | 1 |  |
| 5 | WHI | 5 | 2 | 8 | MO | 1 |  |
| 5 | WHI | 5 | 2 | 10 | MO | 1 |  |
| 5 | WHI | 5 | 2 | 1 | Pup | 1 |  |
| 5 | WHI | 5 | 2 | 8 | Pup | 1 |  |
| 5 | WHI | 5 | 2 | 6 | Pup | 1 |  |
| 5 | WHI | 5 | 2 | 3 | MO | 1 |  |
| 5 | WHI | 5 | 2 | 5 | MO | 1 |  |
| 5 | WHI | 5 | 2 | 6 | Pup | 1 |  |
| 5 | WHI | 5 | 2 | 7 | Pup | 1 |  |
| 5 | WHI | 5 | 2 | 3 | Pup | 1 |  |
| 5 | WHI | 5 | 2 | 7 | MO | 1 |  |
| 5 | WHI | 5 | 2 | 9 | MO | 1 |  |
| 5 | WHI | 5 | 2 | 9 | MO | 1 |  |
| 5 | WHI | 5 | 2 | 9 | MO | 1 |  |
| 5 | WHI | 5 | 2 | 2 | Pup | 1 |  |
| 5 | RS5 | 5 | 3 | 8 | MO | 1 |  |
| 5 | RS5 | 5 | 3 | 8 | MO | 1 |  |
| 5 | RS5 | 5 | 3 | 8 | MO | 1 |  |
| 5 | RS5 | 5 | 3 | 9 | MO | 1 |  |
| 5 | RS5 | 5 | 3 | 9 | MO | 1 |  |
| 5 | RS5 | 5 | 3 | 9 | MO | 1 |  |
| 5 | RS5 | 5 | 3 | 3 | MO | 1 |  |
| 5 | RS5 | 5 | 3 | 3 | MO | 1 |  |
| 5 | RS5 | 5 | 3 | 6 | MO | 1 |  |
| 5 | RS5 | 5 | 3 | 2 | Pup | 1 |  |
| 5 | RS5 | 5 | 3 | 2 | Pup | 1 |  |
| 5 | RS5 | 5 | 3 | 2 | Pup | 1 |  |
| 5 | RS5 | 5 | 3 | 7 | Pup | 1 |  |
| 5 | RS5 | 5 | 3 | 6 | Pup | 1 |  |
| 5 | RS5 | 5 | 3 | 4 | Pup | 1 |  |
| 5 | RS5 | 5 | 3 | 2 | Pup | 1 |  |
| 5 | RS5 | 5 | 3 | 9 | Pup | 1 |  |
| 5 | RS5 | 5 | 3 | 9 | Pup | 1 |  |
| 5 | RS5 | 5 | 3 | 10 | Pup | 1 |  |
| 5 | RS5 | 5 | 3 | 3 | Pup | 1 |  |
| 5 | RS5 | 5 | 3 | 1 | Pup | 1 |  |
| 5 | RS5 | 5 | 3 | 1 | Pup | 1 |  |
| 5 | RS5 | 5 | 3 | 3 | Pup | 1 |  |
| 5 | RS5 | 5 | 3 | 1 | Pup | 1 |  |
| 5 | RS5 | 5 | 3 | 6 | MO | 1 |  |
| 5 | RS5 | 5 | 3 | 5 | MO | 1 |  |
| 5 | RS5 | 5 | 3 | 5 | MO | 1 |  |
| 4 | CAN | 6 | 6 | 1 | Pup | 1 |  |
| 4 | CAN | 6 | 6 | 3 | MO | 1 |  |
| 4 | CAN | 6 | 6 | 8 | MO | 1 |  |
| 4 | CAN | 6 | 6 | 8 | MO | 1 |  |
| 4 | CAN | 6 | 6 | 12 | MO | 1 |  |
| 4 | CAN | 6 | 6 | 8 | MO | 1 |  |
| 4 | CAN | 6 | 6 | 12 | MO | 1 |  |
| 4 | CAN | 6 | 6 | 12 | MO | 1 |  |
| 4 | CAN | 6 | 6 | 4 | Pup | 1 |  |
| 4 | CAN | 6 | 6 | 4 | Pup | 1 |  |
| 4 | CAN | 6 | 6 | 3 | Pup | 1 |  |
| 4 | MDB | 6 | 5 | 0.5 | Pup | 1 |  |
| 4 | MDB | 6 | 5 | 0.5 | Pup | 1 |  |
| 4 | MDB | 6 | 5 | 0.5 | Pup | 1 |  |
| 4 | MDB | 6 | 5 | 1 | Pup | 1 |  |
| 4 | MDB | 6 | 5 | 1 | Pup | 1 |  |
| 4 | MDB | 6 | 5 | 1 | Pup | 1 |  |
| 4 | MDB | 6 | 5 | 1 | Pup | 1 |  |
| 4 | MDB | 6 | 5 | 0.5 | Pup | 1 |  |
| 4 | MDB | 6 | 5 | 0.5 | Pup | 1 |  |
| 4 | MDB | 6 | 5 | 0.5 | Pup | 1 |  |
| 4 | MDB | 6 | 5 | 0.5 | Pup | 1 |  |
| 4 | MDB | 6 | 5 | 0.5 | Pup | 1 |  |
| 4 | MDB | 6 | 5 | 3 | Pup | 1 |  |
| 4 | MDB | 6 | 5 | 3 | Pup | 1 |  |
| 4 | MDB | 6 | 5 | 3 | Pup | 1 |  |
| 4 | MDB | 6 | 5 | 3 | Pup | 1 |  |
| 4 | MDB | 6 | 5 | 3 | Pup | 1 |  |
| 4 | MDB | 6 | 5 | 1 | Pup | 1 |  |
| 4 | MDB | 6 | 5 | 1 | Pup | 1 |  |
| 4 | MDB | 6 | 5 | 1 | Pup | 1 |  |
| 4 | MDB | 6 | 5 | 0.5 | Pup | 1 |  |
| 4 | MDB | 6 | 5 | 0.5 | Pup | 1 |  |
| 4 | MDB | 6 | 5 | 0.5 | Pup | 1 |  |
| 4 | MDB | 6 | 5 | 0.5 | Pup | 1 |  |
| 4 | MDB | 6 | 5 | 0.5 | Pup | 1 |  |
| 4 | MDB | 6 | 5 | 0.5 | Pup | 1 |  |
| 4 | MDB | 6 | 5 | 0.5 | Pup | 1 |  |
| 4 | MDB | 6 | 5 | 1 | MO | 1 |  |
| 4 | MDB | 6 | 5 | 1 | MO | 1 |  |
| 4 | MDB | 6 | 5 | 1 | MO | 1 |  |
| 4 | MDB | 6 | 5 | 1 | MO | 1 |  |
| 4 | MDB | 6 | 5 | 1 | MO | 1 |  |
| 4 | MDB | 6 | 5 | 1 | MO | 1 |  |
| 4 | MDB | 6 | 5 | 1 | MO | 1 |  |
| 4 | MDB | 6 | 5 | 1 | MO | 1 |  |
| 4 | MDB | 6 | 5 | 1 | MO | 1 |  |
| 4 | MDB | 6 | 5 | 1 | MO | 1 |  |
| 4 | MDB | 6 | 5 | 0.5 | Pup | 1 |  |
| 4 | MDB | 6 | 5 | 0.5 | Pup | 1 |  |
| 4 | PF1 | 6 | 3 | 5 | Pup |  | 1 |
| 4 | PF1 | 6 | 3 | 11 | Pup |  | 1 |
| 4 | PF1 | 6 | 3 | 5 | Pup |  | 1 |
| 4 | PF1 | 6 | 3 | 1 | Pup |  | 1 |
| 4 | PF1 | 6 | 3 | 8 | Pup | 1 |  |
| 4 | PF1 | 6 | 3 | 7 | Pup | 1 |  |
| 4 | PF1 | 6 | 3 | 7 | Pup | 1 |  |
| 4 | PF1 | 6 | 3 | 1 | Pup | 1 |  |
| 4 | PF1 | 6 | 3 | 2 | Pup | 1 |  |
| 4 | PF1 | 6 | 3 | 1 | Pup | 1 |  |
| 4 | PF1 | 6 | 3 | 6 | Pup | 1 |  |
| 4 | PF1 | 6 | 3 | 6 | Pup | 1 |  |
| 4 | PF1 | 6 | 3 | 5 | Pup | 1 |  |
| 4 | PF1 | 6 | 3 | 0.5 | Pup | 1 |  |
| 4 | PF1 | 6 | 3 | 0.5 | Pup | 1 |  |
| 4 | PF1 | 6 | 3 | 0.5 | Pup | 1 |  |
| 4 | RS1 | 6 | 2 | 3 | MO | 1 |  |
| 4 | RS1 | 6 | 2 | 3 | MO | 1 |  |
| 4 | RS1 | 6 | 2 | 3 | Pup | 1 |  |
| 4 | RS1 | 6 | 2 | 3 | Pup | 1 |  |
| 4 | RS1 | 6 | 2 | 1 | Pup | 1 |  |
| 4 | RS1 | 6 | 2 | 1 | Pup | 1 |  |
| 4 | RS1 | 6 | 2 | 1 | Pup | 1 |  |
| 4 | RS2 | 6 | 4 | 4 | Pup | 1 |  |
| 4 | RS2 | 6 | 4 | 4 | Pup | 1 |  |
| 4 | RS2 | 6 | 4 | 4 | Pup | 1 |  |
| 4 | RS2 | 6 | 4 | 4 | Pup | 1 |  |
| 4 | RS2 | 6 | 4 | 3 | Pup | 1 |  |
| 4 | RS2 | 6 | 4 | 3 | Pup | 1 |  |
| 4 | RS2 | 6 | 4 | 3 | Pup | 1 |  |
| 4 | RS2 | 6 | 4 | 3 | Pup | 1 |  |
| 4 | RS2 | 6 | 4 | 3 | Pup | 1 |  |
| 4 | RS2 | 6 | 4 | 1 | Pup | 1 |  |
| 4 | RS2 | 6 | 4 | 1 | Pup | 1 |  |
| 4 | RS2 | 6 | 4 | 1 | Pup | 1 |  |
| 4 | RS2 | 6 | 4 | 1 | Pup | 1 |  |
| 4 | RS2 | 6 | 4 | 1 | Pup | 1 |  |
| 4 | RS2 | 6 | 4 | 0.5 | Pup | 1 |  |
| 4 | RS2 | 6 | 4 | 5 | Pup | 1 |  |
| 4 | RS2 | 6 | 4 | 5 | Pup | 1 |  |
| 4 | RS2 | 6 | 4 | 5 | Pup | 1 |  |
| 4 | RS2 | 6 | 4 | 5 | Pup | 1 |  |
| 4 | RS2 | 6 | 4 | 3 | Pup | 1 |  |
| 4 | RS2 | 6 | 4 | 0.5 | Pup | 1 |  |
| 4 | RS2 | 6 | 4 | 0.5 | Pup | 1 |  |
| 4 | RS2 | 6 | 4 | 3 | Pup | 1 |  |
| 4 | RS2 | 6 | 4 | 2 | Pup | 1 |  |
| 4 | RS2 | 6 | 4 | 5 | Pup | 1 |  |
| 4 | RS2 | 6 | 4 | 3 | Pup | 1 |  |
| 4 | RS2 | 6 | 4 | 3 | Pup | 1 |  |
| 4 | RS2 | 6 | 4 | 1 | Pup | 1 |  |
| 4 | RS2 | 6 | 4 | 1 | Pup | 1 |  |
| 4 | RS2 | 6 | 4 | 1 | Pup | 1 |  |
| 4 | RS2 | 6 | 4 | 1 | Pup | 1 |  |
| 4 | RS2 | 6 | 4 | 3 | Pup | 1 |  |
| 4 | RS2 | 6 | 4 | 3 | Pup | 1 |  |
| 4 | RS2 | 6 | 4 | 3 | Pup | 1 |  |
| 4 | RS2 | 6 | 4 | 0.5 | Pup | 1 |  |
| 4 | RS2 | 6 | 4 | 0.5 | Pup | 1 |  |
| 4 | RS2 | 6 | 4 | 1 | Pup | 1 |  |
| 4 | RS2 | 6 | 4 | 1 | Pup | 1 |  |
| 4 | RS2 | 6 | 4 | 1 | Pup | 1 |  |
| 4 | RS2 | 6 | 4 | 1 | Pup | 1 |  |
| 4 | RS2 | 6 | 4 | 0.5 | Pup | 1 |  |
| 4 | RS2 | 6 | 4 | 0.5 | Pup | 1 |  |
| 4 | RS2 | 6 | 4 | 1 | Pup | 1 |  |
| 4 | RS2 | 6 | 4 | 1 | Pup | 1 |  |
| 4 | RS2 | 6 | 4 | 3 | Pup | 1 |  |
| 4 | RS2 | 6 | 4 | 2 | Pup | 1 |  |
| 4 | RS2 | 6 | 4 | 2 | Pup | 1 |  |
| 4 | RS2 | 6 | 4 | 1 | Pup | 1 |  |
| 4 | RS2 | 6 | 4 | 1 | Pup | 1 |  |
| 4 | RS2 | 6 | 4 | 0.5 | Pup | 1 |  |
| 4 | RS2 | 6 | 4 | 0.5 | Pup | 1 |  |
| 4 | RS2 | 6 | 4 | 1 | Pup | 1 |  |
| 4 | RS2 | 6 | 4 | 1 | Pup | 1 |  |
| 4 | RS2 | 6 | 4 | 0.5 | Pup | 1 |  |
| 4 | RS2 | 6 | 4 | 2 | Pup | 1 |  |
| 4 | RS2 | 6 | 4 | 2 | Pup | 1 |  |
| 4 | RS2 | 6 | 4 | 3 | Pup | 1 |  |
| 4 | RS2 | 6 | 4 | 3 | Pup | 1 |  |
| 4 | RS2 | 6 | 4 | 3 | Pup | 1 |  |
| 4 | RS2 | 6 | 4 | 3 | Pup | 1 |  |
| 4 | RS2 | 6 | 4 | 3 | Pup | 1 |  |
| 4 | RS2 | 6 | 4 | 3 | MO | 1 |  |
| 4 | RS2 | 6 | 4 | 3 | MO | 1 |  |
| 4 | RS2 | 6 | 4 | 3 | MO | 1 |  |
| 4 | RS2 | 6 | 4 | 3 | MO | 1 |  |
| 4 | RS2 | 6 | 4 | 1 | Pup | 1 |  |
| 4 | RS2 | 6 | 4 | 1 | Pup | 1 |  |
| 4 | RS2 | 6 | 4 | 1 | Pup | 1 |  |
| 4 | RS2 | 6 | 4 | 1 | Pup | 1 |  |
| 4 | RS2 | 6 | 4 | 6 | MO | 1 |  |
| 4 | RS2 | 6 | 4 | 6 | MO | 1 |  |
| 4 | RS2 | 6 | 4 | 6 | MO | 1 |  |
| 4 | RS2 | 6 | 4 | 6 | MO | 1 |  |
| 4 | RS2 | 6 | 4 | 6 | Pup | 1 |  |
| 4 | RS2 | 6 | 4 | 6 | Pup | 1 |  |
| 4 | RS2 | 6 | 4 | 6 | Pup | 1 |  |
| 4 | RS2 | 6 | 4 | 6 | Pup | 1 |  |
| 4 | RS2 | 6 | 4 | 2 | Pup | 1 |  |
| 4 | RS2 | 6 | 4 | 1 | Pup | 1 |  |
| 4 | RS2 | 6 | 4 | 1 | Pup | 1 |  |
| 4 | RS2 | 6 | 4 | 1 | Pup | 1 |  |
| 4 | RS2 | 6 | 4 | 1 | Pup | 1 |  |
| 4 | RS2 | 6 | 4 | 1 | Pup | 1 |  |
| 4 | RS2 | 6 | 4 | 1 | Pup | 1 |  |
| 4 | RS2 | 6 | 4 | 1 | Pup | 1 |  |
| 4 | RS2 | 6 | 4 | 1 | Pup | 1 |  |
| 4 | RS2 | 6 | 4 | 1 | Pup | 1 |  |
| 4 | RS2 | 6 | 4 | 0.5 | Pup | 1 |  |
| 4 | RS2 | 6 | 4 | 0.5 | Pup | 1 |  |
| 4 | RS2 | 6 | 4 | 1 | Pup | 1 |  |
| 4 | RS2 | 6 | 4 | 1 | Pup | 1 |  |
| 4 | RS2 | 6 | 4 | 4 | Pup | 1 |  |
| 4 | RS2 | 6 | 4 | 4 | Pup | 1 |  |
| 4 | RS2 | 6 | 4 | 4 | Pup | 1 |  |
| 4 | RS2 | 6 | 4 | 4 | Pup | 1 |  |
| 4 | RS2 | 6 | 4 | 4 | Pup | 1 |  |
| 4 | RS2 | 6 | 4 | 2 | Pup | 1 |  |
| 4 | RS2 | 6 | 4 | 2 | Pup | 1 |  |
| 4 | RS2 | 6 | 4 | 2 | Pup | 1 |  |
| 4 | RS2 | 6 | 4 | 2 | Pup | 1 |  |
| 4 | RS2 | 6 | 4 | 2 | Pup | 1 |  |
| 4 | RS2 | 6 | 4 | 1 | Pup | 1 |  |
| 4 | RS2 | 6 | 4 | 2 | Pup | 1 |  |
| 4 | RS2 | 6 | 4 | 2 | Pup | 1 |  |
| 4 | RS2 | 6 | 4 | 3 | Pup | 1 |  |
| 4 | RS2 | 6 | 4 | 1 | Pup | 1 |  |
| 4 | RS2 | 6 | 4 | 1 | Pup | 1 |  |
| 4 | RS2 | 6 | 4 | 2 | Pup | 1 |  |
| 4 | RS2 | 6 | 4 | 2 | Pup | 1 |  |
| 4 | RS2 | 6 | 4 | 2 | Pup | 1 |  |
| 4 | RS2 | 6 | 4 | 2 | Pup | 1 |  |
| 4 | RS2 | 6 | 4 | 2 | Pup | 1 |  |
| 4 | RS2 | 6 | 4 | 1 | Pup | 1 |  |
| 4 | RS2 | 6 | 4 | 1 | Pup | 1 |  |
| 4 | RS2 | 6 | 4 | 1 | Pup | 1 |  |
| 4 | RS2 | 6 | 4 | 1 | Pup | 1 |  |
| 4 | RS2 | 6 | 4 | 1 | Pup |  | 1 |
| 4 | RS2 | 6 | 4 | 1 | Pup |  | 1 |
| 4 | RS2 | 6 | 4 | 1 | Pup |  | 1 |
| 4 | RS2 | 6 | 4 | 1 | Pup |  | 1 |
| 4 | RS2 | 6 | 4 | 1 | Pup |  | 1 |
| 4 | RS3 | 6 | 2 | 6 | MO | 1 |  |
| 4 | RS3 | 6 | 2 | 5 | MO | 1 |  |
| 4 | RS3 | 6 | 2 | 1 | Pup |  | 1 |
| 4 | RS3 | 6 | 2 | 1 | Pup |  | 1 |
| 4 | RS3 | 6 | 2 | 1 | Pup |  | 1 |
| 4 | RS3 | 6 | 2 | 3 | Pup |  | 1 |
| 4 | RS3 | 6 | 2 | 1 | Pup |  | 1 |
| 4 | RS3 | 6 | 2 | 2 | Pup | 1 |  |
| 4 | RS3 | 6 | 2 | 0.5 | Pup | 1 |  |
| 4 | RS3 | 6 | 2 | 1 | Pup | 1 |  |
| 4 | RS3 | 6 | 2 | 2 | Pup | 1 |  |
| 5 | BRN | 6 | 1 | 2 | Pup | 1 |  |
| 5 | BRN | 6 | 1 | 1 | Pup | 1 |  |
| 5 | BRN | 6 | 1 | 0.5 | Pup | 1 |  |
| 5 | BRN | 6 | 1 | 4 | Pup | 1 |  |
| 5 | BRN | 6 | 1 | 4 | Pup | 1 |  |
| 5 | BRN | 6 | 1 | 2 | Pup | 1 |  |
| 5 | BRN | 6 | 1 | 3 | Pup | 1 |  |
| 5 | BRN | 6 | 1 | 6 | Pup | 1 |  |
| 5 | BRN | 6 | 1 | 5 | Pup | 1 |  |
| 5 | BRN | 6 | 1 | 0.5 | Pup | 1 |  |
| 5 | BRN | 6 | 1 | 0.5 | Pup | 1 |  |
| 5 | BRN | 6 | 1 | 3 | Pup | 1 |  |
| 5 | BRN | 6 | 1 | 2 | Pup | 1 |  |
| 5 | BRN | 6 | 1 | 1 | Pup | 1 |  |
| 5 | BRN | 6 | 1 | 1 | Pup | 1 |  |
| 5 | BRN | 6 | 1 | 5 | Pup | 1 |  |
| 5 | BRN | 6 | 1 | 5 | Pup | 1 |  |
| 5 | KTI | 6 | 2 | 2 | Pup | 1 |  |
| 5 | KTI | 6 | 2 | 1 | Pup | 1 |  |
| 5 | KTI | 6 | 2 | 1 | Pup |  | 1 |
| 5 | KTI | 6 | 2 | 1 | Pup |  | 1 |
| 5 | KTI | 6 | 2 | 2 | Pup |  | 1 |
| 5 | KTI | 6 | 2 | 2 | Pup |  | 1 |
| 5 | KTI | 6 | 2 | 2 | Pup |  | 1 |
| 5 | KTI | 6 | 2 | 4 | Pup | 1 |  |
| 5 | KTI | 6 | 2 | 4 | Pup | 1 |  |
| 5 | KTI | 6 | 2 | 1 | Pup |  | 1 |
| 5 | KTI | 6 | 2 | 0.5 | Pup | 1 |  |
| 5 | KTI | 6 | 2 | 1 | Pup | 1 |  |
| 5 | KTI | 6 | 2 | 2 | Pup | 1 |  |
| 5 | KTI | 6 | 2 | 1 | Pup |  | 1 |
| 5 | KTI | 6 | 2 | 1 | Pup | 1 |  |
| 5 | KTI | 6 | 2 | 1 | Pup | 1 |  |
| 5 | KTI | 6 | 2 | 1 | Pup | 1 |  |
| 5 | KTI | 6 | 2 | 1 | Pup | 1 |  |
| 5 | BBR | 6 | 4 | 5 | MO | 1 |  |
| 5 | BBR | 6 | 4 | 6 | MO | 1 |  |
| 5 | BBR | 6 | 4 | 8 | MO | 1 |  |
| 5 | BBR | 6 | 4 | 8 | MO | 1 |  |
| 5 | BBR | 6 | 4 | 4 | Pup | 1 |  |
| 5 | BBR | 6 | 4 | 2 | Pup | 1 |  |
| 5 | BBR | 6 | 4 | 1 | Pup | 1 |  |
| 5 | BBR | 6 | 4 | 6 | MO | 1 |  |
| 5 | BBR | 6 | 4 | 6 | MO | 1 |  |
| 5 | BBR | 6 | 4 | 6 | MO | 1 |  |
| 5 | BBR | 6 | 4 | 6 | MO | 1 |  |
| 5 | BBR | 6 | 4 | 8 | MO | 1 |  |
| 5 | BBR | 6 | 4 | 8 | MO | 1 |  |
| 5 | BBR | 6 | 4 | 6 | MO | 1 |  |
| 5 | BBR | 6 | 4 | 8 | MO | 1 |  |
| 5 | BBR | 6 | 4 | 1 | Pup |  | 1 |
| 5 | BBR | 6 | 4 | 1 | Pup |  | 1 |
| 5 | BBR | 6 | 4 | 3 | Pup |  | 1 |
| 5 | BBR | 6 | 4 | 3 | Pup |  | 1 |
| 5 | BBR | 6 | 4 | 3 | Pup |  | 1 |
| 5 | BBR | 6 | 4 | 4 | Pup | 1 |  |
| 5 | BBR | 6 | 4 | 4 | Pup | 1 |  |
| 5 | BBR | 6 | 4 | 1 | Pup | 1 |  |
| 5 | BBR | 6 | 4 | 2 | Pup |  | 1 |
| 5 | BBR | 6 | 4 | 2 | Pup |  | 1 |
| 5 | BBR | 6 | 4 | 2 | Pup |  | 1 |
| 5 | BBR | 6 | 4 | 4 | Pup | 1 |  |
| 5 | BBR | 6 | 4 | 4 | Pup | 1 |  |
| 5 | BBR | 6 | 4 | 3 | Pup | 1 |  |
| 5 | BBR | 6 | 4 | 3 | Pup | 1 |  |
| 5 | BBR | 6 | 4 | 3 | Pup | 1 |  |
| 5 | BBR | 6 | 4 | 5 | Pup | 1 |  |
| 5 | BBR | 6 | 4 | 5 | Pup | 1 |  |
| 5 | BBR | 6 | 4 | 5 | Pup | 1 |  |
| 5 | BBR | 6 | 4 | 5 | Pup | 1 |  |
| 5 | BBR | 6 | 4 | 5 | Pup | 1 |  |
| 5 | BBR | 6 | 4 | 1 | Pup |  | 1 |
| 5 | BBR | 6 | 4 | 1 | Pup |  | 1 |
| 5 | BBR | 6 | 4 | 1 | Pup |  | 1 |
| 5 | BBR | 6 | 4 | 3 | Pup |  | 1 |
| 5 | BBR | 6 | 4 | 3 | Pup |  | 1 |
| 5 | BBR | 6 | 4 | 3 | Pup |  | 1 |
| 5 | BBR | 6 | 4 | 5 | Pup | 1 |  |
| 5 | BBR | 6 | 4 | 4 | Pup | 1 |  |
| 5 | BBR | 6 | 4 | 4 | Pup | 1 |  |
| 5 | BBR | 6 | 4 | 3 | Pup | 1 |  |
| 5 | BBR | 6 | 4 | 1 | Pup | 1 |  |
| 5 | BBR | 6 | 4 | 1 | Pup | 1 |  |
| 5 | BBR | 6 | 4 | 3 | Pup |  | 1 |
| 5 | BBR | 6 | 4 | 3 | Pup |  | 1 |
| 5 | BBR | 6 | 4 | 3 | Pup |  | 1 |
| 5 | WHI | 6 | 2 | 4 | MO | 1 |  |
| 5 | WHI | 6 | 2 | 6 | MO | 1 |  |
| 5 | WHI | 6 | 2 | 9 | Pup | 1 |  |
| 5 | WHI | 6 | 2 | 5 | Pup | 1 |  |
| 5 | WHI | 6 | 2 | 1 | Pup | 1 |  |
| 5 | WHI | 6 | 2 | 8 | MO | 1 |  |
| 5 | WHI | 6 | 2 | 9 | MO | 1 |  |
| 5 | WHI | 6 | 2 | 2 | Pup | 1 |  |
| 5 | WHI | 6 | 2 | 1 | Pup | 1 |  |
| 5 | WHI | 6 | 2 | 10 | Pup | 1 |  |
| 5 | WHI | 6 | 2 | 9 | Pup | 1 |  |
| 5 | WHI | 6 | 2 | 6 | MO | 1 |  |
| 5 | WHI | 6 | 2 | 6 | MO | 1 |  |
| 5 | WHI | 6 | 2 | 7 | MO | 1 |  |
| 5 | WHI | 6 | 2 | 9 | MO | 1 |  |
| 5 | WHI | 6 | 2 | 2 | Pup | 1 |  |
| 5 | WHI | 6 | 2 | 3 | Pup | 1 |  |
| 5 | WHI | 6 | 2 | 7 | Pup | 1 |  |
| 5 | WHI | 6 | 2 | 5 | Pup | 1 |  |
| 5 | WHI | 6 | 2 | 8 | MO | 1 |  |
| 5 | WHI | 6 | 2 | 11 | MO | 1 |  |
| 5 | RS5 | 6 | 3 | 3 | Pup | 1 |  |
| 5 | RS5 | 6 | 3 | 3 | Pup | 1 |  |
| 5 | RS5 | 6 | 3 | 3 | Pup | 1 |  |
| 5 | RS5 | 6 | 3 | 5 | Pup | 1 |  |
| 5 | RS5 | 6 | 3 | 5 | Pup | 1 |  |
| 5 | RS5 | 6 | 3 | 5 | Pup | 1 |  |
| 5 | RS5 | 6 | 3 | 3 | MO | 1 |  |
| 5 | RS5 | 6 | 3 | 3 | MO | 1 |  |
| 5 | RS5 | 6 | 3 | 2 | MO | 1 |  |
| 5 | RS5 | 6 | 3 | 2 | Pup | 1 |  |
| 5 | RS5 | 6 | 3 | 5 | Pup | 1 |  |
| 5 | RS5 | 6 | 3 | 5 | Pup | 1 |  |
| 5 | RS5 | 6 | 3 | 4 | Pup | 1 |  |
| 5 | RS5 | 6 | 3 | 2 | Pup | 1 |  |
| 5 | RS5 | 6 | 3 | 2 | Pup | 1 |  |
| 5 | RS5 | 6 | 3 | 2 | Pup | 1 |  |
| 5 | RS5 | 6 | 3 | 8 | Pup | 1 |  |
| 5 | RS5 | 6 | 3 | 3 | Pup | 1 |  |
| 5 | RS5 | 6 | 3 | 7 | Pup | 1 |  |
| 5 | RS5 | 6 | 3 | 5 | Pup | 1 |  |
| 5 | RS5 | 6 | 3 | 3 | Pup | 1 |  |
| 5 | RS5 | 6 | 3 | 2 | Pup | 1 |  |
| 5 | RS5 | 6 | 3 | 2 | Pup | 1 |  |
| 5 | RS5 | 6 | 3 | 2 | Pup | 1 |  |
| 5 | RS5 | 6 | 3 | 2 | Pup | 1 |  |
| 4 | CAN | 7 | 5 | 19 | MO | 1 |  |
| 4 | CAN | 7 | 5 | 19 | MO | 1 |  |
| 4 | CAN | 7 | 5 | 19 | MO | 1 |  |
| 4 | CAN | 7 | 5 | 19 | MO | 1 |  |
| 4 | CAN | 7 | 5 | 19 | MO | 1 |  |
| 4 | CAN | 7 | 5 | 14 | Pup | 1 |  |
| 4 | CAN | 7 | 5 | 14 | Pup | 1 |  |
| 4 | CAN | 7 | 5 | 13 | Pup | 1 |  |
| 4 | CAN | 7 | 5 | 13 | Pup | 1 |  |
| 4 | CAN | 7 | 5 | 13 | Pup | 1 |  |
| 4 | CAN | 7 | 5 | 11 | MO | 1 |  |
| 4 | CAN | 7 | 5 | 11 | MO | 1 |  |
| 4 | CAN | 7 | 5 | 11 | MO | 1 |  |
| 4 | CAN | 7 | 5 | 11 | MO | 1 |  |
| 4 | CAN | 7 | 5 | 11 | MO | 1 |  |
| 4 | PF1 | 7 | 3 | 0.5 | Pup |  | 1 |
| 4 | PF1 | 7 | 3 | 1 | Pup |  | 1 |
| 4 | PF1 | 7 | 3 | 0.5 | Pup |  | 1 |
| 4 | PF1 | 7 | 3 | 0.5 | Pup |  | 1 |
| 4 | PF1 | 7 | 3 | 1 | Pup | 1 |  |
| 4 | PF1 | 7 | 3 | 1 | Pup | 1 |  |
| 4 | PF1 | 7 | 3 | 2 | Pup | 1 |  |
| 4 | RS1 | 7 | 2 | 1 | Pup |  | 1 |
| 4 | RS1 | 7 | 2 | 1 | Pup |  | 1 |
| 4 | RS1 | 7 | 2 | 2 | Pup | 1 |  |
| 4 | RS1 | 7 | 2 | 2 | Pup | 1 |  |
| 4 | RS1 | 7 | 2 | 0.5 | Pup | 1 |  |
| 4 | RS1 | 7 | 2 | 1 | Pup |  | 1 |
| 4 | RS1 | 7 | 2 | 4 | Pup | 1 |  |
| 4 | RS1 | 7 | 2 | 4 | Pup | 1 |  |
| 4 | RS1 | 7 | 2 | 0.5 | Pup |  | 1 |
| 4 | RS1 | 7 | 2 | 1 | Pup |  | 1 |
| 4 | RS1 | 7 | 2 | 0.5 | Pup |  | 1 |
| 4 | RS1 | 7 | 2 | 12 | Pup |  | 1 |
| 4 | RS1 | 7 | 2 | 9 | Pup |  | 1 |
| 4 | RS1 | 7 | 2 | 1 | Pup |  | 1 |
| 4 | RS1 | 7 | 2 | 0.5 | Pup |  | 1 |
| 4 | RS1 | 7 | 2 | 0.5 | Pup |  | 1 |
| 4 | RS1 | 7 | 2 | 1 | Pup |  | 1 |
| 4 | RS1 | 7 | 2 | 0.5 | Pup |  | 1 |
| 4 | RS1 | 7 | 2 | 0.5 | Pup |  | 1 |
| 4 | RS1 | 7 | 2 | 0.5 | Pup |  | 1 |
| 4 | RS1 | 7 | 2 | 0.5 | Pup |  | 1 |
| 4 | RS1 | 7 | 2 | 1 | Pup |  | 1 |
| 4 | RS1 | 7 | 2 | 5 | Pup |  | 1 |
| 4 | RS1 | 7 | 2 | 3 | Pup |  | 1 |
| 4 | RS1 | 7 | 2 | 2 | Pup |  | 1 |
| 4 | RS1 | 7 | 2 | 0.5 | Pup |  | 1 |
| 4 | RS1 | 7 | 2 | 1 | Pup |  | 1 |
| 4 | RS1 | 7 | 2 | 0.5 | Pup |  | 1 |
| 4 | RS1 | 7 | 2 | 1 | Pup | 1 |  |
| 4 | RS1 | 7 | 2 | 1 | Pup | 1 |  |
| 4 | RS1 | 7 | 2 | 0.5 | Pup |  | 1 |
| 4 | RS1 | 7 | 2 | 2 | Pup |  | 1 |
| 4 | RS1 | 7 | 2 | 2 | Pup |  | 1 |
| 4 | RS1 | 7 | 2 | 0.5 | Pup |  | 1 |
| 4 | RS1 | 7 | 2 | 1 | Pup |  | 1 |
| 4 | RS1 | 7 | 2 | 1 | Pup |  | 1 |
| 4 | RS1 | 7 | 2 | 1 | Pup |  | 1 |
| 4 | RS1 | 7 | 2 | 1 | Pup |  | 1 |
| 4 | RS1 | 7 | 2 | 1 | Pup |  | 1 |
| 4 | RS1 | 7 | 2 | 1 | Pup |  | 1 |
| 4 | RS1 | 7 | 2 | 0.5 | Pup | 1 |  |
| 4 | RS1 | 7 | 2 | 0.5 | Pup | 1 |  |
| 4 | RS1 | 7 | 2 | 1 | Pup |  | 1 |
| 4 | RS1 | 7 | 2 | 1 | Pup | 1 |  |
| 4 | RS1 | 7 | 2 | 1 | Pup | 1 |  |
| 4 | RS1 | 7 | 2 | 0.5 | Pup | 1 |  |
| 4 | RS1 | 7 | 2 | 0.5 | Pup | 1 |  |
| 4 | RS1 | 7 | 2 | 0.5 | Pup | 1 |  |
| 4 | RS1 | 7 | 2 | 0.5 | Pup | 1 |  |
| 4 | RS1 | 7 | 2 | 1 | Pup | 1 |  |
| 4 | RS1 | 7 | 2 | 1 | Pup | 1 |  |
| 4 | RS1 | 7 | 2 | 0.5 | Pup | 1 |  |
| 4 | RS1 | 7 | 2 | 0.5 | Pup | 1 |  |
| 4 | RS1 | 7 | 2 | 3 | Pup |  | 1 |
| 4 | RS1 | 7 | 2 | 3 | Pup |  | 1 |
| 4 | RS1 | 7 | 2 | 2 | Pup | 1 |  |
| 4 | RS1 | 7 | 2 | 2 | Pup | 1 |  |
| 4 | RS1 | 7 | 2 | 2 | Pup | 1 |  |
| 4 | RS1 | 7 | 2 | 0.5 | Pup |  | 1 |
| 4 | RS1 | 7 | 2 | 0.5 | Pup |  | 1 |
| 4 | RS1 | 7 | 2 | 1 | Pup |  | 1 |
| 4 | RS1 | 7 | 2 | 1 | Pup |  | 1 |
| 4 | RS1 | 7 | 2 | 1 | Pup | 1 |  |
| 4 | RS1 | 7 | 2 | 1 | Pup | 1 |  |
| 4 | RS1 | 7 | 2 | 1 | Pup |  | 1 |
| 4 | RS1 | 7 | 2 | 1 | Pup |  | 1 |
| 4 | RS1 | 7 | 2 | 0.5 | Pup |  | 1 |
| 4 | RS1 | 7 | 2 | 0.5 | Pup |  | 1 |
| 4 | RS1 | 7 | 2 | 0.5 | Pup |  | 1 |
| 4 | RS1 | 7 | 2 | 0.5 | Pup |  | 1 |
| 4 | RS1 | 7 | 2 | 0.5 | Pup |  | 1 |
| 4 | RS1 | 7 | 2 | 0.5 | Pup |  | 1 |
| 4 | RS1 | 7 | 2 | 1 | Pup |  | 1 |
| 4 | RS1 | 7 | 2 | 1 | Pup |  | 1 |
| 4 | RS1 | 7 | 2 | 1 | Pup | 1 |  |
| 4 | RS1 | 7 | 2 | 1 | Pup | 1 |  |
| 4 | RS1 | 7 | 2 | 0.5 | Pup |  | 1 |
| 4 | RS1 | 7 | 2 | 0.5 | Pup |  | 1 |
| 4 | RS1 | 7 | 2 | 0.5 | Pup |  | 1 |
| 4 | RS1 | 7 | 2 | 0.5 | Pup |  | 1 |
| 4 | RS1 | 7 | 2 | 0.5 | Pup | 1 |  |
| 4 | RS1 | 7 | 2 | 0.5 | Pup | 1 |  |
| 4 | RS1 | 7 | 2 | 0.5 | Pup |  | 1 |
| 4 | RS1 | 7 | 2 | 0.5 | Pup |  | 1 |
| 4 | RS1 | 7 | 2 | 2 | Pup |  | 1 |
| 4 | RS1 | 7 | 2 | 0.5 | Pup |  | 1 |
| 4 | RS1 | 7 | 2 | 2 | Pup |  | 1 |
| 4 | RS1 | 7 | 2 | 1 | Pup |  | 1 |
| 4 | RS1 | 7 | 2 | 1 | Pup |  | 1 |
| 4 | RS1 | 7 | 2 | 1 | Pup |  | 1 |
| 4 | RS1 | 7 | 2 | 1 | Pup |  | 1 |
| 4 | RS1 | 7 | 2 | 1 | Pup |  | 1 |
| 4 | RS1 | 7 | 2 | 0.5 | Pup | 1 |  |
| 4 | RS1 | 7 | 2 | 1 | Pup |  | 1 |
| 4 | RS1 | 7 | 2 | 1 | Pup |  | 1 |
| 4 | RS1 | 7 | 2 | 1 | Pup |  | 1 |
| 4 | RS1 | 7 | 2 | 7 | Pup |  | 1 |
| 4 | RS1 | 7 | 2 | 7 | Pup |  | 1 |
| 4 | RS1 | 7 | 2 | 7 | Pup |  | 1 |
| 4 | RS1 | 7 | 2 | 3 | Pup |  | 1 |
| 4 | RS1 | 7 | 2 | 3 | Pup |  | 1 |
| 4 | RS1 | 7 | 2 | 3 | Pup |  | 1 |
| 4 | RS2 | 7 | 2 | 1 | Pup | 1 |  |
| 4 | RS2 | 7 | 2 | 6 | Pup | 1 |  |
| 4 | RS2 | 7 | 2 | 6 | Pup | 1 |  |
| 4 | RS2 | 7 | 2 | 1 | Pup | 1 |  |
| 4 | RS2 | 7 | 2 | 0.5 | Pup | 1 |  |
| 4 | RS2 | 7 | 2 | 1 | Pup | 1 |  |
| 4 | RS2 | 7 | 2 | 1 | Pup | 1 |  |
| 4 | RS2 | 7 | 2 | 1 | Pup | 1 |  |
| 4 | RS2 | 7 | 2 | 1 | Pup | 1 |  |
| 4 | RS2 | 7 | 2 | 3 | Pup | 1 |  |
| 4 | RS2 | 7 | 2 | 3 | Pup | 1 |  |
| 4 | RS2 | 7 | 2 | 3 | Pup | 1 |  |
| 4 | RS2 | 7 | 2 | 1 | Pup |  | 1 |
| 4 | RS2 | 7 | 2 | 1 | Pup |  | 1 |
| 4 | RS2 | 7 | 2 | 1 | Pup | 1 |  |
| 4 | RS2 | 7 | 2 | 6 | Pup | 1 |  |
| 4 | RS2 | 7 | 2 | 6 | Pup | 1 |  |
| 4 | RS2 | 7 | 2 | 5 | Pup | 1 |  |
| 4 | RS2 | 7 | 2 | 1 | Pup | 1 |  |
| 4 | RS2 | 7 | 2 | 2 | Pup | 1 |  |
| 4 | RS2 | 7 | 2 | 2 | Pup | 1 |  |
| 4 | RS2 | 7 | 2 | 1 | Pup | 1 |  |
| 4 | RS2 | 7 | 2 | 1 | Pup | 1 |  |
| 4 | RS2 | 7 | 2 | 1 | Pup | 1 |  |
| 4 | RS2 | 7 | 2 | 1 | Pup | 1 |  |
| 4 | RS2 | 7 | 2 | 1 | Pup | 1 |  |
| 4 | RS2 | 7 | 2 | 1 | Pup | 1 |  |
| 4 | RS2 | 7 | 2 | 2 | Pup | 1 |  |
| 4 | RS2 | 7 | 2 | 2 | Pup | 1 |  |
| 4 | RS2 | 7 | 2 | 2 | Pup | 1 |  |
| 4 | RS2 | 7 | 2 | 1 | Pup | 1 |  |
| 4 | RS2 | 7 | 2 | 1 | Pup | 1 |  |
| 4 | RS2 | 7 | 2 | 2 | Pup |  | 1 |
| 4 | RS2 | 7 | 2 | 2 | Pup |  | 1 |
| 4 | RS2 | 7 | 2 | 3 | Pup | 1 |  |
| 4 | RS2 | 7 | 2 | 3 | Pup | 1 |  |
| 4 | RS2 | 7 | 2 | 2 | Pup | 1 |  |
| 4 | RS3 | 7 | 2 | 6 | MO | 1 |  |
| 4 | RS3 | 7 | 2 | 5 | MO | 1 |  |
| 4 | RS3 | 7 | 2 | 0.5 | Pup | 1 |  |
| 4 | RS3 | 7 | 2 | 1 | Pup | 1 |  |
| 4 | RS3 | 7 | 2 | 1 | Pup | 1 |  |
| 4 | RS3 | 7 | 2 | 3 | MO | 1 |  |
| 4 | RS3 | 7 | 2 | 3 | MO | 1 |  |
| 4 | RS3 | 7 | 2 | 2 | Pup | 1 |  |
| 4 | RS3 | 7 | 2 | 2 | Pup | 1 |  |
| 4 | RS3 | 7 | 2 | 4 | Pup | 1 |  |
| 4 | RS3 | 7 | 2 | 5 | Pup | 1 |  |
| 4 | RS3 | 7 | 2 | 3 | Pup | 1 |  |
| 4 | RS3 | 7 | 2 | 1 | Pup | 1 |  |
| 4 | RS3 | 7 | 2 | 1 | Pup | 1 |  |
| 5 | BRN | 7 | 2 | 1 | Pup | 1 |  |
| 5 | BRN | 7 | 2 | 0.5 | Pup | 1 |  |
| 5 | BRN | 7 | 2 | 0.5 | Pup | 1 |  |
| 5 | BRN | 7 | 2 | 1 | Pup | 1 |  |
| 5 | BRN | 7 | 2 | 1 | Pup | 1 |  |
| 5 | BRN | 7 | 2 | 1 | Pup | 1 |  |
| 5 | BRN | 7 | 2 | 0.5 | Pup | 1 |  |
| 5 | BRN | 7 | 2 | 0.5 | Pup | 1 |  |
| 5 | BRN | 7 | 2 | 0.5 | Pup | 1 |  |
| 5 | BRN | 7 | 2 | 1 | Pup | 1 |  |
| 5 | BRN | 7 | 2 | 1 | Pup | 1 |  |
| 5 | BRN | 7 | 2 | 0.5 | Pup | 1 |  |
| 5 | BRN | 7 | 2 | 1 | Pup | 1 |  |
| 5 | BRN | 7 | 2 | 1 | Pup | 1 |  |
| 5 | BRN | 7 | 2 | 1 | Pup | 1 |  |
| 5 | BRN | 7 | 2 | 1 | Pup | 1 |  |
| 5 | BRN | 7 | 2 | 1 | Pup | 1 |  |
| 5 | BRN | 7 | 2 | 0.5 | Pup | 1 |  |
| 5 | BRN | 7 | 2 | 4 | Pup | 1 |  |
| 5 | BRN | 7 | 2 | 1 | Pup | 1 |  |
| 5 | BRN | 7 | 2 | 5 | Pup | 1 |  |
| 5 | BRN | 7 | 2 | 1 | Pup | 1 |  |
| 5 | BRN | 7 | 2 | 1 | Pup | 1 |  |
| 5 | BRN | 7 | 2 | 1 | Pup | 1 |  |
| 5 | BRN | 7 | 2 | 5 | Pup | 1 |  |
| 5 | BRN | 7 | 2 | 4 | Pup | 1 |  |
| 5 | BRN | 7 | 2 | 8 | Pup | 1 |  |
| 5 | BRN | 7 | 2 | 5 | Pup | 1 |  |
| 5 | BRN | 7 | 2 | 5 | Pup | 1 |  |
| 5 | BRN | 7 | 2 | 1 | Pup |  | 1 |
| 5 | BRN | 7 | 2 | 0.5 | Pup |  | 1 |
| 5 | BRN | 7 | 2 | 2 | Pup |  | 1 |
| 5 | BRN | 7 | 2 | 3 | Pup | 1 |  |
| 5 | BRN | 7 | 2 | 2 | Pup | 1 |  |
| 5 | BRN | 7 | 2 | 1 | Pup | 1 |  |
| 5 | BRN | 7 | 2 | 1 | Pup | 1 |  |
| 5 | BRN | 7 | 2 | 0.5 | Pup | 1 |  |
| 5 | BRN | 7 | 2 | 3 | Pup | 1 |  |
| 5 | BRN | 7 | 2 | 2 | Pup | 1 |  |
| 5 | BRN | 7 | 2 | 5 | Pup | 1 |  |
| 5 | BRN | 7 | 2 | 3 | Pup | 1 |  |
| 5 | BRN | 7 | 2 | 3 | Pup | 1 |  |
| 5 | BRN | 7 | 2 | 3 | Pup | 1 |  |
| 5 | BRN | 7 | 2 | 0.5 | Pup | 1 |  |
| 5 | BRN | 7 | 2 | 1 | Pup |  | 1 |
| 5 | BRN | 7 | 2 | 1 | Pup |  | 1 |
| 5 | BRN | 7 | 2 | 0.5 | Pup |  | 1 |
| 5 | BRN | 7 | 2 | 0.5 | Pup |  | 1 |
| 5 | BRN | 7 | 2 | 0.5 | Pup |  | 1 |
| 5 | BRN | 7 | 2 | 2 | Pup | 1 |  |
| 5 | BRN | 7 | 2 | 1 | Pup | 1 |  |
| 5 | BRN | 7 | 2 | 1 | Pup | 1 |  |
| 5 | BRN | 7 | 2 | 1 | Pup | 1 |  |
| 5 | BRN | 7 | 2 | 1 | Pup | 1 |  |
| 5 | BRN | 7 | 2 | 1 | Pup |  | 1 |
| 5 | BRN | 7 | 2 | 1 | Pup |  | 1 |
| 5 | BRN | 7 | 2 | 1 | Pup |  | 1 |
| 5 | KTI | 7 | 2 | 1 | Pup | 1 |  |
| 5 | KTI | 7 | 2 | 2 | Pup |  | 1 |
| 5 | KTI | 7 | 2 | 2 | Pup | 1 |  |
| 5 | KTI | 7 | 2 | 3 | Pup | 1 |  |
| 5 | KTI | 7 | 2 | 3 | Pup | 1 |  |
| 5 | KTI | 7 | 2 | 1 | Pup | 1 |  |
| 5 | KTI | 7 | 2 | 1 | Pup | 1 |  |
| 5 | KTI | 7 | 2 | 2 | Pup |  | 1 |
| 5 | KTI | 7 | 2 | 2 | Pup |  | 1 |
| 5 | KTI | 7 | 2 | 2 | Pup |  | 1 |
| 5 | KTI | 7 | 2 | 2 | Pup |  | 1 |
| 5 | KTI | 7 | 2 | 1 | Pup | 1 |  |
| 5 | KTI | 7 | 2 | 1 | Pup | 1 |  |
| 5 | KTI | 7 | 2 | 3 | Pup |  | 1 |
| 5 | KTI | 7 | 2 | 3 | Pup |  | 1 |
| 5 | KTI | 7 | 2 | 2 | Pup |  | 1 |
| 5 | KTI | 7 | 2 | 4 | Pup |  | 1 |
| 5 | KTI | 7 | 2 | 4 | Pup |  | 1 |
| 5 | KTI | 7 | 2 | 3 | Pup |  | 1 |
| 5 | KTI | 7 | 2 | 2 | Pup | 1 |  |
| 5 | KTI | 7 | 2 | 2 | Pup | 1 |  |
| 5 | KTI | 7 | 2 | 2 | Pup | 1 |  |
| 5 | KTI | 7 | 2 | 1 | Pup | 1 |  |
| 5 | KTI | 7 | 2 | 1 | Pup | 1 |  |
| 5 | BBR | 7 | 4 | 1 | Pup | 1 |  |
| 5 | BBR | 7 | 4 | 0.5 | Pup | 1 |  |
| 5 | BBR | 7 | 4 | 2 | Pup | 1 |  |
| 5 | BBR | 7 | 4 | 2 | Pup | 1 |  |
| 5 | BBR | 7 | 4 | 2 | Pup | 1 |  |
| 5 | BBR | 7 | 4 | 2 | Pup | 1 |  |
| 5 | BBR | 7 | 4 | 2 | Pup | 1 |  |
| 5 | BBR | 7 | 4 | 0.5 | Pup |  | 1 |
| 5 | BBR | 7 | 4 | 0.5 | Pup |  | 1 |
| 5 | BBR | 7 | 4 | 0.5 | Pup |  | 1 |
| 5 | BBR | 7 | 4 | 1 | Pup | 1 |  |
| 5 | BBR | 7 | 4 | 1 | Pup |  | 1 |
| 5 | BBR | 7 | 4 | 1 | Pup |  | 1 |
| 5 | BBR | 7 | 4 | 4 | Pup | 1 |  |
| 5 | BBR | 7 | 4 | 4 | Pup | 1 |  |
| 5 | BBR | 7 | 4 | 4 | Pup | 1 |  |
| 5 | BBR | 7 | 4 | 4 | Pup | 1 |  |
| 5 | BBR | 7 | 4 | 1 | Pup |  | 1 |
| 5 | BBR | 7 | 4 | 1 | Pup |  | 1 |
| 5 | BBR | 7 | 4 | 1 | Pup |  | 1 |
| 5 | BBR | 7 | 4 | 1 | Pup | 1 |  |
| 5 | BBR | 7 | 4 | 1 | Pup | 1 |  |
| 5 | BBR | 7 | 4 | 1 | Pup | 1 |  |
| 5 | BBR | 7 | 4 | 1 | Pup | 1 |  |
| 5 | BBR | 7 | 4 | 1 | Pup | 1 |  |
| 5 | BBR | 7 | 4 | 1 | Pup | 1 |  |
| 5 | BBR | 7 | 4 | 0.5 | Pup |  | 1 |
| 5 | BBR | 7 | 4 | 0.5 | Pup |  | 1 |
| 5 | BBR | 7 | 4 | 1 | Pup |  | 1 |
| 5 | BBR | 7 | 4 | 1 | Pup |  | 1 |
| 5 | BBR | 7 | 4 | 1 | Pup |  | 1 |
| 5 | BBR | 7 | 4 | 1 | Pup |  | 1 |
| 5 | BBR | 7 | 4 | 1 | Pup |  | 1 |
| 5 | BBR | 7 | 4 | 0.5 | Pup |  | 1 |
| 5 | BBR | 7 | 4 | 0.5 | Pup |  | 1 |
| 5 | BBR | 7 | 4 | 0.5 | Pup |  | 1 |
| 5 | BBR | 7 | 4 | 2 | Pup |  | 1 |
| 5 | BBR | 7 | 4 | 2 | Pup |  | 1 |
| 5 | BBR | 7 | 4 | 2 | Pup |  | 1 |
| 5 | BBR | 7 | 4 | 3 | Pup |  | 1 |
| 5 | BBR | 7 | 4 | 3 | Pup |  | 1 |
| 5 | BBR | 7 | 4 | 1 | Pup |  | 1 |
| 5 | BBR | 7 | 4 | 2 | Pup |  | 1 |
| 5 | BBR | 7 | 4 | 2 | Pup |  | 1 |
| 5 | BBR | 7 | 4 | 2 | Pup |  | 1 |
| 5 | BBR | 7 | 4 | 2 | Pup |  | 1 |
| 5 | BBR | 7 | 4 | 2 | Pup |  | 1 |
| 5 | BBR | 7 | 4 | 1 | Pup |  | 1 |
| 5 | BBR | 7 | 4 | 1 | Pup |  | 1 |
| 5 | BBR | 7 | 4 | 1 | Pup |  | 1 |
| 5 | BBR | 7 | 4 | 1 | Pup |  | 1 |
| 5 | BBR | 7 | 4 | 1 | Pup |  | 1 |
| 5 | BBR | 7 | 4 | 2 | Pup | 1 |  |
| 5 | BBR | 7 | 4 | 1 | Pup | 1 |  |
| 5 | BBR | 7 | 4 | 0.5 | Pup | 1 |  |
| 5 | BBR | 7 | 4 | 1 | Pup |  | 1 |
| 5 | BBR | 7 | 4 | 1 | Pup |  | 1 |
| 5 | BBR | 7 | 4 | 1 | Pup |  | 1 |
| 5 | BBR | 7 | 4 | 0.5 | Pup |  | 1 |
| 5 | BBR | 7 | 4 | 0.5 | Pup |  | 1 |
| 5 | BBR | 7 | 4 | 0.5 | Pup |  | 1 |
| 5 | BBR | 7 | 4 | 1 | Pup | 1 |  |
| 5 | BBR | 7 | 4 | 1 | Pup | 1 |  |
| 5 | BBR | 7 | 4 | 1 | Pup | 1 |  |
| 5 | BBR | 7 | 4 | 1 | Pup | 1 |  |
| 5 | BBR | 7 | 4 | 1 | Pup | 1 |  |
| 5 | BBR | 7 | 4 | 2 | Pup | 1 |  |
| 5 | BBR | 7 | 4 | 2 | Pup | 1 |  |
| 5 | BBR | 7 | 4 | 2 | Pup | 1 |  |
| 5 | BBR | 7 | 4 | 2 | Pup | 1 |  |
| 5 | BBR | 7 | 4 | 2 | Pup | 1 |  |
| 5 | BBR | 7 | 4 | 1 | Pup |  | 1 |
| 5 | BBR | 7 | 4 | 1 | Pup |  | 1 |
| 5 | BBR | 7 | 4 | 1 | Pup |  | 1 |
| 5 | BBR | 7 | 4 | 1 | Pup |  | 1 |
| 5 | BBR | 7 | 4 | 0.5 | Pup | 1 |  |
| 5 | BBR | 7 | 4 | 0.5 | Pup | 1 |  |
| 5 | BBR | 7 | 4 | 0.5 | Pup | 1 |  |
| 5 | BBR | 7 | 4 | 1 | Pup | 1 |  |
| 5 | BBR | 7 | 4 | 1 | Pup | 1 |  |
| 5 | BBR | 7 | 4 | 0.5 | Pup | 1 |  |
| 5 | BBR | 7 | 4 | 0.5 | Pup | 1 |  |
| 5 | BBR | 7 | 4 | 0.5 | Pup | 1 |  |
| 5 | BBR | 7 | 4 | 1 | Pup | 1 |  |
| 5 | BBR | 7 | 4 | 1 | Pup | 1 |  |
| 5 | BBR | 7 | 4 | 0.5 | Pup | 1 |  |
| 5 | BBR | 7 | 4 | 0.5 | Pup | 1 |  |
| 5 | BBR | 7 | 4 | 0.5 | Pup | 1 |  |
| 5 | BBR | 7 | 4 | 0.5 | Pup | 1 |  |
| 5 | BBR | 7 | 4 | 0.5 | Pup | 1 |  |
| 5 | BBR | 7 | 4 | 0.5 | Pup | 1 |  |
| 5 | BBR | 7 | 4 | 0.5 | Pup | 1 |  |
| 5 | WHI | 7 | 2 | 2 | MO | 1 |  |
| 5 | WHI | 7 | 2 | 2 | MO | 1 |  |
| 5 | WHI | 7 | 2 | 6 | Pup | 1 |  |
| 5 | WHI | 7 | 2 | 3 | Pup | 1 |  |
| 5 | WHI | 7 | 2 | 1 | Pup | 1 |  |
| 5 | WHI | 7 | 2 | 0.5 | Pup | 1 |  |
| 5 | WHI | 7 | 2 | 0.5 | Pup | 1 |  |
| 5 | WHI | 7 | 2 | 4 | Pup | 1 |  |
| 5 | WHI | 7 | 2 | 4 | MO | 1 |  |
| 5 | WHI | 7 | 2 | 4 | MO | 1 |  |
| 5 | WHI | 7 | 2 | 3 | Pup | 1 |  |
| 5 | WHI | 7 | 2 | 2 | Pup | 1 |  |
| 5 | WHI | 7 | 2 | 4 | MO | 1 |  |
| 5 | WHI | 7 | 2 | 4 | MO | 1 |  |
| 5 | WHI | 7 | 2 | 1 | Pup | 1 |  |
| 5 | WHI | 7 | 2 | 1 | Pup | 1 |  |
| 5 | WHI | 7 | 2 | 4 | Pup | 1 |  |
| 5 | WHI | 7 | 2 | 3 | Pup | 1 |  |
| 5 | WHI | 7 | 2 | 1 | Pup | 1 |  |
| 5 | WHI | 7 | 2 | 2 | Pup |  | 1 |
| 5 | WHI | 7 | 2 | 4 | Pup |  | 1 |
| 5 | WHI | 7 | 2 | 4 | Pup |  | 1 |
| 5 | WHI | 7 | 2 | 2 | Pup | 1 |  |
| 5 | WHI | 7 | 2 | 2 | Pup | 1 |  |
| 5 | WHI | 7 | 2 | 3 | MO | 1 |  |
| 5 | WHI | 7 | 2 | 3 | MO | 1 |  |
| 5 | WHI | 7 | 2 | 1 | Pup | 1 |  |
| 5 | WHI | 7 | 2 | 1 | Pup | 1 |  |
| 5 | RS5 | 7 | 3 | 3 | Pup | 1 |  |
| 5 | RS5 | 7 | 3 | 3 | Pup | 1 |  |
| 5 | RS5 | 7 | 3 | 3 | Pup | 1 |  |
| 5 | RS5 | 7 | 3 | 4 | MO | 1 |  |
| 5 | RS5 | 7 | 3 | 4 | MO | 1 |  |
| 5 | RS5 | 7 | 3 | 4 | MO | 1 |  |
| 5 | RS5 | 7 | 3 | 2 | Pup | 1 |  |
| 5 | RS5 | 7 | 3 | 2 | Pup | 1 |  |
| 5 | RS5 | 7 | 3 | 2 | Pup | 1 |  |
| 5 | RS5 | 7 | 3 | 3 | Pup |  | 1 |
| 5 | RS5 | 7 | 3 | 1 | Pup | 1 |  |
| 5 | RS5 | 7 | 3 | 1 | Pup | 1 |  |
| 5 | RS5 | 7 | 3 | 1 | Pup | 1 |  |
| 5 | RS5 | 7 | 3 | 5 | Pup | 1 |  |
| 5 | RS5 | 7 | 3 | 5 | Pup | 1 |  |
| 5 | RS5 | 7 | 3 | 5 | Pup | 1 |  |
| 4 | CAN | 8 | 5 | 0.5 | Pup | 1 |  |
| 4 | CAN | 8 | 5 | 0.5 | Pup | 1 |  |
| 4 | CAN | 8 | 5 | 0.5 | Pup | 1 |  |
| 4 | CAN | 8 | 5 | 0.5 | Pup | 1 |  |
| 4 | CAN | 8 | 5 | 0.5 | Pup | 1 |  |
| 4 | CAN | 8 | 5 | 0.5 | Pup | 1 |  |
| 4 | CAN | 8 | 5 | 0.5 | Pup | 1 |  |
| 4 | CAN | 8 | 5 | 0.5 | Pup | 1 |  |
| 4 | CAN | 8 | 5 | 7 | Pup | 1 |  |
| 4 | CAN | 8 | 5 | 7 | Pup | 1 |  |
| 4 | CAN | 8 | 5 | 7 | Pup | 1 |  |
| 4 | CAN | 8 | 5 | 7 | Pup | 1 |  |
| 4 | CAN | 8 | 5 | 7 | Pup | 1 |  |
| 4 | CAN | 8 | 5 | 0.5 | Pup | 1 |  |
| 4 | CAN | 8 | 5 | 0.5 | Pup | 1 |  |
| 4 | CAN | 8 | 5 | 0.5 | Pup | 1 |  |
| 4 | CAN | 8 | 5 | 7 | MO | 1 |  |
| 4 | CAN | 8 | 5 | 7 | MO | 1 |  |
| 4 | CAN | 8 | 5 | 7 | MO | 1 |  |
| 4 | CAN | 8 | 5 | 7 | MO | 1 |  |
| 4 | CAN | 8 | 5 | 7 | MO | 1 |  |
| 4 | PF1 | 8 | 3 | 0.5 | Pup | 1 |  |
| 4 | PF1 | 8 | 3 | 2 | Pup | 1 |  |
| 4 | PF1 | 8 | 3 | 2 | Pup | 1 |  |
| 4 | PF1 | 8 | 3 | 3 | Pup | 1 |  |
| 4 | PF1 | 8 | 3 | 3 | Pup | 1 |  |
| 4 | PF1 | 8 | 3 | 2 | Pup | 1 |  |
| 4 | RS1 | 8 | 2 | 3 | Pup |  | 1 |
| 4 | RS1 | 8 | 2 | 3 | Pup |  | 1 |
| 4 | RS1 | 8 | 2 | 1 | Pup | 1 |  |
| 4 | RS1 | 8 | 2 | 2 | Pup |  | 1 |
| 4 | RS1 | 8 | 2 | 4 | Pup |  | 1 |
| 4 | RS1 | 8 | 2 | 4 | Pup |  | 1 |
| 4 | RS1 | 8 | 2 | 1 | Pup |  | 1 |
| 4 | RS1 | 8 | 2 | 0.5 | Pup |  | 1 |
| 4 | RS1 | 8 | 2 | 1 | Pup | 1 |  |
| 4 | RS1 | 8 | 2 | 1 | Pup | 1 |  |
| 4 | RS1 | 8 | 2 | 2 | Pup |  | 1 |
| 4 | RS1 | 8 | 2 | 0.5 | Pup |  | 1 |
| 4 | RS1 | 8 | 2 | 1 | Pup |  | 1 |
| 4 | RS1 | 8 | 2 | 2 | Pup | 1 |  |
| 4 | RS1 | 8 | 2 | 1 | Pup | 1 |  |
| 4 | RS1 | 8 | 2 | 2 | Pup | 1 |  |
| 4 | RS1 | 8 | 2 | 2 | Pup | 1 |  |
| 4 | RS1 | 8 | 2 | 2 | Pup | 1 |  |
| 4 | RS1 | 8 | 2 | 5 | Pup |  | 1 |
| 4 | RS1 | 8 | 2 | 4 | Pup |  | 1 |
| 4 | RS1 | 8 | 2 | 3 | Pup |  | 1 |
| 4 | RS1 | 8 | 2 | 3 | Pup |  | 1 |
| 4 | RS1 | 8 | 2 | 1 | Pup | 1 |  |
| 4 | RS1 | 8 | 2 | 1 | Pup | 1 |  |
| 4 | RS1 | 8 | 2 | 0.5 | Pup |  | 1 |
| 4 | RS1 | 8 | 2 | 0.5 | Pup |  | 1 |
| 4 | RS1 | 8 | 2 | 1 | Pup | 1 |  |
| 4 | RS1 | 8 | 2 | 0.5 | Pup | 1 |  |
| 4 | RS1 | 8 | 2 | 0.5 | Pup | 1 |  |
| 4 | RS1 | 8 | 2 | 1 | Pup |  | 1 |
| 4 | RS1 | 8 | 2 | 1 | Pup |  | 1 |
| 4 | RS1 | 8 | 2 | 1 | Pup |  | 1 |
| 4 | RS1 | 8 | 2 | 0.5 | Pup |  | 1 |
| 4 | RS1 | 8 | 2 | 1 | Pup |  | 1 |
| 4 | RS1 | 8 | 2 | 0.5 | Pup |  | 1 |
| 4 | RS3 | 8 | 2 | 1 | Pup | 1 |  |
| 4 | RS3 | 8 | 2 | 4 | Pup | 1 |  |
| 4 | RS3 | 8 | 2 | 1 | Pup | 1 |  |
| 4 | RS3 | 8 | 2 | 5 | Pup |  | 1 |
| 4 | RS3 | 8 | 2 | 5 | Pup |  | 1 |
| 4 | RS3 | 8 | 2 | 1 | Pup |  | 1 |
| 4 | RS3 | 8 | 2 | 1 | Pup |  | 1 |
| 4 | RS3 | 8 | 2 | 1 | Pup | 1 |  |
| 4 | RS3 | 8 | 2 | 1 | Pup | 1 |  |
| 4 | RS3 | 8 | 2 | 0.5 | Pup | 1 |  |
| 4 | RS3 | 8 | 2 | 0.5 | Pup | 1 |  |
| 4 | RS3 | 8 | 2 | 1 | Pup | 1 |  |
| 4 | RS3 | 8 | 2 | 1 | Pup | 1 |  |
| 4 | RS3 | 8 | 2 | 0.5 | Pup | 1 |  |
| 4 | RS3 | 8 | 2 | 0.5 | Pup | 1 |  |
| 4 | RS3 | 8 | 2 | 4 | Pup | 1 |  |
| 4 | RS3 | 8 | 2 | 2 | Pup | 1 |  |
| 5 | BRN | 8 | 2 | 2 | Pup |  | 1 |
| 5 | BRN | 8 | 2 | 2 | Pup |  | 1 |
| 5 | BRN | 8 | 2 | 0.5 | Pup |  | 1 |
| 5 | BRN | 8 | 2 | 0.5 | Pup |  | 1 |
| 5 | BRN | 8 | 2 | 1 | Pup |  | 1 |
| 5 | BRN | 8 | 2 | 1 | Pup |  | 1 |
| 5 | BRN | 8 | 2 | 0.5 | Pup |  | 1 |
| 5 | BRN | 8 | 2 | 1 | Pup |  | 1 |
| 5 | BRN | 8 | 2 | 1 | Pup |  | 1 |
| 5 | BRN | 8 | 2 | 1 | Pup |  | 1 |
| 5 | BRN | 8 | 2 | 3 | Pup |  | 1 |
| 5 | BRN | 8 | 2 | 2 | Pup |  | 1 |
| 5 | BRN | 8 | 2 | 1 | Pup |  | 1 |
| 5 | BRN | 8 | 2 | 1 | Pup |  | 1 |
| 5 | BRN | 8 | 2 | 1 | Pup | 1 |  |
| 5 | BRN | 8 | 2 | 1 | Pup | 1 |  |
| 5 | BRN | 8 | 2 | 1 | Pup |  | 1 |
| 5 | BRN | 8 | 2 | 1 | Pup |  | 1 |
| 5 | BRN | 8 | 2 | 1 | Pup |  | 1 |
| 5 | BRN | 8 | 2 | 1 | Pup |  | 1 |
| 5 | BRN | 8 | 2 | 0.5 | Pup | 1 |  |
| 5 | BRN | 8 | 2 | 0.5 | Pup | 1 |  |
| 5 | KTI | 8 | 2 | 1 | Pup |  | 1 |
| 5 | KTI | 8 | 2 | 0.5 | Pup |  | 1 |
| 5 | KTI | 8 | 2 | 1 | Pup | 1 |  |
| 5 | KTI | 8 | 2 | 1 | Pup | 1 |  |
| 5 | KTI | 8 | 2 | 0.5 | Pup | 1 |  |
| 5 | KTI | 8 | 2 | 0.5 | Pup | 1 |  |
| 5 | KTI | 8 | 2 | 0.5 | Pup |  | 1 |
| 5 | KTI | 8 | 2 | 6 | Pup | 1 |  |
| 5 | KTI | 8 | 2 | 6 | Pup | 1 |  |
| 5 | KTI | 8 | 2 | 0.5 | Pup | 1 |  |
| 5 | KTI | 8 | 2 | 0.5 | Pup | 1 |  |
| 5 | KTI | 8 | 2 | 1 | Pup |  | 1 |
| 5 | KTI | 8 | 2 | 1 | Pup |  | 1 |
| 5 | KTI | 8 | 2 | 1 | Pup |  | 1 |
| 5 | KTI | 8 | 2 | 0.5 | Pup |  | 1 |
| 5 | BBR | 8 | 4 | 3 | Pup |  | 1 |
| 5 | BBR | 8 | 4 | 3 | Pup |  | 1 |
| 5 | BBR | 8 | 4 | 2 | Pup |  | 1 |
| 5 | BBR | 8 | 4 | 2 | Pup | 1 |  |
| 5 | BBR | 8 | 4 | 2 | Pup | 1 |  |
| 5 | BBR | 8 | 4 | 2 | Pup | 1 |  |
| 5 | BBR | 8 | 4 | 2 | Pup | 1 |  |
| 5 | BBR | 8 | 4 | 1 | Pup | 1 |  |
| 5 | BBR | 8 | 4 | 4 | Pup | 1 |  |
| 5 | BBR | 8 | 4 | 4 | Pup | 1 |  |
| 5 | BBR | 8 | 4 | 4 | Pup | 1 |  |
| 5 | BBR | 8 | 4 | 4 | Pup | 1 |  |
| 5 | BBR | 8 | 4 | 4 | Pup | 1 |  |
| 5 | BBR | 8 | 4 | 4 | Pup |  | 1 |
| 5 | BBR | 8 | 4 | 4 | Pup |  | 1 |
| 5 | BBR | 8 | 4 | 4 | Pup | 1 |  |
| 5 | BBR | 8 | 4 | 4 | Pup | 1 |  |
| 5 | BBR | 8 | 4 | 4 | Pup | 1 |  |
| 5 | BBR | 8 | 4 | 5 | Pup | 1 |  |
| 5 | BBR | 8 | 4 | 5 | Pup | 1 |  |
| 5 | BBR | 8 | 4 | 5 | Pup | 1 |  |
| 5 | BBR | 8 | 4 | 5 | Pup | 1 |  |
| 5 | BBR | 8 | 4 | 5 | Pup | 1 |  |
| 5 | BBR | 8 | 4 | 4 | Pup | 1 |  |
| 5 | BBR | 8 | 4 | 3 | Pup | 1 |  |
| 5 | BBR | 8 | 4 | 3 | Pup | 1 |  |
| 5 | BBR | 8 | 4 | 1 | Pup | 1 |  |
| 5 | BBR | 8 | 4 | 1 | Pup | 1 |  |
| 5 | BBR | 8 | 4 | 2 | Pup | 1 |  |
| 5 | BBR | 8 | 4 | 2 | Pup |  | 1 |
| 5 | BBR | 8 | 4 | 2 | Pup |  | 1 |
| 5 | BBR | 8 | 4 | 1 | Pup | 1 |  |
| 5 | BBR | 8 | 4 | 1 | Pup | 1 |  |
| 5 | BBR | 8 | 4 | 1 | Pup | 1 |  |
| 5 | BBR | 8 | 4 | 1 | Pup | 1 |  |
| 5 | BBR | 8 | 4 | 0.5 | Pup | 1 |  |
| 5 | BBR | 8 | 4 | 2 | Pup | 1 |  |
| 5 | BBR | 8 | 4 | 2 | Pup | 1 |  |
| 5 | BBR | 8 | 4 | 2 | Pup | 1 |  |
| 5 | BBR | 8 | 4 | 2 | Pup | 1 |  |
| 5 | BBR | 8 | 4 | 1 | Pup | 1 |  |
| 5 | BBR | 8 | 4 | 1 | Pup | 1 |  |
| 5 | BBR | 8 | 4 | 1 | Pup |  | 1 |
| 5 | BBR | 8 | 4 | 1 | Pup |  | 1 |
| 5 | BBR | 8 | 4 | 1 | Pup | 1 |  |
| 5 | BBR | 8 | 4 | 1 | Pup | 1 |  |
| 5 | BBR | 8 | 4 | 1 | Pup | 1 |  |
| 5 | BBR | 8 | 4 | 1 | Pup | 1 |  |
| 5 | BBR | 8 | 4 | 2 | Pup | 1 |  |
| 5 | BBR | 8 | 4 | 2 | Pup | 1 |  |
| 5 | BBR | 8 | 4 | 2 | Pup | 1 |  |
| 5 | BBR | 8 | 4 | 2 | Pup | 1 |  |
| 5 | BBR | 8 | 4 | 3 | Pup | 1 |  |
| 5 | BBR | 8 | 4 | 3 | Pup | 1 |  |
| 5 | BBR | 8 | 4 | 3 | Pup | 1 |  |
| 5 | BBR | 8 | 4 | 3 | Pup | 1 |  |
| 5 | WHI | 8 | 2 | 1 | Pup |  | 1 |
| 5 | WHI | 8 | 2 | 1 | Pup |  | 1 |
| 5 | WHI | 8 | 2 | 2 | Pup |  | 1 |
| 5 | WHI | 8 | 2 | 1 | Pup | 1 |  |
| 5 | WHI | 8 | 2 | 2 | Pup | 1 |  |
| 5 | WHI | 8 | 2 | 2 | Pup | 1 |  |
| 5 | WHI | 8 | 2 | 2 | Pup | 1 |  |
| 5 | WHI | 8 | 2 | 2 | Pup | 1 |  |
| 5 | WHI | 8 | 2 | 1 | Pup | 1 |  |
| 5 | WHI | 8 | 2 | 4 | Pup |  | 1 |
| 5 | WHI | 8 | 2 | 2 | Pup |  | 1 |
| 5 | WHI | 8 | 2 | 0.5 | Pup | 1 |  |
| 5 | WHI | 8 | 2 | 2 | Pup | 1 |  |
| 5 | WHI | 8 | 2 | 2 | Pup | 1 |  |
| 5 | WHI | 8 | 2 | 2 | Pup | 1 |  |
| 5 | WHI | 8 | 2 | 2 | Pup | 1 |  |
| 5 | WHI | 8 | 2 | 1 | Pup | 1 |  |
| 5 | WHI | 8 | 2 | 1 | Pup | 1 |  |
| 5 | WHI | 8 | 2 | 2 | Pup | 1 |  |
| 5 | WHI | 8 | 2 | 2 | Pup | 1 |  |
| 5 | WHI | 8 | 2 | 3 | Pup |  | 1 |
| 5 | WHI | 8 | 2 | 3 | Pup |  | 1 |
| 5 | WHI | 8 | 2 | 2 | Pup |  | 1 |
| 5 | WHI | 8 | 2 | 2 | Pup |  | 1 |
| 5 | WHI | 8 | 2 | 2 | Pup |  | 1 |
| 5 | WHI | 8 | 2 | 0.5 | Pup |  | 1 |
| 5 | WHI | 8 | 2 | 0.5 | Pup |  | 1 |
| 5 | WHI | 8 | 2 | 1 | Pup |  | 1 |
| 5 | WHI | 8 | 2 | 1 | Pup |  | 1 |
| 5 | WHI | 8 | 2 | 1 | Pup |  | 1 |
| 5 | WHI | 8 | 2 | 1 | Pup |  | 1 |
| 5 | WHI | 8 | 2 | 4 | Pup | 1 |  |
| 5 | WHI | 8 | 2 | 1 | Pup | 1 |  |
| 5 | WHI | 8 | 2 | 0.5 | Pup | 1 |  |
| 5 | WHI | 8 | 2 | 3 | Pup | 1 |  |
| 5 | WHI | 8 | 2 | 3 | Pup | 1 |  |
| 5 | WHI | 8 | 2 | 1 | Pup | 1 |  |
| 5 | WHI | 8 | 2 | 1 | Pup | 1 |  |
| 5 | WHI | 8 | 2 | 2 | Pup | 1 |  |
| 5 | WHI | 8 | 2 | 2 | Pup | 1 |  |
| 5 | WHI | 8 | 2 | 2 | Pup | 1 |  |
| 5 | WHI | 8 | 2 | 0.5 | Pup | 1 |  |
| 5 | WHI | 8 | 2 | 0.5 | Pup | 1 |  |
| 5 | WHI | 8 | 2 | 2 | Pup | 1 |  |
| 5 | WHI | 8 | 2 | 1 | Pup | 1 |  |
| 4 | PF1 | 9 | 2 | 1 | Pup | 1 |  |
| 4 | PF1 | 9 | 2 | 1 | Pup |  | 1 |
| 4 | PF1 | 9 | 2 | 1 | Pup |  | 1 |
| 4 | RS1 | 9 | 2 | 5 | Pup | 1 |  |
| 4 | RS1 | 9 | 2 | 2 | Pup | 1 |  |
| 4 | RS1 | 9 | 2 | 2 | Pup | 1 |  |
| 4 | RS1 | 9 | 2 | 2 | Pup | 1 |  |
| 4 | RS1 | 9 | 2 | 1 | Pup |  | 1 |
| 4 | RS1 | 9 | 2 | 1 | Pup |  | 1 |
| 4 | RS1 | 9 | 2 | 1 | Pup |  | 1 |
| 4 | RS1 | 9 | 2 | 1 | Pup |  | 1 |
| 4 | RS1 | 9 | 2 | 1 | Pup |  | 1 |
| 4 | RS1 | 9 | 2 | 0.5 | Pup |  | 1 |
| 4 | RS1 | 9 | 2 | 1 | Pup |  | 1 |
| 4 | RS1 | 9 | 2 | 1 | Pup |  | 1 |
| 4 | RS1 | 9 | 2 | 1 | Pup |  | 1 |
| 4 | RS1 | 9 | 2 | 2 | Pup |  | 1 |
| 4 | RS1 | 9 | 2 | 1 | Pup |  | 1 |
| 4 | RS1 | 9 | 2 | 1 | Pup |  | 1 |
| 4 | RS1 | 9 | 2 | 1 | Pup |  | 1 |
| 4 | RS1 | 9 | 2 | 3 | Pup |  | 1 |
| 4 | RS1 | 9 | 2 | 3 | Pup |  | 1 |
| 4 | RS1 | 9 | 2 | 3 | Pup |  | 1 |
| 4 | RS1 | 9 | 2 | 1 | Pup |  | 1 |
| 4 | RS1 | 9 | 2 | 0.5 | Pup |  | 1 |
| 4 | RS1 | 9 | 2 | 0.5 | Pup |  | 1 |
| 4 | RS1 | 9 | 2 | 0.5 | Pup |  | 1 |
| 4 | RS1 | 9 | 2 | 4 | Pup |  | 1 |
| 4 | RS1 | 9 | 2 | 1 | Pup |  | 1 |
| 4 | RS1 | 9 | 2 | 0.5 | Pup |  | 1 |
| 4 | RS1 | 9 | 2 | 1 | Pup |  | 1 |
| 4 | RS1 | 9 | 2 | 1 | Pup |  | 1 |
| 4 | RS1 | 9 | 2 | 1 | Pup |  | 1 |
| 4 | RS1 | 9 | 2 | 1 | Pup | 1 |  |
| 4 | RS1 | 9 | 2 | 2 | Pup |  | 1 |
| 4 | RS1 | 9 | 2 | 0.5 | Pup |  | 1 |
| 4 | RS2 | 9 | 2 | 0.5 | Pup | 1 |  |
| 4 | RS2 | 9 | 2 | 0.5 | Pup | 1 |  |
| 4 | RS2 | 9 | 2 | 0.5 | Pup | 1 |  |
| 4 | RS2 | 9 | 2 | 1 | Pup | 1 |  |
| 4 | RS2 | 9 | 2 | 0.5 | Pup | 1 |  |
| 4 | RS2 | 9 | 2 | 1 | Pup | 1 |  |
| 4 | RS2 | 9 | 2 | 0.5 | Pup | 1 |  |
| 4 | RS2 | 9 | 2 | 0.5 | Pup | 1 |  |
| 4 | RS2 | 9 | 2 | 2 | Pup | 1 |  |
| 4 | RS2 | 9 | 2 | 0.5 | Pup | 1 |  |
| 4 | RS2 | 9 | 2 | 1 | Pup | 1 |  |
| 4 | RS2 | 9 | 2 | 1 | Pup | 1 |  |
| 4 | RS2 | 9 | 2 | 4 | Pup | 1 |  |
| 4 | RS2 | 9 | 2 | 1 | Pup | 1 |  |
| 4 | RS2 | 9 | 2 | 2 | Pup | 1 |  |
| 4 | RS2 | 9 | 2 | 0.5 | Pup | 1 |  |
| 4 | RS2 | 9 | 2 | 1 | Pup | 1 |  |
| 4 | RS2 | 9 | 2 | 2 | Pup | 1 |  |
| 4 | RS2 | 9 | 2 | 1 | Pup | 1 |  |
| 4 | RS2 | 9 | 2 | 3 | Pup | 1 |  |
| 4 | RS2 | 9 | 2 | 1 | Pup | 1 |  |
| 4 | RS2 | 9 | 2 | 0.5 | Pup | 1 |  |
| 4 | RS2 | 9 | 2 | 1 | Pup | 1 |  |
| 4 | RS2 | 9 | 2 | 3 | Pup | 1 |  |
| 4 | RS2 | 9 | 2 | 3 | Pup | 1 |  |
| 4 | RS2 | 9 | 2 | 3 | Pup | 1 |  |
| 4 | RS2 | 9 | 2 | 0.5 | Pup | 1 |  |
| 4 | RS2 | 9 | 2 | 1 | Pup |  | 1 |
| 4 | RS2 | 9 | 2 | 2 | Pup | 1 |  |
| 4 | RS2 | 9 | 2 | 2 | Pup | 1 |  |
| 4 | RS2 | 9 | 2 | 1 | Pup | 1 |  |
| 4 | RS2 | 9 | 2 | 2 | Pup | 1 |  |
| 4 | RS2 | 9 | 2 | 2 | Pup | 1 |  |
| 4 | RS2 | 9 | 2 | 0.5 | Pup | 1 |  |
| 4 | RS2 | 9 | 2 | 0.5 | Pup | 1 |  |
| 4 | RS2 | 9 | 2 | 3 | Pup | 1 |  |
| 4 | RS2 | 9 | 2 | 3 | Pup | 1 |  |
| 4 | RS2 | 9 | 2 | 1 | Pup | 1 |  |
| 4 | RS2 | 9 | 2 | 0.5 | Pup | 1 |  |
| 4 | RS2 | 9 | 2 | 2 | Pup | 1 |  |
| 4 | RS2 | 9 | 2 | 2 | Pup | 1 |  |
| 4 | RS2 | 9 | 2 | 5 | Pup | 1 |  |
| 4 | RS2 | 9 | 2 | 5 | Pup | 1 |  |
| 4 | RS2 | 9 | 2 | 1 | Pup | 1 |  |
| 4 | RS2 | 9 | 2 | 1 | Pup | 1 |  |
| 4 | RS2 | 9 | 2 | 1 | Pup | 1 |  |
| 4 | RS2 | 9 | 2 | 1 | Pup | 1 |  |
| 4 | RS2 | 9 | 2 | 0.5 | Pup | 1 |  |
| 4 | RS2 | 9 | 2 | 4 | Pup | 1 |  |
| 4 | RS2 | 9 | 2 | 4 | Pup | 1 |  |
| 4 | RS2 | 9 | 2 | 1 | Pup | 1 |  |
| 4 | RS2 | 9 | 2 | 1 | Pup | 1 |  |
| 4 | RS2 | 9 | 2 | 0.5 | Pup | 1 |  |
| 4 | RS2 | 9 | 2 | 0.5 | Pup | 1 |  |
| 4 | RS2 | 9 | 2 | 2 | Pup | 1 |  |
| 4 | RS2 | 9 | 2 | 0.5 | Pup | 1 |  |
| 4 | RS2 | 9 | 2 | 1 | Pup | 1 |  |
| 4 | RS2 | 9 | 2 | 1 | Pup | 1 |  |
| 4 | RS2 | 9 | 2 | 0.5 | Pup | 1 |  |
| 4 | RS2 | 9 | 2 | 0.5 | Pup | 1 |  |
| 4 | RS2 | 9 | 2 | 1 | Pup | 1 |  |
| 4 | RS3 | 9 | 1 | 2 | Pup | 1 |  |
| 4 | RS3 | 9 | 1 | 2 | Pup | 1 |  |
| 4 | RS3 | 9 | 1 | 1 | Pup |  | 1 |
| 5 | BRN | 9 | 2 | 2 | Pup | 1 |  |
| 5 | BRN | 9 | 2 | 1 | Pup | 1 |  |
| 5 | BRN | 9 | 2 | 1 | Pup | 1 |  |
| 5 | BRN | 9 | 2 | 1 | Pup | 1 |  |
| 5 | BRN | 9 | 2 | 1 | Pup | 1 |  |
| 5 | BRN | 9 | 2 | 1 | Pup |  | 1 |
| 5 | BRN | 9 | 2 | 1 | Pup |  | 1 |
| 5 | BRN | 9 | 2 | 2 | Pup |  | 1 |
| 5 | BRN | 9 | 2 | 2 | Pup |  | 1 |
| 5 | BRN | 9 | 2 | 1 | Pup |  | 1 |
| 5 | BRN | 9 | 2 | 1 | Pup |  | 1 |
| 5 | BRN | 9 | 2 | 1 | Pup |  | 1 |
| 5 | BRN | 9 | 2 | 0.5 | Pup |  | 1 |
| 5 | BRN | 9 | 2 | 1 | Pup |  | 1 |
| 5 | BRN | 9 | 2 | 1 | Pup |  | 1 |
| 5 | BRN | 9 | 2 | 1 | Pup |  | 1 |
| 5 | BRN | 9 | 2 | 0.5 | Pup |  | 1 |
| 5 | BRN | 9 | 2 | 0.5 | Pup |  | 1 |
| 5 | BRN | 9 | 2 | 3 | Pup |  | 1 |
| 5 | BRN | 9 | 2 | 3 | Pup |  | 1 |
| 5 | BRN | 9 | 2 | 3 | Pup |  | 1 |
| 5 | BRN | 9 | 2 | 2 | Pup |  | 1 |
| 5 | BRN | 9 | 2 | 2 | Pup |  | 1 |
| 5 | BRN | 9 | 2 | 1 | Pup |  | 1 |
| 5 | BRN | 9 | 2 | 1 | Pup |  | 1 |
| 5 | BRN | 9 | 2 | 0.5 | Pup |  | 1 |
| 5 | BRN | 9 | 2 | 0.5 | Pup |  | 1 |
| 5 | BRN | 9 | 2 | 0.5 | Pup |  | 1 |
| 5 | BRN | 9 | 2 | 2 | Pup |  | 1 |
| 5 | BRN | 9 | 2 | 2 | Pup |  | 1 |
| 5 | BRN | 9 | 2 | 2 | Pup |  | 1 |
| 5 | BRN | 9 | 2 | 2 | Pup | 1 |  |
| 5 | BRN | 9 | 2 | 1 | Pup | 1 |  |
| 5 | BRN | 9 | 2 | 3 | Pup |  | 1 |
| 5 | BRN | 9 | 2 | 3 | Pup |  | 1 |
| 5 | BRN | 9 | 2 | 3 | Pup |  | 1 |
| 5 | BRN | 9 | 2 | 1 | Pup | 1 |  |
| 5 | BRN | 9 | 2 | 0.5 | Pup |  | 1 |
| 5 | BRN | 9 | 2 | 0.5 | Pup |  | 1 |
| 5 | BRN | 9 | 2 | 2 | Pup |  | 1 |
| 5 | BRN | 9 | 2 | 2 | Pup |  | 1 |
| 5 | BRN | 9 | 2 | 2 | Pup |  | 1 |
| 5 | BRN | 9 | 2 | 2 | Pup |  | 1 |
| 5 | BRN | 9 | 2 | 2 | Pup |  | 1 |
| 5 | BRN | 9 | 2 | 2 | Pup |  | 1 |
| 5 | BRN | 9 | 2 | 1 | Pup |  | 1 |
| 5 | BRN | 9 | 2 | 1 | Pup |  | 1 |
| 5 | BRN | 9 | 2 | 1 | Pup |  | 1 |
| 5 | BRN | 9 | 2 | 1 | Pup |  | 1 |
| 5 | BRN | 9 | 2 | 1 | Pup |  | 1 |
| 5 | BRN | 9 | 2 | 1 | Pup |  | 1 |
| 5 | BRN | 9 | 2 | 1 | Pup |  | 1 |
| 5 | BRN | 9 | 2 | 1 | Pup |  | 1 |
| 5 | BRN | 9 | 2 | 3 | Pup |  | 1 |
| 5 | BRN | 9 | 2 | 3 | Pup |  | 1 |
| 5 | BRN | 9 | 2 | 2 | Pup |  | 1 |
| 5 | BRN | 9 | 2 | 2 | Pup |  | 1 |
| 5 | BRN | 9 | 2 | 2 | Pup | 1 |  |
| 5 | BRN | 9 | 2 | 2 | Pup | 1 |  |
| 5 | BRN | 9 | 2 | 1 | Pup |  | 1 |
| 5 | BRN | 9 | 2 | 1 | Pup |  | 1 |
| 5 | BRN | 9 | 2 | 1 | Pup | 1 |  |
| 5 | BRN | 9 | 2 | 1 | Pup |  | 1 |
| 5 | BRN | 9 | 2 | 1 | Pup |  | 1 |
| 5 | BRN | 9 | 2 | 1 | Pup |  | 1 |
| 5 | BRN | 9 | 2 | 1 | Pup | 1 |  |
| 5 | BRN | 9 | 2 | 1 | Pup | 1 |  |
| 5 | BRN | 9 | 2 | 2 | Pup | 1 |  |
| 5 | BRN | 9 | 2 | 1 | Pup | 1 |  |
| 5 | KTI | 9 | 2 | 1 | Pup |  | 1 |
| 5 | KTI | 9 | 2 | 1 | Pup |  | 1 |
| 5 | KTI | 9 | 2 | 1 | Pup |  | 1 |
| 5 | KTI | 9 | 2 | 1 | Pup |  | 1 |
| 5 | KTI | 9 | 2 | 0.5 | Pup |  | 1 |
| 5 | KTI | 9 | 2 | 0.5 | Pup |  | 1 |
| 5 | KTI | 9 | 2 | 0.5 | Pup |  | 1 |
| 5 | KTI | 9 | 2 | 1 | Pup |  | 1 |
| 5 | KTI | 9 | 2 | 1 | Pup |  | 1 |
| 5 | KTI | 9 | 2 | 1 | Pup |  | 1 |
| 5 | KTI | 9 | 2 | 0.5 | Pup |  | 1 |
| 5 | KTI | 9 | 2 | 3 | Pup |  | 1 |
| 5 | KTI | 9 | 2 | 2 | Pup |  | 1 |
| 5 | KTI | 9 | 2 | 2 | Pup |  | 1 |
| 5 | KTI | 9 | 2 | 2 | Pup |  | 1 |
| 5 | KTI | 9 | 2 | 2 | Pup |  | 1 |
| 5 | KTI | 9 | 2 | 2 | Pup |  | 1 |
| 5 | KTI | 9 | 2 | 1 | Pup |  | 1 |
| 5 | KTI | 9 | 2 | 3 | Pup |  | 1 |
| 5 | KTI | 9 | 2 | 3 | Pup |  | 1 |
| 5 | KTI | 9 | 2 | 3 | Pup |  | 1 |
| 5 | KTI | 9 | 2 | 1 | Pup |  | 1 |
| 5 | KTI | 9 | 2 | 1 | Pup |  | 1 |
| 5 | KTI | 9 | 2 | 0.5 | Pup |  | 1 |
| 5 | KTI | 9 | 2 | 0.5 | Pup |  | 1 |
| 5 | KTI | 9 | 2 | 0.5 | Pup |  | 1 |
| 5 | KTI | 9 | 2 | 0.5 | Pup | 1 |  |
| 5 | KTI | 9 | 2 | 2 | Pup |  | 1 |
| 5 | KTI | 9 | 2 | 1 | Pup |  | 1 |
| 5 | KTI | 9 | 2 | 1 | Pup |  | 1 |
| 5 | KTI | 9 | 2 | 2 | Pup |  | 1 |
| 5 | KTI | 9 | 2 | 2 | Pup |  | 1 |
| 5 | KTI | 9 | 2 | 2 | Pup |  | 1 |
| 5 | KTI | 9 | 2 | 2 | Pup |  | 1 |
| 5 | KTI | 9 | 2 | 2 | Pup |  | 1 |
| 5 | KTI | 9 | 2 | 2 | Pup |  | 1 |
| 5 | KTI | 9 | 2 | 2 | Pup |  | 1 |
| 5 | KTI | 9 | 2 | 1 | Pup |  | 1 |
| 5 | KTI | 9 | 2 | 1 | Pup |  | 1 |
| 5 | BBR | 9 | 4 | 2 | Pup |  | 1 |
| 5 | BBR | 9 | 4 | 2 | Pup |  | 1 |
| 5 | BBR | 9 | 4 | 2 | Pup |  | 1 |
| 5 | BBR | 9 | 4 | 2 | Pup |  | 1 |
| 5 | BBR | 9 | 4 | 1 | Pup |  | 1 |
| 5 | BBR | 9 | 4 | 1 | Pup |  | 1 |
| 5 | BBR | 9 | 4 | 1 | Pup |  | 1 |
| 5 | BBR | 9 | 4 | 1 | Pup | 1 |  |
| 5 | BBR | 9 | 4 | 1 | Pup | 1 |  |
| 5 | BBR | 9 | 4 | 0.5 | Pup | 1 |  |
| 5 | BBR | 9 | 4 | 1 | Pup | 1 |  |
| 5 | BBR | 9 | 4 | 1 | Pup | 1 |  |
| 5 | BBR | 9 | 4 | 1 | Pup | 1 |  |
| 5 | BBR | 9 | 4 | 1 | Pup | 1 |  |
| 5 | BBR | 9 | 4 | 1 | Pup | 1 |  |
| 5 | BBR | 9 | 4 | 1 | Pup | 1 |  |
| 5 | BBR | 9 | 4 | 2 | Pup | 1 |  |
| 5 | BBR | 9 | 4 | 2 | Pup | 1 |  |
| 5 | BBR | 9 | 4 | 2 | Pup | 1 |  |
| 5 | BBR | 9 | 4 | 1 | Pup | 1 |  |
| 5 | BBR | 9 | 4 | 1 | Pup | 1 |  |
| 5 | BBR | 9 | 4 | 2 | Pup | 1 |  |
| 5 | BBR | 9 | 4 | 4 | Pup | 1 |  |
| 5 | BBR | 9 | 4 | 4 | Pup | 1 |  |
| 5 | BBR | 9 | 4 | 4 | Pup | 1 |  |
| 5 | BBR | 9 | 4 | 4 | Pup | 1 |  |
| 5 | BBR | 9 | 4 | 4 | Pup | 1 |  |
| 5 | BBR | 9 | 4 | 1 | Pup |  | 1 |
| 5 | BBR | 9 | 4 | 1 | Pup |  | 1 |
| 5 | BBR | 9 | 4 | 1 | Pup |  | 1 |
| 5 | BBR | 9 | 4 | 1 | Pup |  | 1 |
| 5 | BBR | 9 | 4 | 1 | Pup | 1 |  |
| 5 | BBR | 9 | 4 | 1 | Pup | 1 |  |
| 5 | BBR | 9 | 4 | 1 | Pup | 1 |  |
| 5 | BBR | 9 | 4 | 1 | Pup | 1 |  |
| 5 | BBR | 9 | 4 | 1 | Pup | 1 |  |
| 5 | BBR | 9 | 4 | 1 | Pup | 1 |  |
| 5 | BBR | 9 | 4 | 2 | Pup | 1 |  |
| 5 | BBR | 9 | 4 | 2 | Pup | 1 |  |
| 5 | BBR | 9 | 4 | 2 | Pup | 1 |  |
| 5 | BBR | 9 | 4 | 2 | Pup | 1 |  |
| 5 | BBR | 9 | 4 | 1 | Pup | 1 |  |
| 5 | BBR | 9 | 4 | 1 | Pup | 1 |  |
| 5 | BBR | 9 | 4 | 1 | Pup | 1 |  |
| 5 | WHI | 9 | 2 | 1 | Pup |  | 1 |
| 5 | WHI | 9 | 2 | 1 | Pup |  | 1 |
| 5 | WHI | 9 | 2 | 0.5 | Pup | 1 |  |
| 5 | WHI | 9 | 2 | 1 | Pup | 1 |  |
| 5 | WHI | 9 | 2 | 1 | Pup | 1 |  |
| 5 | WHI | 9 | 2 | 1 | Pup | 1 |  |
| 5 | WHI | 9 | 2 | 1 | Pup | 1 |  |
| 5 | WHI | 9 | 2 | 1 | Pup | 1 |  |
| 5 | WHI | 9 | 2 | 5 | Pup |  | 1 |
| 5 | WHI | 9 | 2 | 4 | Pup |  | 1 |
| 5 | WHI | 9 | 2 | 4 | Pup |  | 1 |
| 5 | WHI | 9 | 2 | 5 | Pup |  | 1 |
| 5 | WHI | 9 | 2 | 5 | Pup |  | 1 |
| 5 | WHI | 9 | 2 | 5 | Pup |  | 1 |
| 5 | WHI | 9 | 2 | 1 | Pup |  | 1 |
| 5 | WHI | 9 | 2 | 3 | Pup |  | 1 |
| 5 | WHI | 9 | 2 | 2 | Pup |  | 1 |
| 5 | WHI | 9 | 2 | 1 | Pup |  | 1 |
| 5 | WHI | 9 | 2 | 1 | Pup |  | 1 |
| 5 | WHI | 9 | 2 | 1 | Pup |  | 1 |
| 5 | WHI | 9 | 2 | 1 | Pup |  | 1 |
| 5 | WHI | 9 | 2 | 1 | Pup |  | 1 |
| 5 | WHI | 9 | 2 | 2 | Pup |  | 1 |
| 5 | WHI | 9 | 2 | 2 | Pup |  | 1 |
| 5 | WHI | 9 | 2 | 2 | Pup |  | 1 |
| 5 | WHI | 9 | 2 | 0.5 | Pup |  | 1 |
| 5 | WHI | 9 | 2 | 0.5 | Pup |  | 1 |
| 5 | WHI | 9 | 2 | 0.5 | Pup |  | 1 |
| 5 | WHI | 9 | 2 | 1 | Pup |  | 1 |
| 5 | WHI | 9 | 2 | 1 | Pup |  | 1 |
| 5 | WHI | 9 | 2 | 1 | Pup |  | 1 |
| 5 | WHI | 9 | 2 | 1 | Pup |  | 1 |
| 5 | WHI | 9 | 2 | 1 | Pup |  | 1 |
| 5 | WHI | 9 | 2 | 1 | Pup |  | 1 |
| 5 | WHI | 9 | 2 | 1 | Pup |  | 1 |
| 5 | WHI | 9 | 2 | 1 | Pup |  | 1 |
| 5 | WHI | 9 | 2 | 1 | Pup |  | 1 |
| 5 | WHI | 9 | 2 | 1 | Pup |  | 1 |
| 5 | WHI | 9 | 2 | 1 | Pup |  | 1 |
| 5 | WHI | 9 | 2 | 2 | Pup |  | 1 |
| 5 | WHI | 9 | 2 | 2 | Pup |  | 1 |
| 5 | WHI | 9 | 2 | 2 | Pup |  | 1 |
| 5 | WHI | 9 | 2 | 2 | Pup | 1 |  |
| 5 | WHI | 9 | 2 | 1 | Pup | 1 |  |
| 5 | WHI | 9 | 2 | 2 | Pup | 1 |  |
| 5 | WHI | 9 | 2 | 0.5 | Pup |  | 1 |
| 5 | WHI | 9 | 2 | 1 | Pup |  | 1 |
| 5 | WHI | 9 | 2 | 1 | Pup |  | 1 |
| 5 | WHI | 9 | 2 | 3 | Pup |  | 1 |
| 5 | WHI | 9 | 2 | 3 | Pup |  | 1 |
| 5 | WHI | 9 | 2 | 3 | Pup |  | 1 |
| 5 | WHI | 9 | 2 | 1 | Pup |  | 1 |
| 5 | WHI | 9 | 2 | 3 | Pup |  | 1 |
| 5 | WHI | 9 | 2 | 3 | Pup |  | 1 |
| 5 | WHI | 9 | 2 | 1 | Pup | 1 |  |
| 5 | WHI | 9 | 2 | 1 | Pup | 1 |  |
| 5 | WHI | 9 | 2 | 1 | Pup | 1 |  |
| 5 | WHI | 9 | 2 | 0.5 | Pup | 1 |  |
| 5 | WHI | 9 | 2 | 0.5 | Pup | 1 |  |
| 5 | WHI | 9 | 2 | 0.5 | Pup | 1 |  |
| 5 | WHI | 9 | 2 | 1 | Pup |  | 1 |
| 5 | WHI | 9 | 2 | 0.5 | Pup |  | 1 |
| 5 | WHI | 9 | 2 | 0.5 | Pup |  | 1 |
| 5 | WHI | 9 | 2 | 3 | Pup | 1 |  |
| 5 | WHI | 9 | 2 | 2 | Pup | 1 |  |
| 5 | WHI | 9 | 2 | 0.5 | Pup |  | 1 |
| 5 | WHI | 9 | 2 | 0.5 | Pup |  | 1 |
| 5 | WHI | 9 | 2 | 2 | Pup |  | 1 |
| 5 | WHI | 9 | 2 | 2 | Pup |  | 1 |
| 4 | CAN | 10 | 5 | 2 | MO | 1 |  |
| 4 | CAN | 10 | 5 | 2 | MO | 1 |  |
| 4 | CAN | 10 | 5 | 2 | MO | 1 |  |
| 4 | CAN | 10 | 5 | 2 | MO | 1 |  |
| 4 | CAN | 10 | 5 | 2 | MO | 1 |  |
| 4 | CAN | 10 | 5 | 2 | Pup | 1 |  |
| 4 | CAN | 10 | 5 | 2 | Pup | 1 |  |
| 4 | CAN | 10 | 5 | 2 | Pup | 1 |  |
| 4 | CAN | 10 | 5 | 2 | Pup | 1 |  |
| 4 | CAN | 10 | 5 | 2 | Pup | 1 |  |
| 4 | CAN | 10 | 5 | 0.5 | Pup | 1 |  |
| 4 | CAN | 10 | 5 | 0.5 | Pup | 1 |  |
| 4 | CAN | 10 | 5 | 0.5 | Pup | 1 |  |
| 4 | CAN | 10 | 5 | 0.5 | Pup | 1 |  |
| 4 | CAN | 10 | 5 | 2 | Pup | 1 |  |
| 4 | CAN | 10 | 5 | 2 | Pup | 1 |  |
| 4 | CAN | 10 | 5 | 2 | Pup | 1 |  |
| 4 | CAN | 10 | 5 | 2 | Pup | 1 |  |
| 4 | CAN | 10 | 5 | 3 | Pup | 1 |  |
| 4 | CAN | 10 | 5 | 3 | Pup | 1 |  |
| 4 | CAN | 10 | 5 | 3 | Pup | 1 |  |
| 4 | CAN | 10 | 5 | 3 | Pup | 1 |  |
| 4 | CAN | 10 | 5 | 0.5 | Pup | 1 |  |
| 4 | CAN | 10 | 5 | 0.5 | Pup | 1 |  |
| 4 | CAN | 10 | 5 | 0.5 | Pup | 1 |  |
| 4 | CAN | 10 | 5 | 0.5 | Pup | 1 |  |
| 4 | CAN | 10 | 5 | 1 | Pup | 1 |  |
| 4 | CAN | 10 | 5 | 1 | Pup | 1 |  |
| 4 | CAN | 10 | 5 | 1 | Pup | 1 |  |
| 4 | CAN | 10 | 5 | 1 | Pup | 1 |  |
| 4 | CAN | 10 | 5 | 0.5 | Pup | 1 |  |
| 4 | CAN | 10 | 5 | 0.5 | Pup | 1 |  |
| 4 | CAN | 10 | 5 | 0.5 | Pup | 1 |  |
| 4 | CAN | 10 | 5 | 0.5 | Pup | 1 |  |
| 4 | CAN | 10 | 5 | 4 | Pup | 1 |  |
| 4 | CAN | 10 | 5 | 4 | Pup | 1 |  |
| 4 | CAN | 10 | 5 | 4 | Pup | 1 |  |
| 4 | CAN | 10 | 5 | 3 | Pup | 1 |  |
| 4 | CAN | 10 | 5 | 2 | Pup | 1 |  |
| 4 | CAN | 10 | 5 | 2 | Pup | 1 |  |
| 4 | CAN | 10 | 5 | 2 | Pup | 1 |  |
| 4 | PF1 | 10 | 2 | 1 | Pup | 1 |  |
| 4 | PF1 | 10 | 2 | 1 | Pup | 1 |  |
| 4 | PF1 | 10 | 2 | 1 | Pup | 1 |  |
| 4 | PF1 | 10 | 2 | 1 | Pup | 1 |  |
| 4 | RS2 | 10 | 2 | 2 | Pup | 1 |  |
| 4 | RS2 | 10 | 2 | 0.5 | Pup | 1 |  |
| 4 | RS2 | 10 | 2 | 0.5 | Pup | 1 |  |
| 4 | RS3 | 10 | 1 | 1 | Pup | 1 |  |
| 4 | RS3 | 10 | 1 | 1 | Pup | 1 |  |
| 4 | RS3 | 10 | 1 | 0.5 | Pup | 1 |  |
| 5 | BRN | 10 | 2 | 1 | Pup | 1 |  |
| 5 | BRN | 10 | 2 | 1 | Pup |  | 1 |
| 5 | BRN | 10 | 2 | 1 | Pup |  | 1 |
| 5 | BRN | 10 | 2 | 1 | Pup |  | 1 |
| 5 | BRN | 10 | 2 | 1 | Pup |  | 1 |
| 5 | BRN | 10 | 2 | 0.5 | Pup |  | 1 |
| 5 | BRN | 10 | 2 | 2 | Pup |  | 1 |
| 5 | BRN | 10 | 2 | 2 | Pup |  | 1 |
| 5 | BRN | 10 | 2 | 2 | Pup |  | 1 |
| 5 | BRN | 10 | 2 | 2 | Pup |  | 1 |
| 5 | BRN | 10 | 2 | 1 | Pup |  | 1 |
| 5 | BRN | 10 | 2 | 1 | Pup |  | 1 |
| 5 | BRN | 10 | 2 | 0.5 | Pup |  | 1 |
| 5 | BRN | 10 | 2 | 0.5 | Pup |  | 1 |
| 5 | BRN | 10 | 2 | 1 | Pup |  | 1 |
| 5 | BRN | 10 | 2 | 1 | Pup |  | 1 |
| 5 | BRN | 10 | 2 | 1 | Pup |  | 1 |
| 5 | BRN | 10 | 2 | 1 | Pup |  | 1 |
| 5 | BRN | 10 | 2 | 1 | Pup | 1 |  |
| 5 | BRN | 10 | 2 | 2 | Pup | 1 |  |
| 5 | BRN | 10 | 2 | 1 | Pup | 1 |  |
| 5 | BRN | 10 | 2 | 1 | Pup |  | 1 |
| 5 | BRN | 10 | 2 | 1 | Pup |  | 1 |
| 5 | BRN | 10 | 2 | 1 | Pup |  | 1 |
| 5 | BRN | 10 | 2 | 1 | Pup |  | 1 |
| 5 | BRN | 10 | 2 | 1 | Pup |  | 1 |
| 5 | BRN | 10 | 2 | 1 | Pup |  | 1 |
| 5 | BRN | 10 | 2 | 0.5 | Pup |  | 1 |
| 5 | BRN | 10 | 2 | 1 | Pup |  | 1 |
| 5 | BRN | 10 | 2 | 1 | Pup |  | 1 |
| 5 | KTI | 10 | 2 | 2 | Pup |  | 1 |
| 5 | KTI | 10 | 2 | 1 | Pup |  | 1 |
| 5 | KTI | 10 | 2 | 1 | Pup |  | 1 |
| 5 | KTI | 10 | 2 | 1 | Pup |  | 1 |
| 5 | KTI | 10 | 2 | 1 | Pup |  | 1 |
| 5 | KTI | 10 | 2 | 1 | Pup |  | 1 |
| 5 | BBR | 10 | 4 | 1 | Pup | 1 |  |
| 5 | BBR | 10 | 4 | 1 | Pup | 1 |  |
| 5 | BBR | 10 | 4 | 1 | Pup | 1 |  |
| 5 | BBR | 10 | 4 | 2 | Pup | 1 |  |
| 5 | BBR | 10 | 4 | 2 | Pup | 1 |  |
| 5 | BBR | 10 | 4 | 0.5 | Pup | 1 |  |
| 5 | BBR | 10 | 4 | 0.5 | Pup | 1 |  |
| 5 | BBR | 10 | 4 | 1 | Pup | 1 |  |
| 5 | BBR | 10 | 4 | 1 | Pup | 1 |  |
| 5 | BBR | 10 | 4 | 0.5 | Pup | 1 |  |
| 5 | BBR | 10 | 4 | 0.5 | Pup | 1 |  |
| 5 | BBR | 10 | 4 | 1 | Pup | 1 |  |
| 5 | BBR | 10 | 4 | 1 | Pup | 1 |  |
| 5 | BBR | 10 | 4 | 1 | Pup | 1 |  |
| 5 | BBR | 10 | 4 | 1 | Pup | 1 |  |
| 5 | BBR | 10 | 4 | 0.5 | Pup | 1 |  |
| 5 | BBR | 10 | 4 | 0.5 | Pup | 1 |  |
| 5 | WHI | 10 | 2 | 1 | Pup | 1 |  |
| 5 | WHI | 10 | 2 | 4 | Pup | 1 |  |
| 5 | WHI | 10 | 2 | 3 | Pup | 1 |  |
| 5 | WHI | 10 | 2 | 3 | Pup | 1 |  |
| 5 | WHI | 10 | 2 | 2 | Pup |  | 1 |
| 5 | WHI | 10 | 2 | 2 | Pup |  | 1 |
| 5 | WHI | 10 | 2 | 0.5 | Pup |  | 1 |
| 5 | WHI | 10 | 2 | 0.5 | Pup |  | 1 |
| 5 | WHI | 10 | 2 | 0.5 | Pup |  | 1 |
| 5 | WHI | 10 | 2 | 0.5 | Pup |  | 1 |
| 5 | WHI | 10 | 2 | 1 | Pup | 1 |  |
| 5 | WHI | 10 | 2 | 1 | Pup |  | 1 |
| 5 | WHI | 10 | 2 | 1 | Pup |  | 1 |
| 5 | WHI | 10 | 2 | 1 | Pup |  | 1 |
| 5 | WHI | 10 | 2 | 1 | Pup |  | 1 |
| 5 | WHI | 10 | 2 | 1 | Pup |  | 1 |
| 5 | WHI | 10 | 2 | 1 | Pup |  | 1 |
| 5 | WHI | 10 | 2 | 5 | Pup | 1 |  |
| 5 | WHI | 10 | 2 | 1 | Pup |  | 1 |
| 5 | WHI | 10 | 2 | 1 | Pup |  | 1 |
| 5 | WHI | 10 | 2 | 1 | Pup |  | 1 |
| 5 | WHI | 10 | 2 | 1 | Pup |  | 1 |
| 5 | WHI | 10 | 2 | 1 | Pup |  | 1 |
| 5 | WHI | 10 | 2 | 1 | Pup |  | 1 |
| 5 | WHI | 10 | 2 | 1 | Pup |  | 1 |
| 5 | WHI | 10 | 2 | 1 | Pup |  | 1 |
| 5 | WHI | 10 | 2 | 0.5 | Pup |  | 1 |
| 5 | WHI | 10 | 2 | 0.5 | Pup |  | 1 |
| 5 | WHI | 10 | 2 | 1 | Pup |  | 1 |
| 5 | WHI | 10 | 2 | 1 | Pup |  | 1 |
| 5 | WHI | 10 | 2 | 0.5 | Pup |  | 1 |
| 5 | WHI | 10 | 2 | 0.5 | Pup |  | 1 |
| 5 | WHI | 10 | 2 | 1 | Pup |  | 1 |
| 5 | WHI | 10 | 2 | 1 | Pup |  | 1 |
| 5 | WHI | 10 | 2 | 1 | Pup |  | 1 |
| 5 | WHI | 10 | 2 | 1 | Pup |  | 1 |
| 5 | WHI | 10 | 2 | 1 | Pup |  | 1 |
| 5 | WHI | 10 | 2 | 1 | Pup |  | 1 |
| 5 | WHI | 10 | 2 | 0.5 | Pup |  | 1 |
| 5 | WHI | 10 | 2 | 0.5 | Pup |  | 1 |
| 5 | WHI | 10 | 2 | 0.5 | Pup |  | 1 |
| 5 | WHI | 10 | 2 | 0.5 | Pup |  | 1 |
| 5 | WHI | 10 | 2 | 0.5 | Pup |  | 1 |
| 5 | WHI | 10 | 2 | 0.5 | Pup |  | 1 |
| 5 | WHI | 10 | 2 | 1 | Pup | 1 |  |
| 5 | WHI | 10 | 2 | 1 | Pup | 1 |  |
| 5 | WHI | 10 | 2 | 1 | Pup | 1 |  |
| 5 | WHI | 10 | 2 | 1 | Pup | 1 |  |
| 5 | WHI | 10 | 2 | 1 | Pup | 1 |  |
| 5 | WHI | 10 | 2 | 1 | Pup | 1 |  |
| 5 | WHI | 10 | 2 | 1 | Pup | 1 |  |
| 5 | WHI | 10 | 2 | 1 | Pup | 1 |  |
| 5 | WHI | 10 | 2 | 1 | Pup | 1 |  |
| 5 | WHI | 10 | 2 | 1 | Pup | 1 |  |
| 5 | WHI | 10 | 2 | 1 | Pup |  | 1 |
| 5 | WHI | 10 | 2 | 1 | Pup |  | 1 |
| 4 | CAN | 11 | 5 | 0.5 | Pup | 1 |  |
| 4 | CAN | 11 | 5 | 2 | Pup | 1 |  |
| 4 | CAN | 11 | 5 | 2 | Pup | 1 |  |
| 4 | CAN | 11 | 5 | 2 | Pup | 1 |  |
| 4 | CAN | 11 | 5 | 2 | Pup | 1 |  |
| 4 | CAN | 11 | 5 | 2 | Pup | 1 |  |
| 4 | CAN | 11 | 5 | 3 | Pup | 1 |  |
| 4 | CAN | 11 | 5 | 2 | Pup | 1 |  |
| 4 | CAN | 11 | 5 | 0.5 | Pup | 1 |  |
| 4 | RS2 | 11 | 2 | 1 | Pup | 1 |  |
| 4 | RS2 | 11 | 2 | 0.5 | Pup | 1 |  |
| 4 | RS2 | 11 | 2 | 2 | Pup | 1 |  |
| 4 | RS2 | 11 | 2 | 1 | Pup | 1 |  |
| 4 | RS2 | 11 | 2 | 2 | Pup | 1 |  |
| 4 | RS3 | 11 | 1 | 4 | Pup | 1 |  |
| 4 | RS3 | 11 | 1 | 5 | Pup | 1 |  |
| 5 | BRN | 11 | 1 | 3 | Pup |  | 1 |
| 5 | BRN | 11 | 1 | 3 | Pup |  | 1 |
| 5 | BRN | 11 | 1 | 0.5 | Pup |  | 1 |
| 5 | BRN | 11 | 1 | 0.5 | Pup |  | 1 |
| 5 | BRN | 11 | 1 | 2 | Pup |  | 1 |
| 5 | BRN | 11 | 1 | 2 | Pup |  | 1 |
| 5 | KTI | 11 | 2 | 1 | Pup |  | 1 |
| 5 | KTI | 11 | 2 | 1 | Pup |  | 1 |
| 5 | KTI | 11 | 2 | 2 | Pup |  | 1 |
| 5 | KTI | 11 | 2 | 2 | Pup |  | 1 |
| 5 | KTI | 11 | 2 | 2 | Pup |  | 1 |
| 5 | KTI | 11 | 2 | 1 | Pup |  | 1 |
| 5 | KTI | 11 | 2 | 1 | Pup |  | 1 |
| 5 | WHI | 11 | 2 | 1 | Pup |  | 1 |
| 5 | WHI | 11 | 2 | 1 | Pup |  | 1 |
| 5 | WHI | 11 | 2 | 1 | Pup |  | 1 |
| 5 | WHI | 11 | 2 | 2 | Pup |  | 1 |
| 5 | WHI | 11 | 2 | 2 | Pup |  | 1 |
| 5 | WHI | 11 | 2 | 2 | Pup |  | 1 |
| 5 | WHI | 11 | 2 | 0.5 | Pup |  | 1 |
| 5 | WHI | 11 | 2 | 0.5 | Pup |  | 1 |
| 5 | WHI | 11 | 2 | 0.5 | Pup |  | 1 |
| 5 | WHI | 11 | 2 | 0.5 | Pup |  | 1 |
| 5 | WHI | 11 | 2 | 2 | Pup |  | 1 |
| 5 | WHI | 11 | 2 | 2 | Pup |  | 1 |
| 5 | WHI | 11 | 2 | 1 | Pup |  | 1 |
| 5 | WHI | 11 | 2 | 2 | Pup |  | 1 |
| 5 | WHI | 11 | 2 | 2 | Pup |  | 1 |
| 5 | WHI | 11 | 2 | 2 | Pup |  | 1 |
| 5 | WHI | 11 | 2 | 2 | Pup |  | 1 |
| 5 | WHI | 11 | 2 | 2 | Pup |  | 1 |
| 5 | WHI | 11 | 2 | 0.5 | Pup |  | 1 |
| 5 | WHI | 11 | 2 | 0.5 | Pup |  | 1 |
| 5 | WHI | 11 | 2 | 0.5 | Pup |  | 1 |
| 5 | WHI | 11 | 2 | 0.5 | Pup |  | 1 |
| 5 | WHI | 11 | 2 | 0.5 | Pup |  | 1 |
| 4 | CAN | 12 | 5 | 2 | Pup | 1 |  |
| 4 | CAN | 12 | 5 | 2 | Pup | 1 |  |
| 4 | CAN | 12 | 5 | 2 | Pup | 1 |  |
| 4 | RS2 | 12 | 2 | 0.5 | Pup | 1 |  |
| 4 | RS2 | 12 | 2 | 0.5 | Pup | 1 |  |
| 4 | RS3 | 12 | 1 | 1 | Pup | 1 |  |
| 4 | RS3 | 12 | 1 | 3 | Pup | 1 |  |
| 5 | KTI | 12 | 2 | 1 | Pup |  | 1 |
| 5 | KTI | 12 | 2 | 0.5 | Pup |  | 1 |
| 5 | WHI | 12 | 2 | 2 | Pup |  | 1 |
| 5 | WHI | 12 | 2 | 2 | Pup |  | 1 |
| 5 | WHI | 12 | 2 | 1 | Pup |  | 1 |
| 5 | WHI | 12 | 2 | 1 | Pup |  | 1 |
| 5 | WHI | 12 | 2 | 1 | Pup |  | 1 |
| 5 | WHI | 12 | 2 | 1 | Pup |  | 1 |
| 5 | WHI | 12 | 2 | 1 | Pup |  | 1 |
| 5 | WHI | 12 | 2 | 1 | Pup |  | 1 |
| 5 | WHI | 12 | 2 | 1 | Pup |  | 1 |
| 5 | WHI | 12 | 2 | 2 | Pup |  | 1 |
| 5 | WHI | 12 | 2 | 1 | Pup |  | 1 |
| 5 | WHI | 12 | 2 | 1 | Pup |  | 1 |
| 5 | WHI | 12 | 2 | 1 | Pup |  | 1 |
| 5 | WHI | 12 | 2 | 0.5 | Pup |  | 1 |
| 5 | WHI | 12 | 2 | 1 | Pup |  | 1 |
| 5 | WHI | 12 | 2 | 1 | Pup |  | 1 |
| 5 | WHI | 12 | 2 | 1 | Pup |  | 1 |
| 5 | WHI | 12 | 2 | 1 | Pup |  | 1 |
| 5 | WHI | 12 | 2 | 0.5 | Pup |  | 1 |
| 5 | WHI | 12 | 2 | 0.5 | Pup |  | 1 |
| 5 | WHI | 12 | 2 | 0.5 | Pup |  | 1 |
| 5 | WHI | 12 | 2 | 1 | Pup |  | 1 |
| 5 | WHI | 12 | 2 | 1 | Pup |  | 1 |
| 5 | WHI | 12 | 2 | 2 | Pup |  | 1 |
| 5 | WHI | 12 | 2 | 2 | Pup |  | 1 |
| 4 | CAN | 13 | 5 | 1 | Pup | 1 |  |
| 4 | CAN | 13 | 5 | 1 | Pup | 1 |  |
| 4 | CAN | 13 | 5 | 0.5 | Pup | 1 |  |
| 4 | CAN | 13 | 5 | 0.5 | Pup | 1 |  |
| 4 | CAN | 14 |  | 0 |  |  |  |
| 4 | CAN | 15 |  | 0 |  |  |  |
| 4 | CAN | 16 |  | 0 |  |  |  |
| 4 | CAN | 17 |  | 0 |  |  |  |
| 4 | CAN | 14 |  | 0 |  |  |  |
| 4 | CAN | 15 |  | 0 |  |  |  |
| 4 | CAN | 16 |  | 0 |  |  |  |
| 4 | CAN | 17 |  | 0 |  |  |  |

**Raw data for Model 2**

| ID | Current | Age | Initiated by | |
| --- | --- | --- | --- | --- |
|  | litter size |  | mother | pup |
| CAN | 6 | 3 | 18 | 2 |
| CAN | 6 | 4 | 18 | 0 |
| CAN | 6 | 5 | 24 | 16 |
| CAN | 6 | 6 | 7 | 4 |
| CAN | 5 | 7 | 10 | 5 |
| CAN | 5 | 8 | 8 | 13 |
| CAN | 5 | 9 |  |  |
| CAN | 5 | 10 | 5 | 36 |
| CAN | 5 | 11 | 0 | 9 |
| CAN | 5 | 12 | 0 | 3 |
| CAN | 5 | 13 | 0 | 4 |
| CAN | 5 | 14 |  |  |
| CAN | 5 | 15 |  |  |
| MDB | 5 | 3 | 15 | 0 |
| MDB | 5 | 4 | 22 | 8 |
| MDB | 5 | 5 |  |  |
| MDB | 5 | 6 | 10 | 29 |
| MDB | 5 | 7 |  |  |
| PF1 | 5 | 3 | 30 | 12 |
| PF1 | 5 | 4 | 0 | 39 |
| PF1 | 4 | 5 | 0 | 29 |
| PF1 | 3 | 6 | 0 | 16 |
| PF1 | 3 | 7 | 0 | 7 |
| PF1 | 3 | 8 | 0 | 6 |
| PF1 | 2 | 9 | 0 | 3 |
| PF1 | 2 | 10 | 0 | 4 |
| PF1 | 2 | 11 |  |  |
| PF1 | 2 | 12 |  |  |
| PF1 | 2 | 13 |  |  |
| RS1 | 2 | 3 | 10 | 18 |
| RS1 | 2 | 4 | 10 | 8 |
| RS1 | 2 | 5 | 2 | 3 |
| RS1 | 2 | 6 | 2 | 5 |
| RS1 | 2 | 7 | 0 | 96 |
| RS1 | 2 | 8 | 0 | 32 |
| RS1 | 2 | 9 | 0 | 30 |
| RS2 | 4 | 3 | 37 | 13 |
| RS2 | 4 | 4 | 12 | 22 |
| RS2 | 4 | 5 | 28 | 1 |
| RS2 | 4 | 6 | 8 | 73 |
| RS2 | 2 | 7 | 0 | 15 |
| RS2 | 2 | 8 |  |  |
| RS2 | 2 | 9 | 0 | 61 |
| RS2 | 2 | 10 | 0 | 3 |
| RS2 | 2 | 11 | 0 | 5 |
| RS2 | 2 | 12 | 0 | 2 |
| RS2 | 2 | 13 |  |  |
| RS2 | 2 | 14 |  |  |
| RS3 | 2 | 3 | 5 | 20 |
| RS3 | 2 | 4 | 4 | 10 |
| RS3 | 2 | 5 | 8 | 11 |
| RS3 | 2 | 6 | 2 | 8 |
| RS3 | 2 | 7 | 4 | 7 |
| RS3 | 1 | 8 | 0 | 11 |
| RS3 | 1 | 9 | 0 | 3 |
| RS3 | 1 | 10 | 0 | 3 |
| RS3 | 1 | 11 | 0 | 2 |
| RS3 | 1 | 12 | 0 | 2 |
| RS3 | 1 | 13 |  |  |
| BRN | 2 | 3 | 5 | 22 |
| BRN | 2 | 4 | 2 | 12 |
| BRN | 2 | 5 | 0 | 12 |
| BRN | 1 | 6 | 0 | 12 |
| BRN | 2 | 7 | 0 | 41 |
| BRN | 2 | 8 | 0 | 13 |
| BRN | 2 | 9 | 0 | 44 |
| BRN | 2 | 10 | 0 | 17 |
| BRN | 1 | 11 | 0 | 3 |
| KTI | 2 | 3 | 6 | 10 |
| KTI | 2 | 4 | 14 | 26 |
| KTI | 2 | 5 | 14 | 25 |
| KTI | 2 | 6 | 0 | 16 |
| KTI | 2 | 7 | 0 | 22 |
| KTI | 2 | 8 | 0 | 15 |
| KTI | 2 | 9 | 0 | 25 |
| KTI | 2 | 10 | 0 | 3 |
| KTI | 2 | 11 | 0 | 4 |
| KTI | 2 | 12 | 0 | 2 |
| BBR | 4 | 3 | 30 | 19 |
| BBR | 4 | 4 | 24 | 6 |
| BBR | 4 | 5 | 24 | 14 |
| BBR | 4 | 6 | 12 | 29 |
| BBR | 4 | 7 | 0 | 62 |
| BBR | 4 | 8 | 0 | 37 |
| BBR | 4 | 9 | 0 | 30 |
| BBR | 4 | 10 | 0 | 11 |
| WHI | 2 | 3 | 9 | 8 |
| WHI | 2 | 4 | 10 | 43 |
| WHI | 2 | 5 | 12 | 12 |
| WHI | 2 | 6 | 10 | 11 |
| WHI | 2 | 7 | 8 | 17 |
| WHI | 2 | 8 | 0 | 35 |
| WHI | 2 | 9 | 0 | 50 |
| WHI | 2 | 10 | 0 | 37 |
| WHI | 2 | 11 | 0 | 15 |
| WHI | 2 | 12 | 0 | 16 |
| RS5 | 3 | 3 | 15 | 22 |
| RS5 | 3 | 4 | 11 | 7 |
| RS5 | 3 | 5 | 12 | 15 |
| RS5 | 3 | 6 | 3 | 22 |
| RS5 | 3 | 7 | 3 | 13 |

**Raw data for Model 3**

| ID | Current | Age | Termination of nursing/ suckling bouts through refusal | |
| --- | --- | --- | --- | --- |
|  | litter size |  | Mother mediated | Allomother mediated |
| CAN | 6 | 3 | 0.1 | 0 |
| CAN | 6 | 4 | 0.333333333 |  |
| CAN | 6 | 5 | 0.1 |  |
| CAN | 6 | 6 | 0.181818182 |  |
| CAN | 5 | 7 | 1 |  |
| CAN | 5 | 8 | 1 |  |
| CAN | 5 | 9 |  |  |
| CAN | 5 | 10 | 1 |  |
| CAN | 5 | 11 | 1 |  |
| CAN | 5 | 12 | 1 |  |
| CAN | 5 | 13 | 1 |  |
| CAN | 5 | 14 |  |  |
| CAN | 5 | 15 |  |  |
| MDB | 5 | 3 | 0.666666667 |  |
| MDB | 5 | 4 | 0.533333333 |  |
| MDB | 5 | 5 |  |  |
| MDB | 5 | 6 | 1 |  |
| MDB | 5 | 7 |  |  |
| PF1 | 5 | 3 | 0.380952381 |  |
| PF1 | 5 | 4 | 1 | 0 |
| PF1 | 4 | 5 | 1 |  |
| PF1 | 3 | 6 | 0.5 | 0 |
| PF1 | 3 | 7 | 1 | 1 |
| PF1 | 3 | 8 | 0.333333333 |  |
| PF1 | 2 | 9 | 1 | 1 |
| PF1 | 2 | 10 | 1 |  |
| PF1 | 2 | 11 |  |  |
| PF1 | 2 | 12 |  |  |
| PF1 | 2 | 13 |  |  |
| RS1 | 2 | 3 | 0.357142857 |  |
| RS1 | 2 | 4 | 0.277777778 |  |
| RS1 | 2 | 5 | 0.6 |  |
| RS1 | 2 | 6 | 1 |  |
| RS1 | 2 | 7 | 1 | 0.890410959 |
| RS1 | 2 | 8 | 1 | 0.954545455 |
| RS1 | 2 | 9 | 0.8 | 0.892857143 |
| RS2 | 4 | 3 | 0.8 |  |
| RS2 | 4 | 4 | 0.828571429 |  |
| RS2 | 4 | 5 | 0.651515152 |  |
| RS2 | 4 | 6 | 0.879310345 | 1 |
| RS2 | 2 | 7 | 0.96969697 | 1 |
| RS2 | 2 | 8 |  |  |
| RS2 | 2 | 9 | 0.833333333 | 1 |
| RS2 | 2 | 10 | 0.666666667 |  |
| RS2 | 2 | 11 | 0.6 |  |
| RS2 | 2 | 12 | 1 |  |
| RS2 | 2 | 13 |  |  |
| RS2 | 2 | 14 |  |  |
| RS3 | 2 | 3 | 0 |  |
| RS3 | 2 | 4 | 0.428571429 |  |
| RS3 | 2 | 5 | 0.578947368 |  |
| RS3 | 2 | 6 | 0.666666667 | 1 |
| RS3 | 2 | 7 | 0.642857143 |  |
| RS3 | 1 | 8 | 0.923076923 | 1 |
| RS3 | 1 | 9 | 1 | 1 |
| RS3 | 1 | 10 | 1 |  |
| RS3 | 1 | 11 | 0.5 |  |
| RS3 | 1 | 12 | 0.5 |  |
| RS3 | 1 | 13 |  |  |
| BRN | 2 | 3 | 0.296296296 |  |
| BRN | 2 | 4 | 1 |  |
| BRN | 2 | 5 | 0.818181818 | 1 |
| BRN | 1 | 6 | 0.705882353 |  |
| BRN | 2 | 7 | 0.847826087 | 1 |
| BRN | 2 | 8 | 1 | 1 |
| BRN | 2 | 9 | 0.866666667 | 0.962962963 |
| BRN | 2 | 10 | 1 | 1 |
| BRN | 1 | 11 |  | 1 |
| KTI | 2 | 3 | 0.4375 |  |
| KTI | 2 | 4 | 0.1 |  |
| KTI | 2 | 5 | 0.230769231 |  |
| KTI | 2 | 6 | 1 | 1 |
| KTI | 2 | 7 | 1 | 1 |
| KTI | 2 | 8 | 1 | 1 |
| KTI | 2 | 9 | 1 | 1 |
| KTI | 2 | 10 |  | 1 |
| KTI | 2 | 11 |  | 1 |
| KTI | 2 | 12 |  | 1 |
| BBR | 4 | 3 | 0.489795918 |  |
| BBR | 4 | 4 | 0.575757576 |  |
| BBR | 4 | 5 | 0.714285714 |  |
| BBR | 4 | 6 | 0.852941176 | 1 |
| BBR | 4 | 7 | 1 | 1 |
| BBR | 4 | 8 | 1 | 1 |
| BBR | 4 | 9 | 1 | 1 |
| BBR | 4 | 10 | 1 |  |
| WHI | 2 | 3 | 0.352941176 |  |
| WHI | 2 | 4 | 0.132075472 |  |
| WHI | 2 | 5 | 0.166666667 |  |
| WHI | 2 | 6 | 0.238095238 |  |
| WHI | 2 | 7 | 0.913043478 | 1 |
| WHI | 2 | 8 | 1 | 1 |
| WHI | 2 | 9 | 1 | 0.980769231 |
| WHI | 2 | 10 | 0.875 | 1 |
| WHI | 2 | 11 |  | 1 |
| WHI | 2 | 12 |  | 1 |
| RS5 | 3 | 3 | 0.081081081 |  |
| RS5 | 3 | 4 | 0.166666667 |  |
| RS5 | 3 | 5 | 0 |  |
| RS5 | 3 | 6 | 0.56 |  |
| RS5 | 3 | 7 | 1 | 1 |
| RS5 | 3 | 8 |  |  |
| RS5 | 3 | 9 |  |  |
| RS5 | 3 | 10 |  |  |
| RS5 | 3 | 11 |  |  |
| RS5 | 3 | 12 |  |  |
| RS5 | 3 | 13 |  |  |
| RS5 | 3 | 14 |  |  |
| RS5 | 3 | 15 |  |  |
| RS5 | 3 | 16 |  |  |
| RS5 | 3 | 17 |  |  |
